# A new Middle Jurassic lagoon margin assemblage of theropod and sauropod dinosaur trackways from the Isle of Skye, Scotland

**DOI:** 10.1371/journal.pone.0319862

**Published:** 2025-04-02

**Authors:** Tone Blakesley, Paige E. dePolo, Thomas J. Wade, Dugald A. Ross, Stephen L. Brusatte

**Affiliations:** 1 School of GeoSciences, University of Edinburgh, Edinburgh, United Kingdom; 2 School of Biological and Environmental Sciences, Liverpool John Moores University, Liverpool, United Kingdom; 3 Staffin Museum, Staffin, Isle of Skye, Scotland, United Kingdom; Dakota State University, UNITED STATES OF AMERICA

## Abstract

Although globally scarce, Middle Jurassic dinosaur tracks are known from the Isle of Skye, Scotland, and help indicate the palaeoenvironmental preferences and behaviour of major dinosaur clades. Here, we report an extensive new tracksite from Skye: 131 *in-situ* dinosaur tracks at Prince Charles’s Point on the Trotternish Peninsula. The tracks occur in multiple horizons of rippled sandstones of the Late Bathonian aged Kilmaluag Formation, part of the Great Estuarine Group, which formed in a locally, shallowly submerged lagoon margin. We assign these tracks to two morphotypes, further divided into four morphotype subgroups, most likely representing large megalosaurid theropods, and sauropods that are either non-neosauropods or basal neosauropods. The trackways, although relatively short, evidence time-averaged milling behaviour, as observed at other tracksites in the Great Estuarine Group. The presence of sequential manus and pes sauropod tracks amends their previous identification by geologists as fish resting burrows, raising the potential that other such structures locally and globally may in fact be dinosaur tracks, and emphasises the predominant occurrence of sauropods in lagoonal palaeoenvironments in the Great Estuarine Group. At Prince Charles’s Point, however, unlike previously described lagoonal assemblages, large theropod trackmakers are more abundant than sauropods.

## Introduction

The Middle Jurassic (c. 174–164 Ma) was a time when many dinosaur clades including large-bodied and long-necked Eusauropoda/Neosauropoda, herbivorous Ornithischia, and carnivorous Theropoda (specifically allosauroids and coelurosaurs) diversified [1–4]. Despite this apparent diversification, the Middle Jurassic fossil record is sparse [5], and much of our understanding of dinosaur evolution from this time is implied from dated phylogenies rather than directly supported by considerable fossil evidence.

On the Isle of Skye, the Great Estuarine Group (c. 170–166 Ma) is one of the most fossiliferous Middle Jurassic (Late Bajocian?-Bathonian) sedimentary sequences in the UK with a variety of subtropical terrestrial faunas [6–8]. Vertebrates from this sequence include crocodylomorphs, salamanders, stem lizards, turtles, docodont and tritylodontid cynodonts, and pterosaurs [7–20]. Although scarce, dinosaur body fossils are also present on Skye and a few other Inner Hebridean isles in Western Scotland. At present, specimens from Skye include: several sauropod teeth and vertebrae, a sauropod humerus, and a thyreophoran ulna and radius [21–25]. Theropods are represented by several teeth and a middle caudal vertebra [26–28]. Additionally, a thyreophoran limb bone, referred to a stegosaur fibula, was documented from the nearby Isle of Eigg [29].

Given the paucity of bone material, Scottish dinosaur communities have been primarily inferred from Skye’s abundant track record. The first scientifically described track (GLAHM V1980) by [30] was found in the Lealt Shale Formation at Rubha nam Bràthairean (Brothers’ Point) and is a large tridactyl track variably discussed as either a theropod [30] or an ornithopod [31–32]. Subsequent trackway discoveries throughout the Great Estuarine Group, including in the Lealt Shale, Duntulm, Valtos, and Kilmaluag formations, have illuminated a variety of dinosaurs present on Skye in the Middle Jurassic. These include theropods, sauropods, thyreophorans (particularly stegosaurs), and putative ornithopods [32–38]. Dinosaur tracks in the Kilmaluag Formation (c. 167 ma), in particular, include a small assemblage of theropod tracks from Lùb Score [35]. In general, however, tracks from the Kilmaluag Formation, largely formed in and near freshwater lagoons, are rarer than those preserved in the Great Estuarine Group facies formed on beaches, in or near brackish lagoons, or in fluviodeltaic settings.

**Fig 1:**
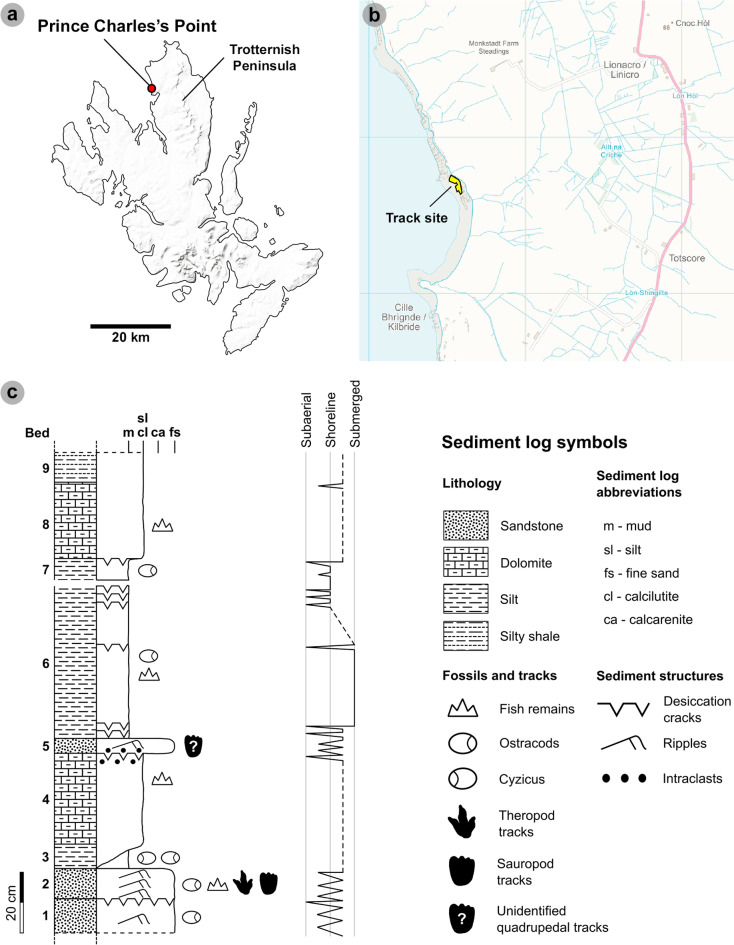
Geographical and geological context of the Prince Charles’s Point tracksite. The tracksite is situated on the northwest coast of the Trotternish peninsula. (A) Overall map of the Isle of Skye. MiniScale® [EPS geospatial data], Scale 1:1000000, Tiles: NG; NM, Updated: 20 November 2023, Ordnance Survey (GB), Using: EDINA Digimap Ordnance Survey Service (https://digimap.edina.ac.uk/os), Downloaded: April 2024. Contains public sector information licensed under the Open Government Licence v3.0. Available at: https://www.nationalarchives.gov.uk/doc/open-government-licence/version/3 (B) Overview map with the tracksite highlighted by a yellow polygon. OS VectorMap™ District [TIFF geospatial data], Scale 1:25000, Tiles: ng36_clipped, ng46_clipped, Updated: 23 September 2024, Ordnance Survey (GB), Using: EDINA Digimap Ordnance Survey Service (https://digimap.edina.ac.uk/os), Downloaded: December 2024. Contains public sector information licensed under the Open Government Licence v3.0. Available at: https://www.nationalarchives.gov.uk/doc/open-government-licence/version/3 (C) Stratigraphic log of the Kilmaluag Formation exposed at Prince Charles’s Point. Log is adapted from [40,48 ] to show new observations – including beds with dinosaur tracks.

In this study, we document 131 newly discovered *in-situ* dinosaur tracks in the Kilmaluag Formation at Prince Charles’s Point, situated on the northwest coast of Skye’s Trotternish Peninsula (Fig 1). This site includes extensive and morphologically well-defined trackways of large theropods and sauropods, some of which are among the longest continuous track sequences yet found on Skye. Interestingly, some of the sauropod tracks encompassed features that had previously been identified as ‘fish resting/burrow traces’ [39]. Here, we present an alternative argument to show that they match the morphology of sauropod tracks, which opens the possibility that other such structures in local and global stratigraphy may in fact be dinosaur tracks. The presence of sauropods amongst theropods provides further evidence for their preferential use of lagoonal palaeoenvironments on Skye. Such tracks furthermore complement other *in-situ* tracksites and body fossils found on Skye.

### Geological and stratigraphic context

The Great Estuarine Group contains a series of sedimentary formations deposited c. 170–166 Ma. The sediments represent alternating freshwater and marine-influenced delta and lagoonal systems in a paralic sequence [6,40,41]. Its constituent sedimentary formations crop out across the Inner Hebrides and were deposited across two basins – the Inner Hebrides Basin and the Sea of the Hebrides Basin. The Inner Hebrides Basin extended from the present-day Straithaird Peninsula, Skye, to the Isles of Eigg and Muck. The more northerly Sea of the Hebrides Basin extended from the Outer Hebrides landmass to the Central Skye Palaeo High – a hypothetical landmass now occupied by the Cullin Mountains [6,41]. Prince Charles’s Point would have been situated ~ 42.8*°*N within the Sea of the Hebrides basin [42]. The basin formed part of a series of low-lying small landmasses and shallow seas which resulted from localised volcanic induced uplift and falling global eustatic sea levels [41,43].

At Prince Charles’s Point, [40] logged a nine-bed sequence < 2 m in thickness (Fig 1C). The sequence is correlated to the clastic facies of the upper Kilmaluag Formation (late Bathonian, ~ 167 Ma). This correlation is supported by the presence of the freshwater ostracod *Darwinula* in most beds [40,41,44]. The sequence represents an ephemeral, low salinity, closed lagoonal system within a vast river delta alternating between subaerial and submerged states [40,45]. Beds 1-2 and 5 represent a regressive, lagoon margin (near-shoreline) palaeoenvironment, characterised by horizons of fine-grained rippled sandstones deposited by small rivers which fed the lagoon [40]. A lack of radial and desiccation cracks in and around dinosaur tracks present in bed 2 and 5 suggests the surface was shallowly submerged [46]. Intermittent periods of evaporation and extensive drying are indicated by brecciated and desiccated calcilutite dolomite and desiccated argillaceous shales (beds 4 and 8) [47]. Beds 6-7, and 9 are composed of argillaceous shales and silty shales and signify prolonged periods of increased river sediment input and lagoon margin transgression [40].

**Fig 2:**
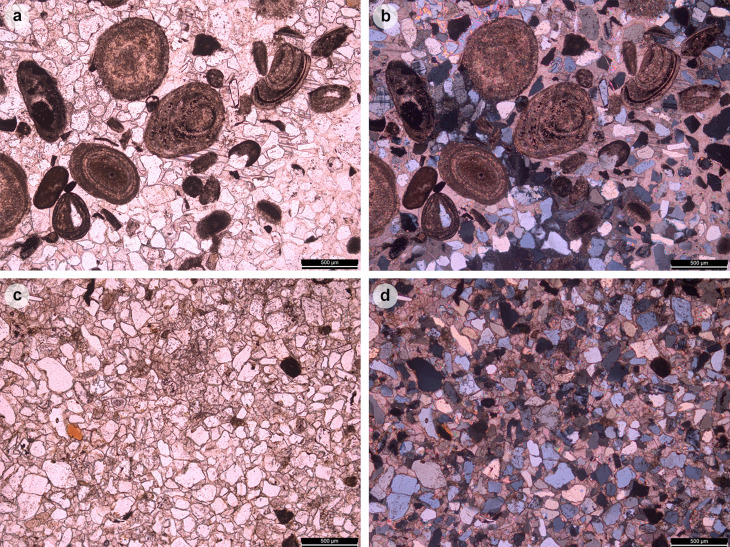
Sediment thin sections of track-bearing beds. Bed 2 (A, B) preserves abundant ostracods while Bed 5 (C, D) lacked ostracods. Sections were examined under plane polarised light (A, C) and cross polarised light (B, D).

At Prince Charles’s Point, the majority of dinosaur tracks occur within bed 2 – measured by [40] as a ~ 10 cm thick bed of fine-grained sandstone with abundant ostracods (Fig 2); we note here that the thickness of this bed varies laterally and can be greater than 10 cm. We determined a south westerly bed dip of ~ 4° in bed 2. Additional tracks occur in bed 5 – a ~ 5 cm thick bed with near identical sediment properties to bed 2 (Fig 2). Bed 5 differs with the presence of intraclasts and lack of ostracods [48]. Both beds are pale-dark grey in colour on fresh surfaces and weather orange or dark grey.

Bed 2 is composed of multiple generations of shallow, wave-generated, bifurcated, asymmetric current ripples [40]. Bed 5, in contrast, is composed of at least a single generation of these ripples. Each set of ripples typically varies between 2-4 cm in vertical thickness. Track-bearing horizons in beds 2 and 5 typically feature ripple wavelengths between ~ 5-8 cm. Non-track-bearing horizons in the same beds are characterised by ripple wavelengths between ~ 9-17 cm. Such wavelengths imply a greater water depth which might indicate inaccessibility to dinosaurs [49]. In a closed lagoon palaeoenvironment, wave-formed ripples may have formed perpendicular to wind-induced currents over very shallow water [49].

### Sequence biota

Other than dinosaur tracks, the outcrop at Prince Charles’s Point records a relatively minimal floral and faunal assemblage. Fish fragments are present in all beds, including a poorly preserved jawbone from bed 4 and scales within beds 2 and 8. Ostracods were previously recorded in infilled desiccation cracks within beds 6-9, and some exhibited rare ‘cup-in-cup’ preservation [48]. We additionally recorded ostracods in beds 1-2 (e.g., Fig 2A-B). A palynological study by [50] highlighted the presence of terrestrial miospores, predominantly gymnospermous pollen. Pollen such as *Lycopodiacidites baculatus* within the Prince Charles’s Point sequence constrained the locality to no younger than the Bathonian [51]. The presence of the planktonic alga *Botryococcus* and lack of marine palynomorphs provided further evidence that the depositional palaeoenvironment was non-marine [50]. The predominant absence of invertebrate bioturbation in all exposed beds may have reflected the rate of sediment accretion, food availability, or variation in salinity as recorded in deltaic palaeoenvironments [52–53].

### Tracksite discovery

In the early 1980s, Prince Charles’s Point was visited by Julian Andrews, who measured the section and documented ‘circular obstacle marks’ in bed 2 [40]. Later, Andrews discussed the possibility that the structures were ‘animal tracks’ but proceeded to identify them as fish resting burrows [39] – see ‘interpretation of sauropod tracks’ discussion section.

In August 2019, Tone Blakesley (TB), Dugald Ross (DR), Anne Martin (AM), and Victoria Bradder (VB) visited Prince Charles’s Point and found three tridactyl tracks on the northerly, bed 2-dominated wave-cut platforms. The vast majority of tracks and trackways were discovered during subsequent fieldwork led by TB in July-August of 2021 and 2022 and in April 2023 and 2024. TB described this tracksite for his MScR dissertation at the University of Edinburgh [54].

## Materials and methods

### Sampling permissions and ethics statement

Permission to conduct fieldwork, unmanned aerial vehicle (UAV) based photography, and collect sample sediments was provided by Ewan MacPherson (Kilmuir Estate landowner). No organic material was collected for this study.

### Field recording

Prince Charles’s Point is a tidal platform and can only be accessed at low tide (ideally with a high tidal coefficient). Prior to the morphological description, photography, and measurement of *in-situ* tracks, seaweed and limpets were carefully removed from in and around tracks while seawater was sponged out of concave epirelief tracks. Trackways are regarded as sequences with ≥ 3 tracks, while track associations featured two consecutive tracks, *sensu* [55].

Each track is catalogued with a site-specific number with prefixes to denote: the locality (‘PC’ for Prince Charles’s Point); clade designation (‘TH’ for theropods, ‘SA’ for sauropods, ‘U’ for unidentified); ‘A’ for associated tracks or ‘I’ for isolated individual tracks; trackway/track association number; and track number. ‘PC-TH-1-07’ for example is theropod trackway one and track seven at the locality. During fieldwork, we described each track and recorded their location, bed, symmetry, relief, and preservation grade (see S1 Appendix of S7 Table). Orientations for isolated tracks were recorded in the field with a compass.

### Morphological preservation grading

Previous studies by [56–57] graded the ‘morphological preservation’ of tracks on an ordinal scale of 0-3 (at 0.5 intervals) to state how well preserved a track is. Tracks graded closer to 3 are considered more diagnostic and suitable for characterising morphotypes. Grades are informed by the presence or absence, and sharpness and wear, of morphology. For our theropod tracks, grades are influenced by the preservation of digits ii-iv (digit i if present) and, for each digit, ungual marks, tapering, and phalangeal and metatarsophalangeal pads. Our sauropod tracks grades are influenced by the preservation of an overall track shape, digits i-iv, ungual marks, heel and pedal pads, and pad creases. The grading criteria are outlined in Table 1 in [57].

Complete theropod tracks graded ≥ 1.5 are measured and used to characterise morphotypes. Theropod tracks graded 1 are referred to specific morphotypes. Theropod tracks graded < 1 are either incomplete or possess poorly defined margins and are not measured or used to characterise morphotypes. Sauropod tracks graded ≥ 0.5 are used to characterise morphotypes.

### Photogrammetric datasets of tracks

Photogrammetric image sets for individual tracks consist of ≥ 50 photographs taken at three relative angles in a circular rotation on a 24.2-megapixel resolution Nikon D5600 with a 35mm lens. Specific trackways were photographed with the camera attached to an ~ 2 m high monopod with a shutter release controller. Photographs were taken at near nadir angles and sequentially overlap. Each track or trackway was photographed in calm weather in bright, diffuse lighting conditions. Camera parameters included: aperture ≥ f13, ISO ≤ 640, and shutter speed ≥ 1/200. These parameters ensured that the photographs were sharp and not too grainy.

We used a DJI Matrice 300 RTK UAV with a payload of a 45-megapixel Zenmuse P1 camera to survey the entire tracksite. All UAV flights were conducted in calm weather conditions with wind speeds < 15 mph. Due to weather constraints and UAV availability, flights took place in sunny conditions with the exposure compensation (EV) value lowered (up to -1.0) on photographs to compensate for bright lighting. The UAV was piloted via the DJI Pilot 2 app installed on a smart controller and flown autonomously using the smart oblique mode setting. This enabled the collection of a total of 4,528 images at five angles (928 images were nadir) at a 1.5-3 mm per pixel resolution from 12 m above the surface.

Prior to flights, corner markers were placed to establish the limits of a defined ‘polygon’ area of interest. Ground control points (GCPs) were then placed randomly across the tracksite. Coordinates (WGS84) of the GCPs were recorded using Emlid Reach RS survey units. The base station was set up outside the area of interest at set coordinates. The receiver units were operated through the Emlid Flow iPhone app. The coordinates were post-processed through Emlid Studio using RINEX data from the Ordnance Survey Benbecula Continuously Operating Reference Station (CORS) for an absolute positioning reference. The reference station is 60.1 km from the tracksite.

### Generation of photogrammetric models

Track and trackway 3D models were generated in Agisoft Metashape (Version 1.8.0, 64 bit, 2023) installed on two workstations (Athena and Goliath) based in the University of Edinburgh Airborne Research and Innovation facility. Workstation specs are: Athena (i7-7820X 8-core (16 with hyper-threading) and 1 x NVIDIA GeForce GTX 1080 Ti 11GB, 128GB RAM) and Goliath (2 x Intel Xeon E5-2640V4/ 2.4 GHz processor Total: 20 cores, 40 threads, and 2 x ASUS GTX1080TI-FE, 256GB RAM). Models were generated following the workflow from [32]. The resolution parameters for point cloud and mesh generation were set to the ‘highest’ setting. For sharp texture generation, the source data was set to ‘3D model’ and other fields left on default. Due to the large area of the tracksite, high resolution models were generated from UAV imagery of two sections. Each section contained a concentration of several trackways. A model of the entire tracksite meanwhile was generated at lower resolution parameters (highest image alignment, high dense point cloud, medium mesh), to ensure the tracksite model was manipulable in post-processing software. This combination of resolutions ensured ripple marks were also resolvable.

For handheld imagery of individual tracks or trackways, check points were placed to denote a scale reference on the track-bearing surface after the medium imagery alignment process. The scale calibration was then used to optimise the imagery in place of the GCPs. These were generated following the same ‘highest’ resolution workflow.

### Post-processing photogrammetric models

Photogrammetric models were post-processed in Meshlab (Version 2022.02, 64 bit). Models > 2 GB were processed on a University of Edinburgh Geosciences CT lab workstation PC (Intel Xeon W-2125 CPU at 4.00GHz with NVIDIA GeForce GTX 1060 6GB, 160GB RAM). A laptop workstation processed models < 2 GB (i7-11800H at 2.3 GHz with NVIDIA RTX™️ A3000 (6 GB GDDR6 dedicated) 32GB RAM). Meshlab was used to remove excessive mesh and boulders on trackway models to accurately set planes through the track-bearing surface. Subsequently generated digital representations included textured orthophotos, digital elevation maps (DEM), and contour maps in Paraview (Version 5.10.1) also installed on the laptop workstation. Contours on contour maps are spaced by 2 mm. Model outlines were drawn in Inkscape (Version 1.2.2, 64 bit).

### Track measurements

**Fig 3:**
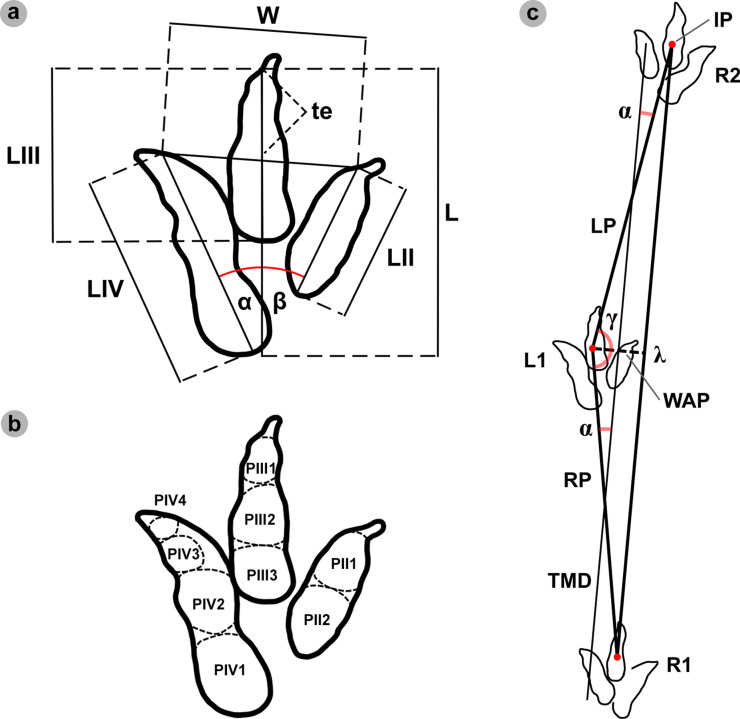
Theropod track and trackway measurements and track phalangeal pads. (A) Measurements for theropod tracks included: overall length (L), width (W), digit lengths (LII-LIV), toe extension length (te), and interdigital angles between digits ii-iii (α) and digits iii-iv (β). (B) Theropod track phalangeal pad configuration (2:3:4 formula) between digits ii-iv (right to left). (C) Bipedal trackway measurements derive from [55] and included: pace – the distance between common reference points (intersecting point (IP) of track length and width lines) on a sequential right and left track (RP and LP respectively); stride (λ) – the distance between two successive right or left track IPs; width of the pes angulation pattern (WAP) – overall width of stride from the IP of a respective alternate track; angle of rotation (α) – measured between the pace line and trackway midline (TML); and pace angulation (γ) – the angle of two intersecting pace lines from a preceding and proceeding track in the stride. The TML was drawn from the midpoints of pace lengths.

For theropod tracks, we measured: the overall length (L), width (W), digit lengths (LII-LIV), and toe extension of digit iii (te), as used in [55,58,59], in FIJI ImageJ [60] (Fig 3A). Full pes track length measurements enabled tracks to be classified in accordance with [58] (Table 1).

**Fig 4:**
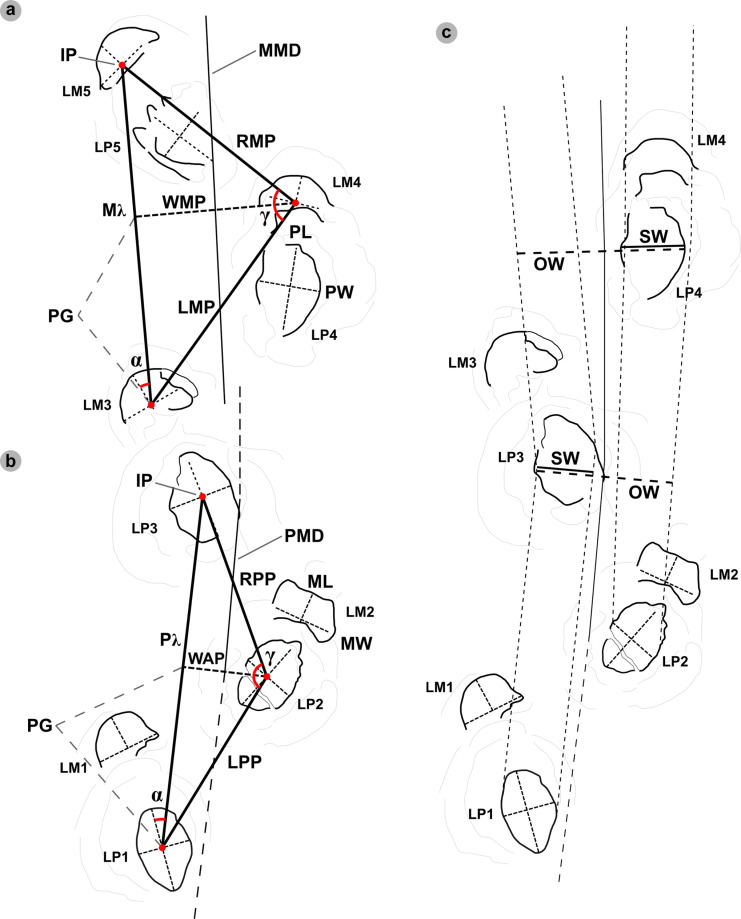
Sauropod track and trackway measurements. Measurements are based on [55,58]. (A-B) Pes and manus trackway measurements included overall pes (P) or manus (M) length (PL or ML) and width (PW or MW). As per bipedal tracks, right and left pes or manus paces (RPP or RMP and LPP or LMP) were measured between alternating track IPs, while strides (Pλ or Mλ) from the IP of two consecutive right or left tracks. Further measurements included the width of the pes angulation pattern (WAP), width of the manus angulation pattern (WAM), and the pes and manus angles of rotation (α) and pace angulation (γ). A track midline (PMD for pes and MMD for manus trackways) was drawn from the midpoint of pace lengths. Progression (PG) was based on the Pythagorean theorem and determined the distance between an IP of one track and WAP intersection point of a consecutive track within a respective stride. (C) The pes trackway ratio (gauge) was based on measurements perpendicular to the TMD including: side width ‘SW’ – measured across an individual track; and overall width ‘OW’ – measured across the outermost side width lines of consecutive pes tracks – as stipulated by [64].

For sauropod manus and pes tracks, the overall length and width were respectively measured across the long and short axes *sensu* [63] (Fig 4). Each track was classified by track length size *sensu* [58] (Table 1). Due to their poor definition across all tracks, digits and pads were described qualitatively, but not individually measured.

**Table 1:** Pes tracks are categorised by their length in accordance with [58].

Trackmaker	Track size classification	Pes length (PL)
**Theropod**	Tiny	<10 cm
	Small	10 cm ≤ PL < 20 cm
	Medium	20 cm ≤ PL < 30 cm
	Large	30 cm ≥ PL < 50 cm
	Giant	PL ≥ 50 cm
**Sauropod**	Tiny	PL < 25 cm
	Small	25 cm ≤ PL < 50 cm
	Medium	50 cm ≤ PL < 75 cm
	Large	PL ≥ 75 cm

Length/width (l/w) ratios illustrated whether a track was longer than wide or vice versa. Track width and te determined the track mesaxony (te/W) – i.e., how elongated, well-developed digit iii was *sensu* [59]. Digit lengths were measured in accordance with [55], from the base of the digit to its most distal point (excludes ungual marks). These lengths were used to calculate digit ‘iii/ii’ and ‘iii/iv’ ratios *sensu* [61]. Phalangeal pads were identified in accordance with [59] (Fig 3B). Interdigital angles between digits ii-iii (α) and digits iii-iv (β) were measured following [62] in Inkscape. The sum of these interdigital angles provided a digit ii-iv divarication angle.

### Trackway measurements

We measured trackway characteristics of both theropod (Fig 3C) and sauropod (Fig 4) trackways based on [55,58] using FIJI ImageJ [60] and Inkscape. Stride (λ) was measured between the intersecting point (IP) of the overall track length and width lines of two consecutive tracks impressed by the same foot. The right or left pace (RP or LP respectively) were measured between the IP of two consecutive, alternating tracks. The width of pes angulation (WAP) intersects the stride length line perpendicular from an IP and measures the trackway width. The track midline (TMD) was drawn between the midpoint of the pace length lines and interpolated for the first and final tracks in a visible sequence. The pace angulation (γ) was measured based on the intersection of two pace lines at an IP. The angle of rotation (α) was measured from the intersection of the pace length and TMD lines between two alternating tracks in the forward direction of the trackway. Track orientations for sauropod pes and manus tracks were measured between respective forward stride lines and track long axes (Fig 4A-B).

The approximate track area of sauropod pes tracks (IPS) was calculated using Eq 1, while Eq 2 determined the manus track area (IMS) [65]. Heteropody (Eq 3) expressed the difference between the areas of a paired pes and manus track as a ratio [66]. While [67] illustrates carpal width might provide a better overall estimate, an absence of clearly defined digits on most of the sauropod tracks at Prince Charles’s Point precluded that approach to assessing the heteropody ratio. Heteropody was not calculated when manus tracks were overprinted or absent.


$$IPS={\left(\text{Pes length x Pes width}\right)}^{0.5}$$ (1)

$$IMS={\left(\text{Manus length x Manus width}\right)}^{0.5}$$ (2)

$$Heteropodyratio=\frac{IPS}{IMS}$$ (3)

Sauropod pes trackway gauges were classified based on trackway ratios (TR) as proposed by [64] (Eq 4) – in which side width (SW) was measured across an individual track, while overall width (OW) was measured between the outermost side width lines of respective pes tracks and perpendicular to the TMD (Fig 4C). The equation output a percentage value based on measurements taken perpendicular to the track midline for pes and manus tracks. The equation expanded on previous gauge designations by [68] to provide values that signify: wide below 35%, medium between 35-50% and narrow above 50%.


$$TR=\frac{\text{SW}}{\text{OW}}\text{x }100$$ (4)

Trackway gauge was further quantified through dividing WAP by pes length (PL) (Eq 5) or the width of manus angulation (WAM) by manus width (MW) (Eq 6) [58]. Pes gauge ratios could then be assessed as: narrow below 1.0, medium between 1.0-1.2, and wide above 1.2. Eq 5 also determined the extent to which pes tracks touched the TMD: overlaps midline below 1.0, and separated from midline above 1.0. The gauge difference and proximity of manus tracks to pes tracks to the track midline was determined by a WAP/WAM ratio (Eq 7) – signified as: further than pes below 1.0, and nearer than pes above 1.0.


$$Pesgaugeratio=\frac{\text{WAP}}{\text{PL}}$$ (5)

$$Manusgaugeratio=\frac{\text{WAM}}{\text{MW}}$$ (6)

$$Manusmidlinedistanceratio=\frac{\text{WAP}}{\text{WAM}}$$ (7)

Gauge was also assessed according to [58] from ratios of WAP with the index of pes size (IPS) and/or WAM with the index of manus size (IMS) (Eqs 8-9).


$$Pesindexgaugeratio=\frac{\text{WAP}}{\text{IPS}}$$ (8)

$$Manusindexgaugeratio=\frac{\text{WAM}}{\text{IMS}}$$ (9)

Due to the low number of sequential tracks per quadrupedal trackway, progression was used to determine the forward motion of a trackmaker in a single footfall for pes and manus tracks (Eqs 10-11).


$$Pesprogression=\sqrt{Pace^{2}-WAP^{2}}$$ (10)

$$Manusprogression=\sqrt{Pace^{2}-WAM^{2}}$$ (11)

An estimated trackmaker hip height (h) was obtained by multiplying a track length (FL) by four in Eq 12 [69]. Hip height further informed velocity calculations. Velocity (V) was calculated per stride (three consecutive tracks as per [55]) (Eq 13). Average values of track length and stride across a trackway were used to calculate overall velocity and enabled comparison between trackways. The calculation utilised a trackway average stride length (λ), acceleration due to gravity (g =  9.81 m/s^2) and average estimated hip height (h) (based on the mean FL per λ) [69]. An updated version of this equation, devised by [70], is also used (Eq 14). Stride length was furthermore divided by an estimated hip height to estimate trackmaker gait (Eq 15) *sensu* [71]. Gait values were approximated as: walking below 2.0, trotting between 2.0-2.9, and running above 2.9.


h=4FL (12)

$$V=0.25g^{0.5}{\text{λ}}^{1.67}h^{-1.17}$$ (13)

$$V=0.226g^{0.5}{\text{λ}}^{1.67}h^{-1.17}$$ (14)

$$Gait=\frac{\text{λ}}{h}$$ (15)

### Morphotype classification

**Fig 5:**
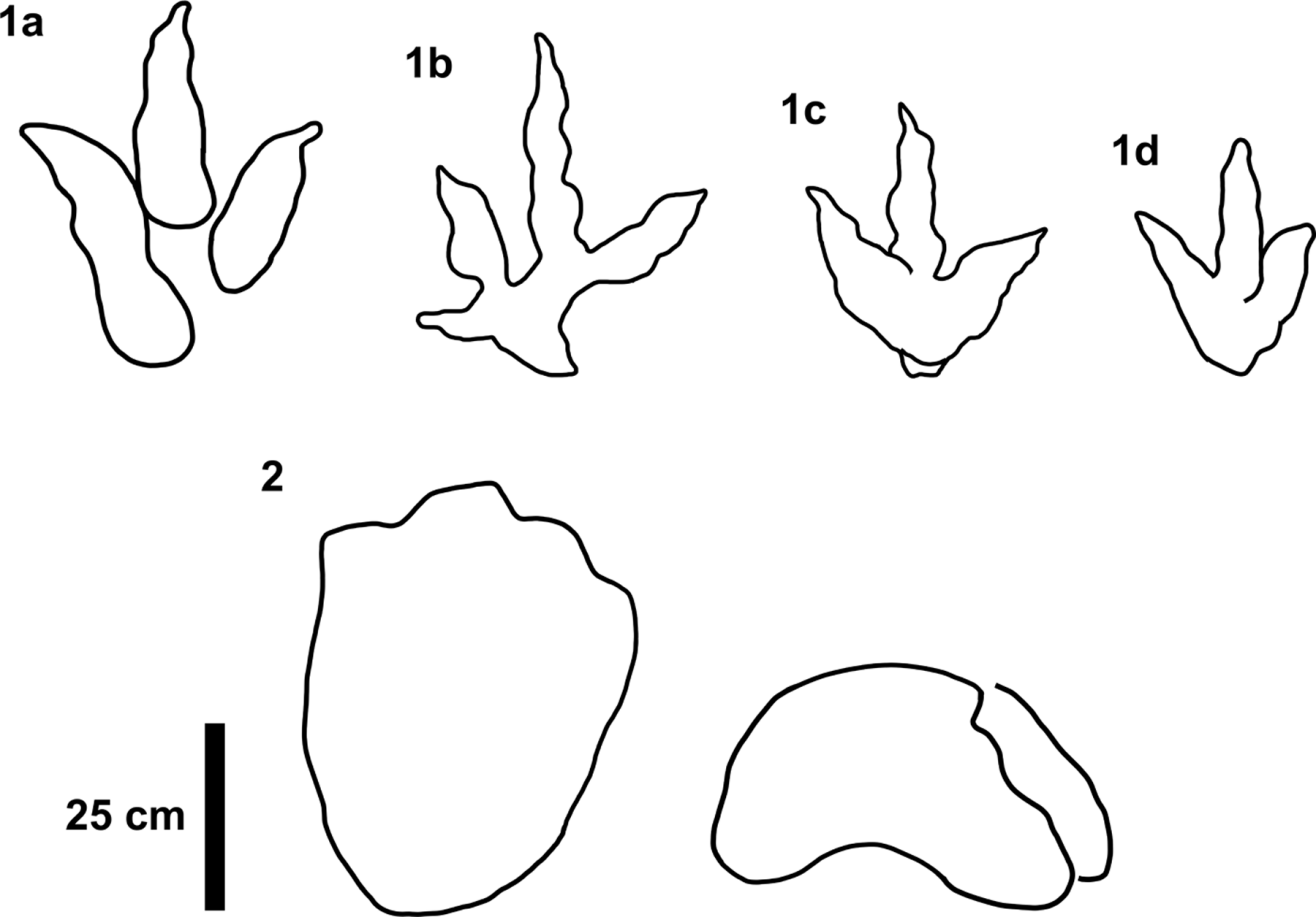
Track morphotypes recorded at Prince Charles’s Point. Note outlines for morphotypes 1b and 2 are right pes tracks, the rest are left.

Both track metrics and morphology were used to group tracks into tracksite morphotypes (Fig 5). Tridactyl morphotypes were further subgrouped based on subtle differences and shared morphologies or metrics. Morphotype subgroups featured an additional letter added onto the end of each number. The main representative morphotype of the group was first, e.g., morphotype-1a. Each morphotype was compared with previously described ichnotaxa with similar metrics and morphologies to suggest a representative ichnotaxon and inform on possible trackmakers.

### Trackmaker orientations and palaeocurrent directions

The orientations of tracks and flow directions (ripples) were digitally measured from the tracksite DEM. Track orientation categories included trackways, track associations, and isolated theropod tracks. Trackways and track associations were measured based on a midline. Isolated theropod track orientations were measured in the field from the length line. Flow directions were measured perpendicular to ripples around tracks (if visible). The steeper lee side of ripples represent forward flow directions [49].

## Results

### Tracksite summary

**Fig 6:**
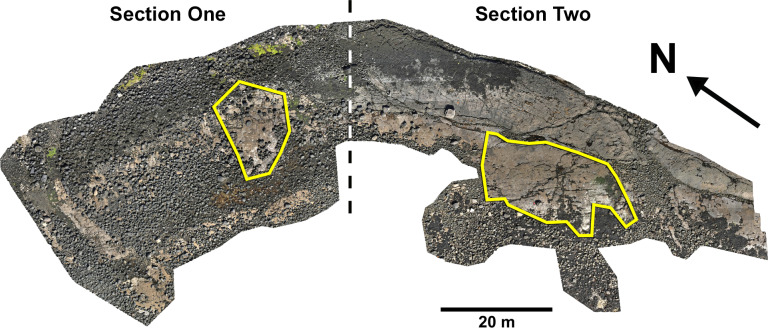
Overview orthomosaic of Prince Charles’s Point. UAV orthomosaic of full tracksite with Section One and Section Two divided accordingly by a dashed line. Track clusters in each section are highlighted in each by a yellow polygon.

A total of 131 tracks were catalogued and preservation graded (65 theropods, 58 sauropods, 8 unidentified). Out of these tracks, 77 were measured, with 16 theropod and 27 sauropod tracks used to classify morphotypes (Fig 5). The tracks generally occur within or near two clusters, each in their own ‘section’ (Fig 6).

**Fig 7:**
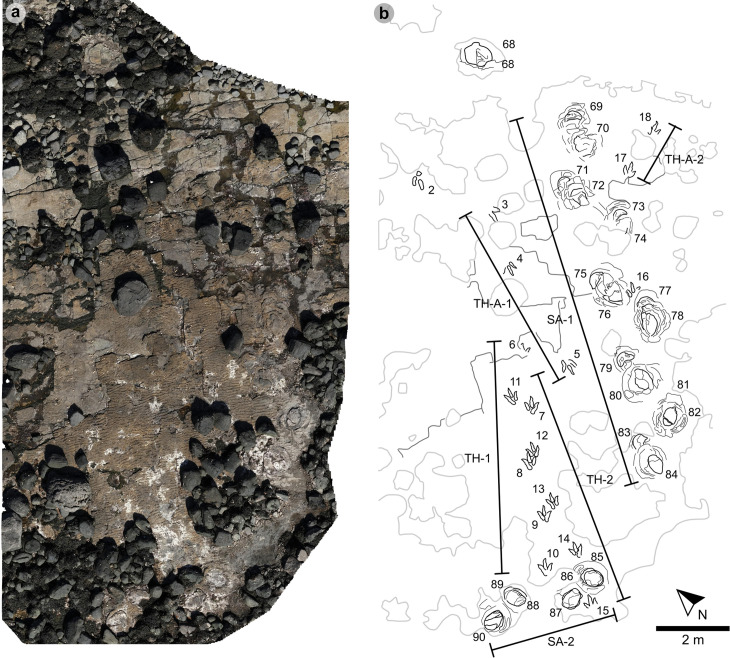
UAV orthophoto and outline map of main track cluster in Section One. (A) In the orthophoto, the rippled sandstones of bed 2 gradually wear down toward the worn surface of bed 1 exposed toward the present-day high-tide mark due to a ~ 4° south-westerly dip. The orthophoto was composited from photographs taken by our UAV during fieldwork in April 2023. (B) The outline map highlights the position and distribution of tracks. Each track is labelled with its tracksite catalogue number. Trackways of ‘TH’ represent theropods, ‘SA’ signify sauropod, and ‘TH-A’ denote theropod track associations. Dark grey outlines highlight the bedding plane edge. Pale grey outlines signify boulders.

In Section One, most tracks occur in a single horizon of bed 2 exposed as a partially boulder-covered, intertidal platform. A cluster of 17 (predominantly concave epirelief) theropod and 23 (shallow convex epirelief) sauropod tracks occur in a ~ 92 m^2^ clearing – as shown in our tracksite orthophoto (Fig 7). This includes morphotype-1a theropod track sequences ‘PC-TH-1 and 2’, associations ‘PC-TH-A-1 and 2’, and morphotype-2 sauropod trackways ‘PC-SA-1 and 2’. A trackway which includes an example of morphotype-1b (PC-TH-3) is situated 20.2 m north of the main track cluster and distantly surrounded by further isolated morphotype-1a and morphotype-2 tracks. Tracks impressed by an undetermined quadrupedal trackmaker, i.e., PC-U-A-1-66 to 67, PC-U-A-2-93 to 95, PC-U-A-3-96 to 97, and PC-U-A-4-98 to 100, were recorded in bed 5 below the ~ 92 m^2^ track accumulation. As most tracks in bed 5 have suffered considerable wear, they are not measured, figured or further described in this study.

**Fig 8:**
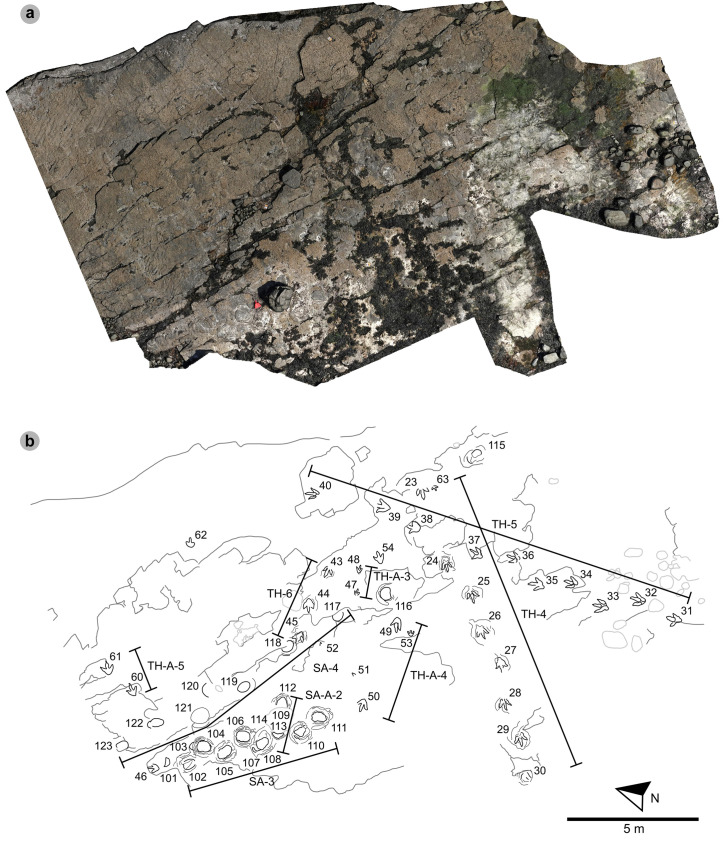
UAV orthophoto and outline map of main track cluster in Section Two. (A) In the orthophoto, extensive bed wear has revealed a variety of ripple wavelengths and bearings across multiple bed 2 horizons. The orthophoto was composited from photographs taken by our UAV during fieldwork in April 2023. (B) Outline map highlights the position and distribution of trackways. Each track is labelled with its tracksite catalogue number. Trackways of ‘TH’ represent theropods, ‘SA’ signify sauropod, and ‘TH-A’ or ‘SA-A’ denote track associations. Dark grey outlines highlight the edge of a bedding plane. Pale grey outlines signify boulders.

In Section Two, tracks occur across multiple scoured bed 2 rippled sandstone horizons and are worn by erosion. A cluster of 34 (mostly concave epirelief) theropod and 23 (shallow convex epirelief) sauropod tracks are present on a ~ 171 m^2^ area of intertidal platform – as shown in our tracksite orthophoto (Fig 8). These include morphotype-1a trackways ‘PC-TH-4 to 6’ and track associations ‘PC-TH-A-4 and 5’, amongst several isolated tracks. Morphotype-1d is solely represented by ‘PC-TH-A-3’. Morphotype-2 is most clearly represented by trackway ‘PC-SA-3’, alongside the worn and partial ‘PC-SA-4’ track sequence. Elsewhere, morphotype-1b is known from an isolated track (PC-TH-I-55) toward the northern end of Section Two. Morphotype-1c, represented by PC-TH-I-56 and 59, is present in the lower most bed 2 horizons to the south of the main Section Two cluster. Meanwhile, ‘PC-SA-5’, an isolated morphotype-2 trackway, represented at the northern end of the section is surrounded by sparsely distributed isolated morphotype-1a and 2 tracks.

### Morphotype-1a

**Fig 9:**
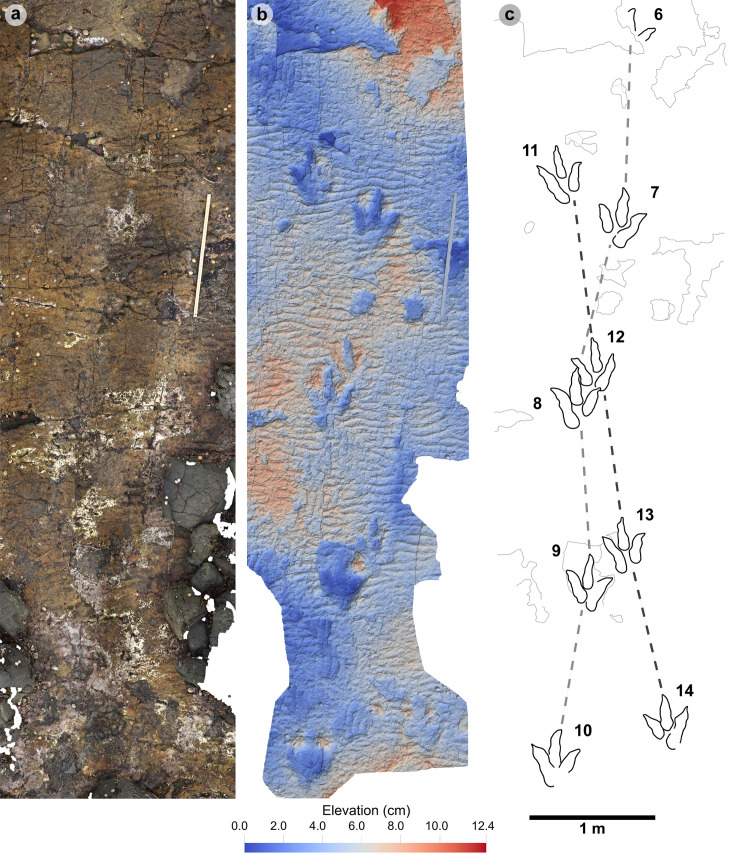
Overview of PC-TH-1 and 2. (A) Orthophoto, (B) DEM, (C) outline with trackways highlighted by dashed lines – PC-TH-1 is pale grey, while PC-TH-2 is dark grey. Both trackways are oriented in the opposite direction to the palaeocurrent inferred from ripples. A horizon which directly underlies the track-bearing surface features ripples oriented in a southeasterly direction, from which we infer variation in the current direction across relatively short timescales. Note PC-TH-2-15 is excluded from imagery as the track was most clearly exposed after the photogrammetric dataset was collected.

**Fig 10:**
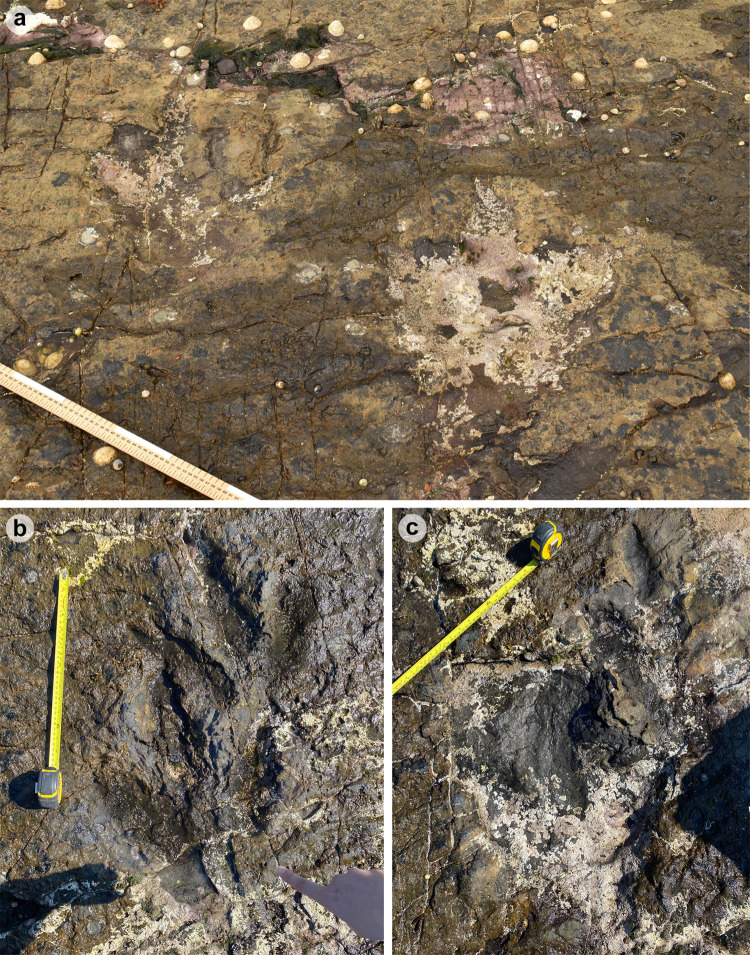
Photographs of selected PC-TH-1 and 2 tracks. (A) PC-TH-1-07 (right) and PC-TH-2-11 (left). (B) The distal regions of digit iii and ii on PC-TH-1-08 (left) overprint the lower margins of digit ii and heel region of PC-TH-2-12 (right). (C) PC-TH-1-09 (left) penetrated the substrate more deeply but suffers from present-day erosion, while PC-TH-2-13 (right) features a shallower relief with greater sediment infill and ripples.

**Material** – PC-TH-I-01, PC-TH-1-07 – 09, PC-TH-2-11 – 14, PC-TH-5-40, PC-TH-A-4-49 (Figs 9–10,14A-D,15A-D,16I-L; Table 2).

**Table 2:** Morphotype 1a track measurements.

						Digit length (DL)			DL ratios		Divarication angles				
**Specimen**	**PG**	**L/ R**	**L**	**W**	**L/W**	**II**	**III**	**IV**	**III/II**	**III/IV**	**II-III**	**III-IV**	**II-IV**	**te**	**M**
PC-TH-I-**01**	2	R	46.34	38.12	1.22	25.71	28.90	36.18	1.12	0.80	18.01	34.43	52.44	14.43	0.38
PC-TH-1-**07**	2	R	45.50	32.90	1.38	20.90	25.10	32.70	1.20	0.77	20.70	31.41	52.11	14.90	0.45
PC-TH-1-**08**	1.5	L	45.20	31.90	1.42	21.70	24.90	30.80	1.15	0.81	23.89	27.16	51.05	16.40	0.51
PC-TH-1-**09**	1.5	R	43.00	31.70	1.36	22.00	25.30	30.10	1.15	0.84	27.05	26.16	53.21	15.20	0.48
PC-TH-2-**11**	1.5	L	47.70	31.10	1.53	21.00	25.40	36.30	1.21	0.70	20.96	23.08	44.04	13.00	0.42
PC-TH-2-**12**	1.5	R	46.60	28.00	1.66	19.40	26.20	34.10	1.35	0.77	26.82	18.70	45.52	14.60	0.52
PC-TH-2-**13**	1.5	L	43.10	29.60	1.46	20.20	25.30	34.40	1.25	0.74	31.38	24.37	55.75	10.60	0.36
PC-TH-2-**14**	1.5	R	45.60	32.20	1.42	20.20	25.40	35.80	1.26	0.71	32.15	27.21	59.36	11.80	0.37
PC-TH-5-**40**	1.5	R	46.30	36.20	1.28	22.90	32.60	31.00	1.42	1.05	16.76	36.96	53.72	18.20	0.50
PC-TH-A-5-**49**	1.5	L	53.20	34.40	1.55	24.40	32.40	33.90	1.33	0.96	5.92	38.19	44.11	17.30	0.50
***AVERAGE***	***1.6***		***46.25***	***32.61***	***1.43***	***21.84***	***27.15***	***33.53***	***1.24***	***0.81***	***22.36***	***28.77***	***51.13***	***14.64***	***0.45***

Morphotype-1a is characterised by large track lengths, weak-moderate l/w ratios and mesaxony, and < 60° total divarication angles. Preservation grades (PG) are scored based on track morphology. The ‘L/ R’ column states whether a track was printed by a left or right foot. Measurements include: overall length (L), overall width (W), length/width ratio (L/W), digit lengths (DL) for digits ii-iv, digit iii/ii and digit iii/iv ratios (III/II and III/IV respectively), digit divarication angles between digits ii-iii (II-III), digits iii-iv (III-IV), and digits ii-iv (II-IV), toe extension (te), and mesaxony (M). All lengths were measured in cm. Divarication angles were measured in degrees. All track measurements were rounded to two decimal places.

**Fig 11:**
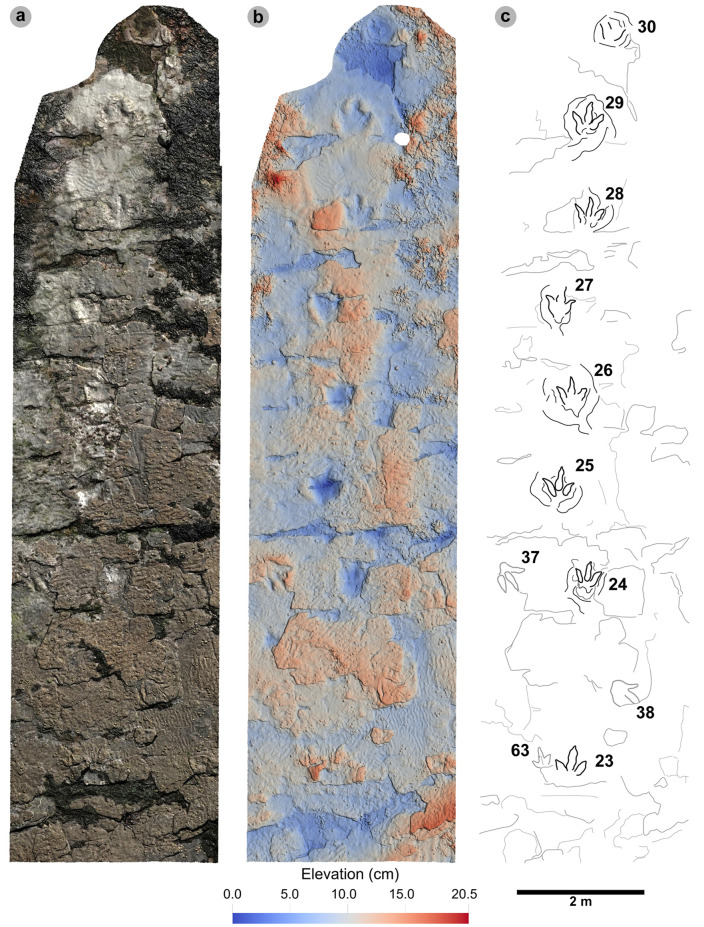
Overview of PC-TH-4. (A) Orthophoto with software-based shadowing, (B) DEM, (C) outline highlights the trackway. Note the greyed-out tracks of PC-TH-5 (37 and 38) can be seen crossing the path of PC-TH-4 in a northerly direction. The tracks of PC-TH-4 gradually become less well-defined through the sequence as present-day erosion exposed underlying layers.

**Fig 12:**
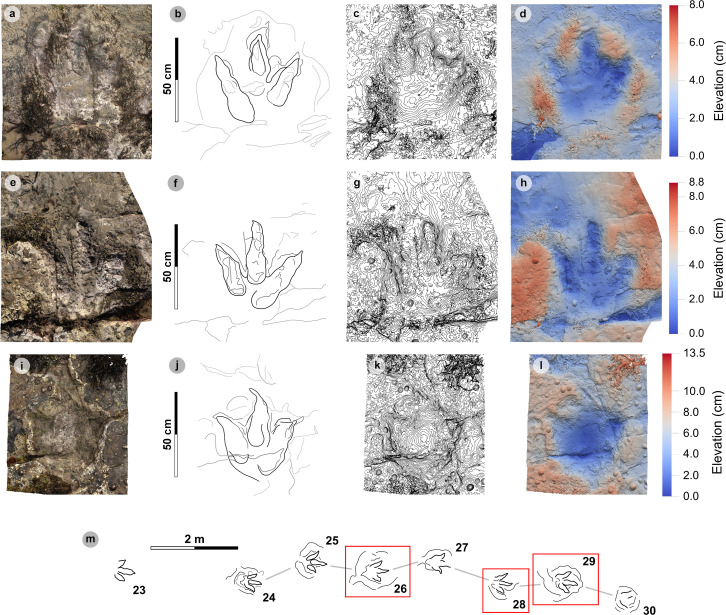
Digital representations of selected PC-TH-4 tracks. Textured orthophotos, outlines, contour maps, and DEMs are respectively represented for: (A-D) PC-TH-4-29, (F-H) PC-TH-4-28, and (I-L) PC-TH-4-26. The tracks likely represent worn surface tracks with morphotype-1a configurations (i.e., longer digit iv than ii) and low ~ 1.2 mesaxony. (M) Selected tracks, indicated by red boxes, in context to the rest of the trackway sequence. Due to present-day erosion, track margin definitions gradually decline across the sequence.

**Fig 13:**
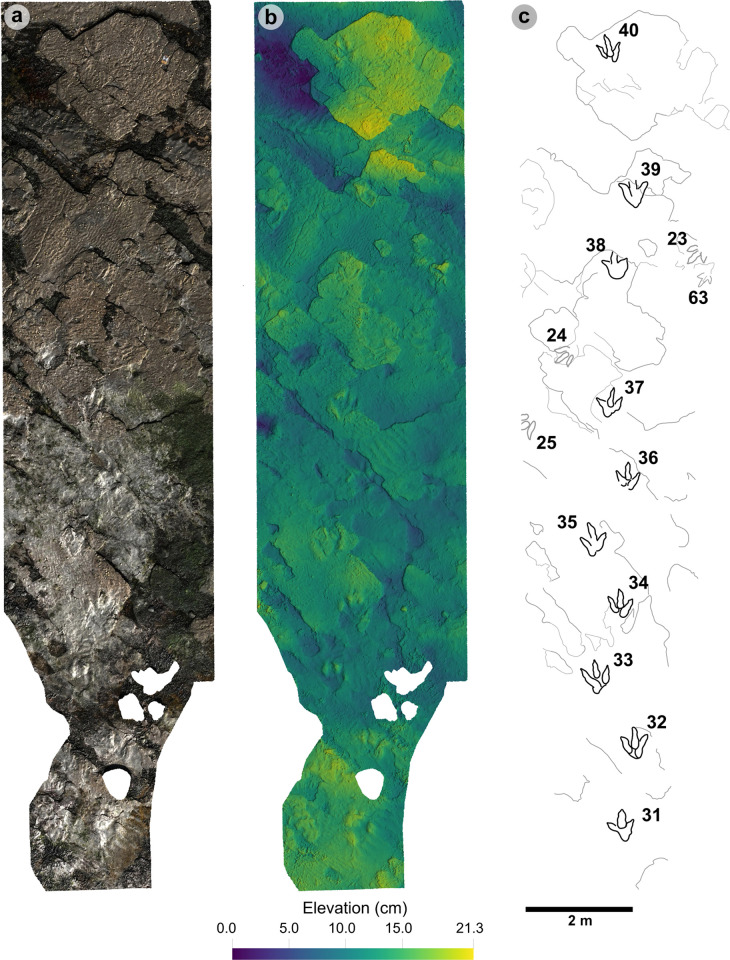
Overview of PC-TH-5. (A) Textured orthophoto with software-based shadowing, (B) DEM, (C) outline highlights the trackway. Note the greyed-out tracks of PC-TH-4 can be seen intersecting the south-westerly path of PC-TH-4. Although most tracks are eroded, some featured large divarication angles.

**Referred material** – PC-TH-1-06, 10, PC-TH-2-15, PC-TH-4-23 – 29, PC-TH-5-31-38, PC-TH-6-43 – 45, PC-TH-A-1-04, 05, PC-TH-A-2-18, PC-TH-A-4-50, PC-TH-A-5-60, 61, PC-TH-I-16, 41, 46, 54 (Figs 11–16; S1–S8 Figs).

#### Diagnosis.

Track lengths typically range between 40-55 cm. Some phalangeal pad margins more discernible than others based on digit margins and configured up to a ‘2:3:4’ formula. Digit iv features a large, sub-circular metatarsophalangeal pad. Digit ii-iv divarication angles are generally between 40°-60°. Digit i is absent.

#### Description.

A large, tridactyl footprint between 43.0-53.2 cm long and 28.0-38.1 cm wide (l/w ratio 1.22-1.66). Tracks are weakly-moderately mesaxonic (0.36-0.52) with broad, distally tapering digits. Digit margins are often parallel sided with anteriorly oriented, narrow, long ungual marks. Average digit iii/ii length ratios (1.24) are larger than digit iii/iv equivalents (0.81). Digits feature a clearly defined ‘2:3:4’ phalangeal pad formula (which include the shallowly impressed metatarsophalangeal pad on digit iv). Digit iv is the longest digit followed by digits iii and ii. Digit ii-iv divarication angles range between 44.0°-59.4° (average 51.1°).

### Morphotype-1a material description

#### PC-TH-1 and 2.

PC-TH-1 and 2 are the most diagnostic morphotype-1a trackways (Fig 9, Tables 3–8) and cross one another in Section One. Both exhibit similar trajectories with PC-TH-1 extending over 5.9 m at 47.5° relative to north and PC-TH-2 extending 6 m at 32.2° relative to north - against a south westerly flow direction implied by the ripples (219°). Each trackway is composed of five large tridactyl footprints. All except PC-TH-2-15 are in concave epirelief. PC-TH-1 tracks ranged in preservation grades between 0.5-2. PC-TH-2 preservation grades ranged between 1-1.5. Due to their incompleteness, PC-TH-1-06 and PC-TH-2-15 were not measured.

**Fig 14:**
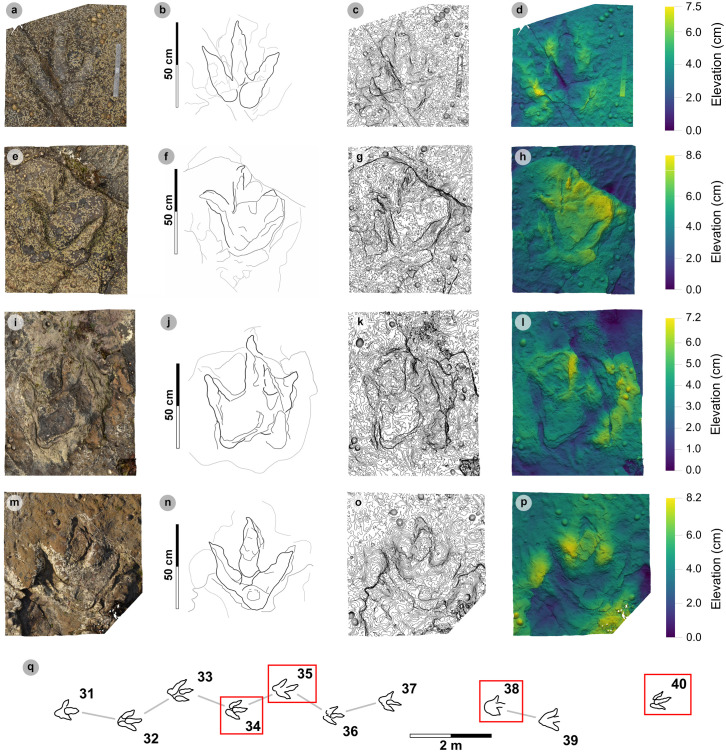
Digital representations of selected PC-TH-5 tracks. Textured orthophotos, outlines, contour maps, and DEMs are respectively represented from left to right. (A-D) PC-TH-5-40 enables other poorly preserved PC-TH-5 tracks to be referred as morphotype-1a. PC-TH-5 tracks are variably preserved: (E-H) much of PC-TH-5-38 is obscured by sediment, (I-L) PC-TH-5-35 exhibits wide digit ii-iv divarication which may have reflected the trackmaker turning, (M-P) PC-TH-5-34 was similarly widely divaricated. (Q) Selected tracks, indicated by red boxes, in context to the rest of the trackway.

**Fig 15:**
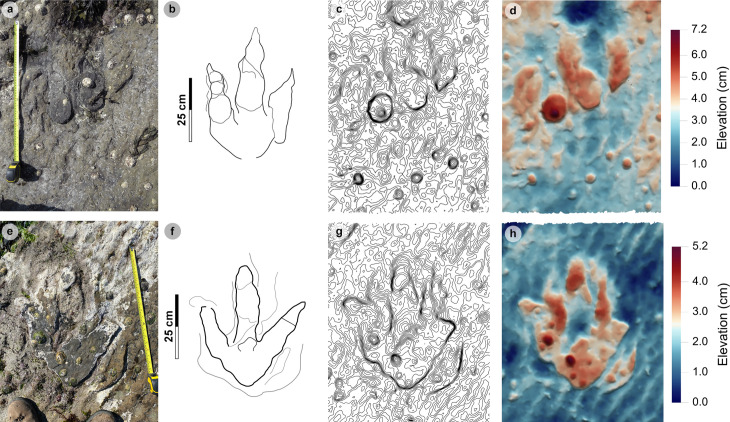
Overview of PC-TH-A-4 tracks. Photographs, outlines, contour maps, and DEMs are respectively represented from left to right. (A-D) PC-TH-A-4-49 was posteriorly heavily intruded by ripples and partially infilled on digits iii and iv. Unusually, the phalangeal pad creases for Piv3-1 are distinguishable. (E-H) PC-TH-4-50 retains most of its sediment infill and possesses a wider digit divarication angle than PC-TH-4-49.

**Fig 16:**
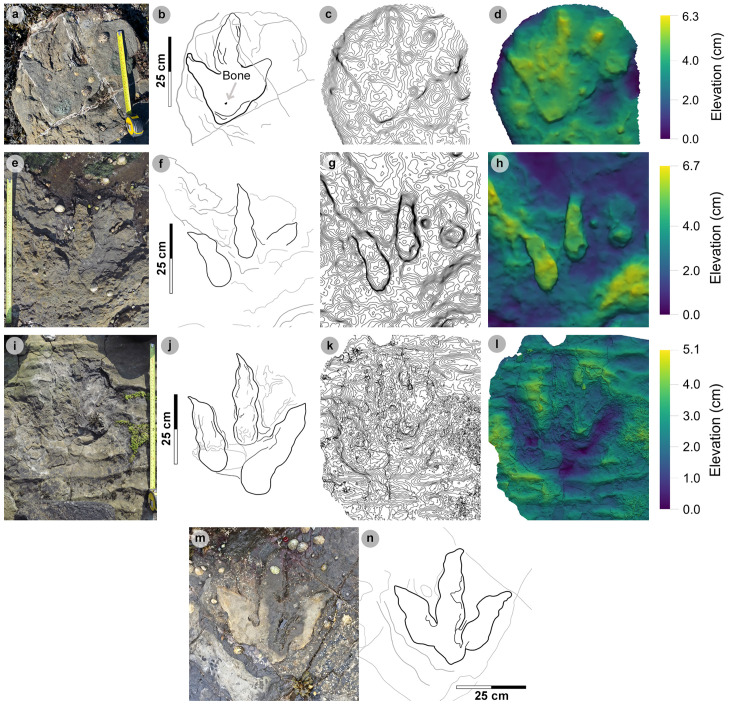
Photographic and digital representations of selected isolated tracks. Photographs, outlines, contour maps, and DEMs are respectively represented from left to right. (A-D) PC-TH-I-46, (E-H) PC-TH-I-16, (I-L) PC-TH-I-01, and PC-TH-I-41 (M-N). PC-TH-I-46 and PC-TH-I-16 occur in close proximity to sauropod trackways and were likely impressed in the same horizon. Unlike these tracks, PC-TH-I-01 was used to define morphotype-1a due to its clear digit margins, ‘2:3:4’ phalangeal pad formula, and sharp, elongated ungual marks. The track heel region is intruded by ripples. Due to a large boulder covering the anterior portion of the track, a photogrammetric model could not be produced for PC-TH-I-41.

**Table 3:** PC-TH-1 track measurements.

						Digit length (DL)			DL ratios		Divarication angles				
**Specimen**	**PG**	**L/ R**	**L**	**W**	**L/W**	**II**	**III**	**IV**	**III/II**	**III/IV**	**II-III**	**III-IV**	**II-IV**	**te**	**M**
PC-TH-1-**06**	0.5	L	n/a	n/a	n/a	n/a	n/a	n/a	n/a	n/a	n/a	n/a	n/a	n/a	n/a
PC-TH-1-**07**	2	R	45.50	32.90	1.38	20.90	25.10	32.70	1.20	0.77	20.70	31.41	52.11	14.90	0.45
PC-TH-1-**08**	1.5	L	45.20	31.90	1.42	21.70	24.90	30.80	1.15	0.81	23.89	27.16	51.05	16.40	0.51
PC-TH-1-**09**	1.5	R	43.00	31.70	1.36	22.00	25.30	30.10	1.15	0.84	27.05	26.16	53.21	15.20	0.48
PC-TH-1-**10**	1	L	45.80	32.50	1.41	18.60	25.40	31.50	1.37	0.81	36.69	27.33	64.02	17.10	0.53
***AVERAGE***	***1.3***		***44.88***	***32.25***	***1.39***	***20.80***	***25.18***	***31.28***	***1.22***	***0.81***	***27.08***	***28.02***	***55.10***	***15.90***	***0.49***

All lengths were measured in cm. Divarication angles were measured in degrees. All track measurements were rounded to two decimal places.

**Table 4:** PC-TH-2 track measurements.

						Digit length (DL)			DL ratios		Divarication angles				
**Specimen**	**PG**	**L/ R**	**L**	**W**	**L/W**	**II**	**III**	**IV**	**III/II**	**III/IV**	**II-III**	**III-IV**	**II-IV**	**te**	**M**
PC-TH-2-**11**	1.5	L	47.70	31.10	1.53	21.00	25.40	36.30	1.21	0.70	20.96	23.08	44.04	13.00	0.42
PC-TH-2-**12**	1.5	R	46.60	28.00	1.66	19.40	26.20	34.10	1.35	0.77	26.82	18.70	45.52	14.60	0.52
PC-TH-2-**13**	1.5	L	43.10	29.60	1.46	20.20	25.30	34.40	1.25	0.74	31.38	24.37	55.75	10.60	0.36
PC-TH-2-**14**	1.5	R	45.60	32.20	1.42	20.20	25.40	35.80	1.26	0.71	32.15	27.21	59.36	11.80	0.37
PC-TH-2-**15**	1	L	n/a	n/a	n/a	n/a	n/a	n/a	n/a	n/a	n/a	n/a	n/a	n/a	n/a
***AVERAGE***	***1.4***		*45.75*	*30.23*	*1.52*	*20.20*	*25.58*	*35.15*	*1.27*	*0.73*	*27.83*	*23.34*	*51.17*	*12.50*	*0.42*

All lengths were measured in cm. Divarication angles were measured in degrees. All track measurements were rounded to two decimal places.

**Table 5:** Trackway measurements for PC-TH-1.

Specimen	L/ R	L	h	P	λ	WAP	αR	αL	γ
PC-TH-1-**06**	L	n/a	n/a	n/a	n/a	n/a	n/a	n/a	n/a
PC-TH-1-**07**	R	45.50	1.82	1.47	n/a	15.70	n/a	6.15	167.73
PC-TH-1-**08**	L	45.20	1.81	1.48	2.93	24.60	6.11	n/a	160.76
PC-TH-1-**09**	R	43.00	1.72	1.21	2.90	18.40	n/a	9.69	165.35
PC-TH-1-**10**	L	45.80	1.83	1.43	2.87	n/a	7.39	n/a	n/a
***AVERAGE***		***44.88***	*1.80*	***1.40***	***2.90***	***19.57***	***6.75***	***7.92***	***164.61***

All values were rounded to two decimal places. Total track length (L) and width of pes angulation (WAP) were measured in cm. Hip height (h), pace (P) and stride (λ) lengths were measured in m. Right or left angles of rotation (αR or αL respectively) and pace angulation (γ) were measured in degrees.

**Table 6:** Trackway measurements for PC-TH-2.

Specimen	L/ R	L	h	P	λ	WAP	αR	αL	γ
PC-TH-2-**11**	L	47.70	1.91	n/a	n/a	4.30	n/a	n/a	n/a
PC-TH-2-**12**	R	46.60	1.86	1.57	n/a	6.80	n/a	1.62	176.59
PC-TH-2-**13**	L	43.10	1.72	1.42	2.98	n/a	1.79	n/a	174.59
PC-TH-2-**14**	R	45.60	1.82	1.46	2.88	n/a	n/a	2.66	n/a
PC-TH-2-**15**	L	n/a	n/a	n/a	n/a	n/a	n/a	n/a	n/a
***AVERAGE***		***45.75***	***1.83***	***1.48***	***2.93***	***5.55***	***1.79***	***2.14***	***175.59***

All values were rounded to two decimal places. Total track length (L) and width of pes angulation (WAP) were measured in cm. Hip height (h), pace (P) and stride (λ) lengths were measured in m. Right or left angles of rotation (αR or αL respectively) and pace angulation (γ) were measured in degrees.

**Table 7:** Stride and overall trackway velocities and gait ratios for PC-TH-1.

Track λ	L/ R	L	L-SD	h	λ	λ/h	AX-V	RT-V	AX-V-SD	RT-V-SD
PC-TH-1-**08-06**	L	43.50	0.55	1.77	2.93	1.66	2.42	2.11	0.00	0.00
PC-TH-1-**09-07**	R	44.57	0.10	1.77	2.90	1.64	2.37	2.14	0.00	0.00
PC-TH-1-**10-08**	L	44.67	0.18	1.77	2.87	1.62	2.33	2.19	0.00	0.00
***AVERAGE***		***44.24***	***0.53***	***1.77***	***2.90***	***1.64***	***2.37***	***2.15***	***0.04***	***0.03***

All values were rounded to two decimal places. Track stride (track λ) denotes the tracks from which a stride was measured between. ‘L/ R’ denoted whether a stride originated from the left or right foot. Track length (L) represented an average value of at least three track lengths from within the denoted stride and were measured in cm. These measurements can be inferred from two tracks if an alternating track is missing from within the stride. Hip height (h), and stride length (λ) were measured in metres. Hip height represented an average value obtained from stride averages. λ/h ratios represented an estimated gait. Velocity (V) was measured in m/s. Standard deviation (SD) of track length and velocity are also reported. AX velocity follows [69], while RT velocity follows [70].

**Table 8:** Stride and overall trackway velocities and gait ratios for PC-TH-2.

Track λ	L/ R	L	L-SD	h	λ	λ/h	AX-V	RT-V	AX-V-SD	RT-V-SD
PC-TH-2-**13-11**	L	45.80	0.12	1.82	2.98	1.64	2.41	2.05	0.00	0.00
PC-TH-2-**14-12**	R	45.10	0.12	1.82	2.88	1.58	2.27	2.18	0.00	0.00
***AVERAGE***		***45.45***	***0.35***	***1.82***	***2.93***	***1.61***	***2.34***	***2.11***	***0.07***	***0.06***

Note, PC-TH-2-15 was not clearly exposed when fieldwork took place for a stride measurement to be taken and would have been omitted due to incompleteness. All values were rounded to two decimal places. Track length was measured in cm. Hip height (h), and stride length (λ) were measured in metres. Velocity was measured in m/s. AX velocity follows [69], while RT velocity follows [70].

**Table 9:** Track measurements for PC-TH-4.

						Digit length (DL)			DL ratios		Divarication angles				
**Specimen**	**PG**	**L/ R**	**L**	**W**	**L/W**	**II**	**III**	**IV**	**III/II**	**III/IV**	**II-III**	**III-IV**	**II-IV**	**te**	**M**
PC-TH-4-**23**	0.5	R	52.20	41.80	1.25	n/a	n/a	n/a	n/a	n/a	n/a	n/a	n/a	20.80	0.50
PC-TH-4-**24**	1	R	47.60	33.90	1.40	27.60	29.30	38.30	1.06	0.77	26.82	23.77	50.59	13.40	0.40
PC-TH-4-**25**	1	L	51.80	39.70	1.30	28.30	33.70	36.20	1.19	0.93	24.86	32.35	57.21	21.20	0.53
PC-TH-4-**26**	0.5	R	44.50	33.70	1.32	25.20	35.90	33.10	1.42	1.08	20.25	18.34	38.59	13.30	0.39
PC-TH-4-**27**	0.5	L	51.40	36.40	1.41	27.50	35.50	38.10	1.29	0.93	25.03	30.61	55.64	18.00	0.49
PC-TH-4-**28**	1	R	48.00	37.40	1.28	23.70	30.40	32.40	1.28	0.94	19.01	34.74	53.75	19.90	0.53
PC-TH-4-**29**	1	L	53.40	43.10	1.24	29.10	31.10	35.70	1.07	0.87	29.12	25.48	54.60	23.00	0.53
PC-TH-4-**30**	0	R	n/a	n/a	n/a	n/a	n/a	n/a	n/a	n/a	n/a	n/a	n/a	n/a	n/a
***AVERAGE***	*0.69*		***49.84***	***38.00***	***1.32***	***26.90***	***32.65***	***35.63***	***1.22***	***0.92***	***24.18***	***27.55***	***51.73***	***18.51***	***0.48***

All lengths were measured in cm. Divarication angles were measured in degrees. All track measurements were rounded to two decimal places.

The tracks are characterised by similar overall and digit lengths (Fig 10, Tables 3–4). PC-TH-1 track lengths vary between 43.0-45.8 cm (average 44.9 cm), while PC-TH-2 equivalents range between 43.1-47.7 cm (average 45.8 cm). Based on these track lengths, the estimated trackmaker hip height of PC-TH-1 (1.80 m) is almost identical to PC-TH-2 (1.83 m). The PC-TH-1 and 2 tracks also possess longer than wide l/w ratios (1.36-1.42 in PC-TH-1 and 1.42-1.66 in PC-TH-2). Although subtle, the digit ii-iv divarication angles are on average larger in PC-TH-1 (55.1°) than PC-TH-2 (51.2°).

The distal region of digits ii-iii of PC-TH-1-08 appear to overprint into the lower margin of digit ii and heel region of PC-TH-2-12 (Fig 10B). This cross-cutting relationship and the presence of more clearly defined ripples within the PC-TH-2 track impressions suggests that the PC-TH-1 trackway was impressed slightly later than PC-TH-2. Their similar track margin definitions further imply that both trackways were likely impressed within a similar timeframe of one another. PC-TH-2-15 also overprints the worn displacement rim of sauropod track PC-SA-2-85 and further suggests the PC-TH-2 trackmaker traversed the lagoon margin soon after PC-SA-2 was registered and before PC-TH-1 was registered. These relationships demonstrate that the footprints in Section One are at least a little bit time-averaged and the trackmakers may not have crossed the same physical space at the same time.

Both trackways also exhibit relatively consistent pace and stride lengths (Tables 5–6). On average, PC-TH-1 paced (1.40 m) and strode (2.90 m) similar distances to PC-TH-2, which paced at 1.48 m and strode at 2.93 m. The estimated velocities of PC-TH-1 are on average between 2.15-2.37 m/s (7.74-8.53 km/h) and very similar to that of PC-TH-2 at 2.11-2.34 m/s (7.60-8.42 km/h) (Tables 7–8). Overall, based on the gait parameters of [69], PC-TH-1 (1.62-1.66) and PC-TH-2 (1.58-1.64) both demonstrate walking gaits. Despite similarity in track morphology and velocity, both trackways are distinguished by contrasting WAP. The PC-TH-1 trackmaker overall paced at a much wider WAP (15.7-24.6 cm) than PC-TH-2 (4.3-6.8 cm). Regardless, both trackmakers are considered to have walked at a relatively narrow gauge.

#### PC-TH-4.

PC-TH-4 consists of eight large to giant-sized tracks, with seven clearly tridactyl, spanning over 12.2 m oriented 237° (Fig 11, Tables 9–11). All except PC-TH-4-23 are in concave epirelief. The tracks almost bear the same direction as palaeocurrents indicated by ripples (228.2°). Due to present-day erosion, tracks range from worn surface tracks to transmitted tracks and score low preservation grades between 0-1 (average 0.69). The overall lower preservation grade of this trackway may explain why length and width measurements appear larger than tracks with more crisply defined margins in PC-TH-1 and 2. Although the measurements of the PC-TH-4 tracks were not used to characterise morphotype-1a, the tracks were referred to the morphotype based on shared characteristics.

**Table 10:** Trackway measurements for PC-TH-4.

Specimen	L/ R	L	h	P	λ	WAP	αR	αL	γ
PC-TH-4-**23**	R	52.20	2.09	n/a	3.00	n/a	n/a	n/a	n/a
PC-TH-4-**24**	R	47.60	1.90	1.48	2.88	n/a	n/a	13.12	n/a
PC-TH-4-**25**	L	51.80	2.07	1.48	2.88	33.50	7.37	n/a	153.81
PC-TH-4-**26**	R	44.50	1.78	1.42	2.83	19.00	n/a	12.52	164.92
PC-TH-4-**27**	L	51.40	2.06	1.48	2.91	30.70	9.42	n/a	155.45
PC-TH-4-**28**	R	48.00	1.92	1.47	2.96	24.10	n/a	9.46	161.15
PC-TH-4-**29**	L	53.40	2.14	1.53	n/a	24.10	9.10	n/a	161.45
PC-TH-4-**30**	R	n/a	n/a	n/a	n/a	n/a	n/a	n/a	n/a
***AVERAGE***		***49.84***	***1.99***	***1.48***	***2.91***	***26.28***	***8.63***	***11.70***	***159.36***

All values were rounded to two decimal places. Total track length (L) and width of pes angulation (WAP) were measured in cm. Hip height (h), pace (P) and stride (λ) lengths were measured in m. Right or left angles of rotation (αR or αL respectively) and pace angulation (γ) were measured in degrees.

**Table 11:** Stride and overall trackway velocities and gait ratios for PC-TH-4.

Track λ	L/ R	L	L-SD	h	λ	λ/h	AX-V	RT-V	AX-V-SD	RT-V-SD
PC-TH-4-23-24	R	49.90	0.20	1.97	3.00	1.52	2.22	2.00	0.01	0.01
PC-TH-4-24-26	R	47.97	2.20	1.97	2.88	1.47	2.08	1.88	0.00	0.00
PC-TH-4-25-27	L	49.23	0.05	1.97	2.88	1.46	2.07	1.87	0.00	0.00
PC-TH-4-26-28	R	47.97	2.20	1.97	2.83	1.44	2.01	1.82	0.01	0.01
PC-TH-4-27-29	L	50.93	2.20	1.97	2.91	1.48	2.11	1.91	0.00	0.00
PC-TH-4-28-30	R	50.70	1.56	1.97	2.96	1.51	2.17	1.97	0.00	0.00
***AVERAGE***		***49.45***	***1.30***	***1.98***	***2.91***	***1.47***	***2.10***	***1.90***	***0.07***	***0.06***

Due to a missing track between the PC-TH-4-23-24 stride, average measurements of only two tracks were taken. The average length for the PC-TH-4-28-30 stride does not include PC-TH-4-30 due to its poorly defined margins. All values were rounded to two decimal places. Track length was measured in cm. Hip height (h), and stride length (λ) were measured in metres. Velocity was measured in m/s. AX velocity follows [69], while RT velocity follows [70].

Measurements such as low l/w ratios (1.24-1.41) and low-moderate mesaxony (0.39-0.53) are consistent with morphotype-1a (Table 9). Similarly, most digit ii-iv divarication angles are within the 40°-60° morphotype range (50.6°-57.2°), apart from PC-TH-4-26 (38.6°). Though phalangeal pads were difficult or impossible to distinguish within this trackway, faint digit margins indicate the tracks possessed broad digits with tapering, elongated ungual marks (Fig 12). Further morphotype-1a similarities include a declining digit length between digits iv to ii.

The trackway exhibits relatively consistent pace (1.42-1.53 m) and stride (2.83-3.00 m) lengths, with generally subtly larger right pace angulations (161.2°-164.9°) than left equivalents (153.8°-161.5°) (Table 10). Right track WAPs meanwhile are generally smaller (19.0-24.1 cm) than left equivalents (24.1-33.5 cm). On average, the trackmaker exhibited a relatively steady walking gait (1.47) travelling between 1.90-2.10 m/s (6.84-7.56 km/h) (Table 11).

#### PC-TH-5.

PC-TH-5 is composed of ten large-giant sized tridactyl tracks, with an overall bearing of 7.2° over 15.2 m (Fig 13, Tables 12–14). All except PC-TH-5-38 and 39 are in concave epirelief. Track preservation grades are mostly between 0-1 due to poorly defined digit margins. As in PC-TH-4, track length and width appear subtly exaggerated. This exaggeration may have contributed to relatively low l/w ratios (Table 12).

**Table 12:** Track measurements for PC-TH-5.

						Digit length (DL)			DL ratios		Divarication angles				
**Specimen**	**PG**	**L/ R**	**L**	**W**	**L/W**	**II**	**III**	**IV**	**III/II**	**III/IV**	**II-III**	**III-IV**	**II-IV**	**te**	**M**
PC-TH-5-**31**	0.5	L	50.70	41.50	1.22	n/a	n/a	n/a	n/a	n/a	37.10	48.82	85.92	22.50	0.54
PC-TH-5-**32**	0.5	R	46.20	41.80	1.11	26.00	33.20	33.80	1.28	0.98	29.97	35.05	65.02	14.20	0.34
PC-TH-5-**33**	1	L	51.80	45.20	1.15	28.90	36.70	45.90	1.27	0.80	27.76	32.32	60.08	14.80	0.33
PC-TH-5-**34**	1	R	51.20	42.60	1.20	25.70	32.90	39.90	1.28	0.82	33.42	37.16	70.58	17.30	0.41
PC-TH-5-**35**	1	L	55.10	42.50	1.30	n/a	n/a	n/a	n/a	n/a	30.97	32.29	63.26	21.60	0.51
PC-TH-5-**36**	1	R	46.50	38.90	1.20	26.10	28.80	35.00	1.10	0.82	38.12	36.71	74.83	18.50	0.48
PC-TH-5-**37**	1	L	49.00	42.80	1.14	27.80	28.60	37.00	1.03	0.77	18.57	51.21	69.78	15.80	0.37
PC-TH-5-**38**	0.5	L	46.90	44.00	1.07	n/a	n/a	n/a	n/a	n/a	35.59	49.58	85.17	18.10	0.41
PC-TH-5-**39**	0	R	n/a	n/a	n/a	n/a	n/a	n/a	n/a	n/a	n/a	n/a	n/a	n/a	n/a
PC-TH-5-**40**	1.5	R	46.30	36.20	1.28	22.90	32.60	31.00	1.42	1.05	16.76	36.96	53.72	18.20	0.50
***AVERAGE***	*0.8*		***49.30***	***41.72***	***1.18***	***26.23***	***32.13***	***37.10***	***1.23***	***0.88***	***29.81***	***40.01***	***69.82***	***17.89***	***0.43***

All lengths were measured in cm. Divarication angles were measured in degrees. All track measurements were rounded to two decimal places.

**Table 13:** Trackway measurements for PC-TH-5.

Specimen	L/ R	L	h	P	λ	WAP	αR	αL	γ
PC-TH-5-**31**	L	50.70	2.03	1.58	2.89	n/a	20.85	n/a	n/a
PC-TH-5-**32**	R	46.20	1.85	1.48	2.63	48.90	n/a	21.78	142.41
PC-TH-5-**33**	L	51.80	2.07	1.40	2.52	58.40	18.57	n/a	131.87
PC-TH-5-**34**	R	51.20	2.05	1.30	2.34	49.20	n/a	26.00	137.16
PC-TH-5-**35**	L	55.10	2.20	1.33	2.56	59.70	22.09	n/a	125.88
PC-TH-5-**36**	R	46.50	1.86	1.43	n/a	51.20	n/a	20.96	136.27
PC-TH-5-**37**	L	49.00	1.96	n/a	2.56	n/a	n/a	n/a	n/a
PC-TH-5-**38**	L	46.90	1.88	1.51	n/a	n/a	n/a	n/a	n/a
PC-TH-5-**39**	R	n/a	n/a	n/a	2.60	n/a	n/a	n/a	n/a
PC-TH-5-**40**	R	46.30	1.85	n/a	n/a	n/a	n/a	n/a	n/a
***AVERAGE***		***49.30***	***1.97***	***1.43***	***2.59***	***53.48***	***20.50***	***22.91***	***134.72***

All values were rounded to two decimal places. Total track length (L) and width of pes angulation (WAP) were measured in cm. Hip height (h), pace (P) and stride (λ) lengths were measured in m. Right or left angles of rotation (αR or αL respectively) and pace angulation (γ) were measured in degrees.

**Table 14:** Stride and overall trackway velocities and gait ratios for PC-TH-5.

Track λ	L/ R	L	L-SD	h	λ	λ/h	AX-V	RT-V	AX-V-SD	RT-V-SD
PC-TH-5-31-33	L	49.57	0.00	1.99	2.89	1.46	2.07	1.87	0.12	0.10
PC-TH-5-32-34	R	49.73	0.01	1.99	2.63	1.32	1.76	1.59	0.00	0.00
PC-TH-5-33-35	L	52.70	9.45	1.99	2.52	1.27	1.64	1.48	0.01	0.00
PC-TH-5-34-36	R	50.93	1.71	1.99	2.34	1.18	1.46	1.32	0.07	0.06
PC-TH-5-35-37	L	50.20	0.33	1.99	2.56	1.29	1.69	1.53	0.00	0.00
PC-TH-5-37-38	L	47.95	2.81	1.99	2.56	1.29	1.68	1.52	0.00	0.00
PC-TH-5-39-40	R	46.30	11.06	1.99	2.60	1.31	1.73	1.57	0.00	0.00
***AVERAGE***		***49.63***	***1.90***	***1.99***	***2.59***	***1.30***	***1.72***	***1.55***	***0.17***	***0.15***

Due to missing tracks, average measurements of only two tracks informed the metrics of the PC-TH-5-37-38 and PC-TH-5-39-40 strides. All values were rounded to two decimal places. Track length was measured in cm. Hip height (h), and stride length (λ) were measured in metres. Velocity was measured in m/s. AX velocity follows [69], while RT velocity follows [70].

In contrast to most PC-TH-5 tracks, PC-TH-5-40 (preservation grade =  1.5) possesses clearly defined digit and phalangeal pad margins. PC-TH-5-40 was used to diagnose morphotype-1a while the remaining PC-TH-5 tracks were referred to morphotype-1a (Fig 14). The digit ii-iv divarication angle in most PC-TH-5 tracks were larger than those used to distinguish morphotype-1a, i.e., (53.7°-85.9°), possibly in part due to their eroded, more poorly defined margins, or a specific mode of locomotion. The largest interdigital angles were predominantly measured between digits iii-iv. The largest difference is recorded in PC-TH-5-37, which featured digits iii-iv angled at 51.2° and digits ii-iii at 18.6°.

The trackway meanwhile exhibits natural fluctuations across an overall linear path (Table 13). Stride (2.34-2.89 m) and pace (1.30-1.58 m) exhibit greater ranges compared to other morphotype-1a trackways. These lengths appear to reduce between PC-TH-5-31 and 34 and increase thereafter. This fluctuation is therefore reflected in the stride/hip height ratio (1.46-1.18) and estimated velocities: 2.07-1.46 m/s =  7.45-5.26 km/h according to the equation in [69]; or 1.87-1.32 m/s =  6.73-4.75 km/h according to the equation in [70]. Regardless of velocity equation, the results highlight a walking gait (Table 14).

#### PC-TH-A-4.

PC-TH-A-4 is composed of two tridactyl tracks and extends over 3.41 m at 280.2° (Fig 15; S6 Fig, Table 15). PC-TH-A-4-49 is a shallow concave epirelief track with a preservation grade of 1.5. The track features well-defined digit margins, phalangeal pads, and ungual marks and was used to define morphotype-1a. The track features an enlarged heel region which extends its overall length to 53.2 cm, a 34.4 cm width, 1.55 l/w ratio and moderate mesaxony (0.50). These metrics, apart from l/w ratios, are subtly larger in PC-TH-A-4-50, which was not used to define morphotype-1a due to poorly defined digit margins toward the heel (preservation grade =  1). Despite this, the digit ii-iv divarication angle is larger in PC-TH-A-4-50 (59.5°) than PC-TH-A-4-49 (44.1°). In PC-TH-A-4-49, the digit ii-iii interdigital angle is unusually < 10° (5.9°).

**Table 15:** Track measurements for PC-TH-A-4.

			In cm			Digit length (DL) in cm			DL ratios		Divarication angles			In cm	
**Specimen**	**PG**	**L/ R**	**L**	**W**	**L/W**	**II**	**III**	**IV**	**III/II**	**III/IV**	**II-III**	**III-IV**	**II-IV**	**te**	**M**
PC-TH-A-4-**49**	1.5	L	53.20	34.40	1.55	24.40	32.40	33.90	1.33	0.96	5.92	38.19	44.11	17.30	0.50
PC-TH-A-4-**50**	1	R	50.90	38.20	1.33	24.20	37.70	35.60	1.56	1.06	24.33	35.16	59.49	19.90	0.52
***AVERAGE***	***1.25***		*52.05*	*36.30*	*1.44*	*24.30*	*35.05*	*34.75*	*1.44*	*1.01*	*15.13*	*36.68*	*51.80*	*18.60*	*0.51*

All lengths were measured in cm. Divarication angles were measured in degrees. All track measurements were rounded to two decimal places.

#### Isolated tracks.

Most isolated tracks referred to morphotype-1a were generally incomplete or lacked clear margins. These tracks ranged in preservation grade between 0-2 (Fig 16, Table 16). Although isolated, such tracks are documented as they provide an indication of the number of theropod trackmakers, their varied directions, and close co-occurrences to sauropod trackmakers. PC-TH-I-46 occurs immediately ahead of the trajectory of sauropod trackway PC-SA-3 and bears 345° (Fig 16A-D). Unusually, a single unidentified bone is preserved within the heel region. PC-TH-I-16 occurs between the gauge margins of PC-SA-1 and bears 42° (Fig 16E-H). Exceptional tracks such as PC-TH-I-01 retain clear morphological detail and are used to define morphotype-1a (Fig 16I-L). PC-TH-I-01 bears 210° and is situated closest to the present-day high-tide mark in bed 2. The track is similarly preserved to some PC-TH-1 and 2 tracks with sharp, elongated ungual marks and ripples intruding the heel region. A ‘2:3:4’ phalangeal pad formula was discerned from a clear digit margin. The track is 46.3 cm long by 38.1 cm wide (l/w ratio 1.22) with a weak 0.38 mesaxony. The track features some of the most narrowly divaricated digits of the morphotype: digits ii-iii are divaricated by 18.0°, while digits iii-iv are 34.4° (total divarication angle 52.4°).

**Table 16:** Track measurements for selected isolated tracks.

						Digit length (DL)			DL ratios		Divarication angles				
**Specimen**	**PG**	**L/ R**	**L**	**W**	**L/W**	**II**	**III**	**IV**	**III/II**	**III/IV**	**II-III**	**III-IV**	**II-IV**	**te**	**M**
PC-TH-I-**01**	2	R	46.34	38.12	1.22	25.71	28.90	36.18	1.12	0.80	18.01	34.43	52.44	14.43	0.38
PC-TH-I-**16**	0.5	?	n/a	n/a	n/a	n/a	n/a	n/a	n/a	n/a	n/a	n/a	n/a	n/a	n/a
PC-TH-I-**41**	2	?	41.20	34.80	1.18	20.70	27.80	30.70	1.34	0.91	26.50	32.20	58.70	14.10	0.41
PC-TH-I-**46**	1	L?	42.90	29.30	1.46	n/a	n/a	n/a	n/a	n/a	24.40	37.51	61.91	17.20	0.59

All lengths were measured in cm. Divarication angles were measured in degrees. All track measurements were rounded to two decimal places.

Another clearly preserved track is PC-TH-I-41 (Fig 16M-N, Table 16). PC-TH-I-41 is a convex epirelief tridactyl track which bears 282° and scores a preservation grade of 2. As a large boulder covered the anterior portion of the track during later fieldwork, a photogrammetric model could not be produced. Despite this, approximate track measurements were taken from photographs. The track metrics occupy the extreme ranges of morphotype-1a and include a ~ 41.2 cm length and ~ 34.8 cm width (l/w ratio 1.18) with a 0.41 mesaxony and digit ii-iv divarication angle of ~ 58.7°. Like morphotype-1a, PC-TH-I-41 features clearly defined phalangeal pads and long ungual marks and digits with sharp margins. The lateral digits however feature sigmoidal curvature. Pad Piv1 furthermore is poorly defined.

### Morphotype-1b

**Fig 17:**
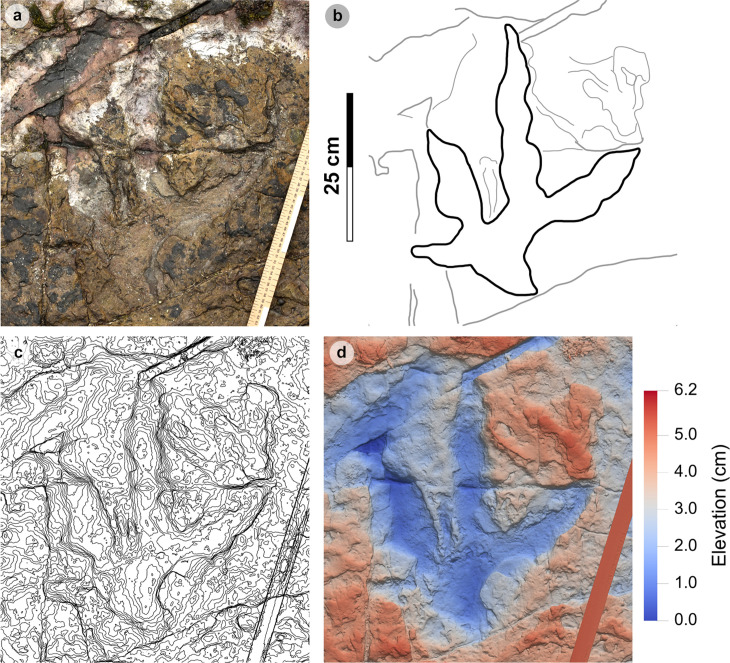
Digital representations of PC-TH-I-55. (A) Textured orthophoto, (B) outline, (C) contour, (D) DEM. Unlike morphotype-1a tracks, PC-TH-I-55 possesses an anteromedially oriented digit i ungual impression. The digits are slenderer than morphotype-1a with well-defined phalangeal pad margins.

**Fig 18:**
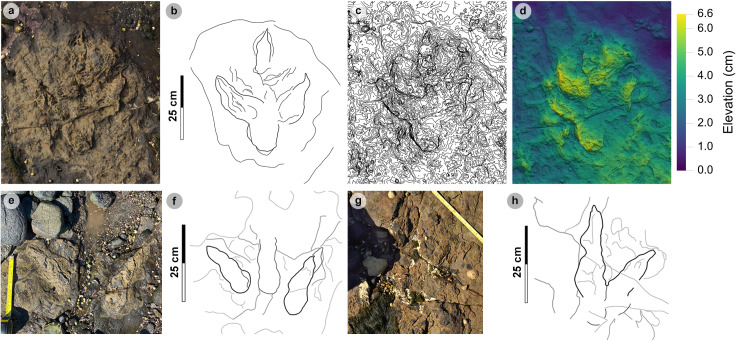
Photographic and digital representations of PC-TH-3 tracks. (A-D) From left to right, textured orthophoto, outline, contour map, and DEM of PC-TH-3-20 - a complete convex epirelief track with a ‘U’ shaped heel and poorly defined digit margins. (E-F) Photograph and outline of PC-TH-3-22, a partial convex epirelief track composed of lateral digits. (G-H) Photograph and outline of PC-TH-3-19, a second partial concave epirelief track with poorly defined digit ii and iii margins. The latter two tracks could not be attributed to morphotype-1b due to their incompleteness, isolated digits and absent digit i impression. Despite this, the tracks are described due to their occurrence in a trackway sequence with PC-TH-3-20.

**Material** – PC-TH-3-20, PC-TH-I-55 (Figs 17 and 18A-D; Table 17).

**Table 17:** Track measurements for morphotype-1b.

						Digit length (DL)			DL ratios		Divarication angles				
**Specimen**	**PG**	**L/ R**	**L**	**W**	**L/W**	**II**	**III**	**IV**	**III/II**	**III/IV**	**II-III**	**III-IV**	**II-IV**	**te**	**M**
PC-TH-3-**20**	1.5	L?	43.30	32.70	1.32	17.10	23.20	19.10	1.36	1.21	29.03	44.50	73.53	17.30	0.53
PC-TH-I-**55**	1.5	R	41.80	33.30	1.26	17.70	27.80	19.20	1.57	1.45	24.81	50.93	75.74	17.10	0.51
***AVERAGE***	*1.5*		***42.55***	***33.00***	***1.29***	***17.40***	***25.50***	***19.15***	***1.46***	***1.33***	***26.92***	***47.72***	***74.64***	***17.20***	***0.52***

All lengths were measured in cm. Divarication angles were measured in degrees. All track measurements were rounded to two decimal places.

#### Diagnosis.

Differentiated from morphotype-1a with the presence of a tapering digit i and/or heel. Digits are broad and lack metatarsophalangeal pads. Digit i is inconsistently preserved. – i.e., phalangeal pads and ungual marks may be present. Digit ii and iv lengths are generally subequal in length. The digit ii-iv divarication angle is > 65°.

#### Description.

A large tridactyl track, between 41.8-43.3 cm long and 32.7-33.3 cm wide (l/w ratio 1.26-1.32). Tracks are moderately mesaxonic (0.51-0.53) with moderately slender, distally tapering digits and narrow, long ungual marks. Digit margins are generally linear. Digits could possess clear phalangeal pad margins and subtly anterolateral oriented ungual marks. Tracks can possess a ‘X:2:3:3’ phalangeal pad formula. Digit iii is the longest digit. A digit i ungual mark may be present and laterally directed opposite a posterolaterally oriented subtriangular heel region. The digit ii-iv divarication angle ranges between 73.5°-75.7°.

### Morphotype-1b material description

#### PC-TH-I-55.

PC-TH-I-55 represents the clearest example of morphotype-1b, with a preservation grade of 1.5. Metrically, the track is distinguished by its low l/w ratio (1.26), moderate mesaxony (0.51), and pronounced acute divarication angle (75.7°) (Table 17). Morphologically, the track is distinguished from other morphotype-1 subgroup by lacking a digit iv metatarsophalangeal pad and featuring a anteromedially directed, triangular shaped digit i ungual mark impression (Fig 17). A second morphotype-1b track, PC-TH-3-20, forms a part of the PC-TH-3 trackway as discussed below.

#### PC-TH-3.

PC-TH-3 is an isolated trackway which extends over 4.93 m at 311.14° and is composed of four tracks – including a single morphotype-1b track (Figs 18, S9). Shales appear to obscure most of PC-TH-3-21. Due to the occurrence of incomplete tracks, trackway measurements were omitted. Although most tracks score low preservation grades (0-0.5), a single complete track diagnosed as morphotype-1b, PC-TH-3-20, is graded 1.5. The track measures 43.3 cm long by 32.7 cm wide (l/w ratio 1.32) with a 0.53 mesaxony. Like PC-TH-I-55, the digit iii-iv interdigital angle (44.5°) is greater than digit ii-iii (29.0°). Morphologically, the track possesses an extensive ‘U’ shaped heel region (Fig 18A-D). Although part of the same trackway as PC-TH-3-20, PC-TH-3-19 and PC-TH-3-22 morphologically differ with isolated digits and no digit i impression (Fig 18E-H). Due to this, while these tracks reflect characteristics of an alternative morphotype like morphotype-1c, they are unreferred. Their presence within this trackway suggests a single trackmaker could produce multiple track shapes.

### Morphotype-1c

**Fig 19:**
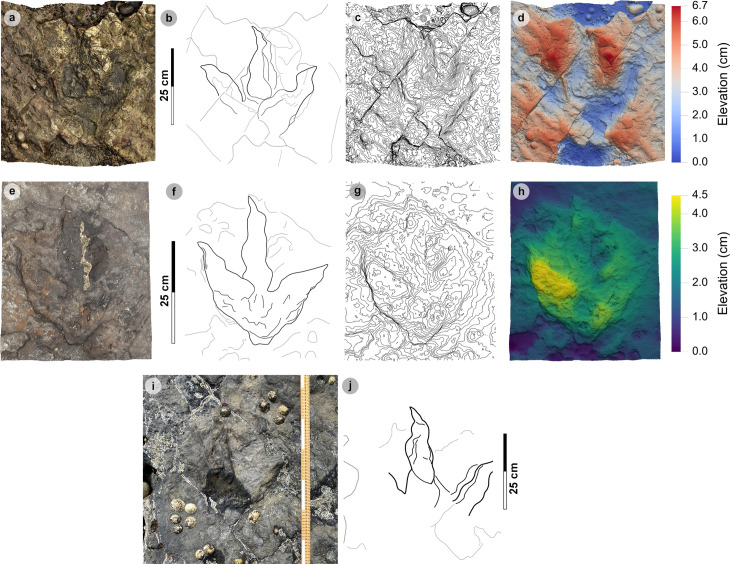
Photographic and digital representations of morphotype-1c tracks. From left to right, textured orthophoto, outline, contour map, and DEM. (A-D) PC-TH-I-56 was the only recorded concave epirelief morphotype-1c track. The track is situated in a slightly younger bed 2 horizon behind an igneous intrusion. (E-H) PC-TH-I-59 is distinguished as morphotype-1c due to its smaller track length, more slender digits, and wider > 60° digit ii-iv divarication angle. (I) Photograph and (J) outline of PC-TH-I-65. The track is referred to morphotype-1c and is partially metamorphosed. The track occurs in the same horizon as PC-TH-I-59.

**Material** – PC-TH-I-56, 59 (Fig 19, Table 18).

**Table 18:** Track measurements for morphotype-1c tracks.

						Digit length (DL)			DL ratios		Divarication angles				
**Specimen**	**PG**	**L/ R**	**L**	**W**	**L/W**	**II**	**III**	**IV**	**III/II**	**III/IV**	**II-III**	**III-IV**	**II-IV**	**te**	**M**
PC-TH-I-**56**	1.5	L	32.10	28.40	1.13	15.60	20.70	22.60	1.33	0.92	33.50	30.34	63.84	10.30	0.36
PC-TH-I-**59**	2	L	30.60	26.30	1.16	17.20	19.10	22.10	1.11	0.86	32.12	44.85	76.97	13.20	0.50
***AVERAGE***	*1.75*		***31.35***	***27.35***	***1.15***	***16.40***	***19.90***	***22.35***	***1.22***	***0.89***	***32.81***	***37.60***	***70.41***	***11.75***	***0.43***

All lengths were measured in cm. Divarication angles were measured in degrees. All track measurements were rounded to two decimal places.

#### Diagnosis.

Differentiated from morphotype 1a-b with ~ 30 cm track lengths and weak l/w ratios. Digits are slenderer with sub-parallel margins which taper toward narrow, subtriangular ungual marks. The digit iv metatarsophalangeal pad is not well defined. Digit ii-iv divarication angles are > 60°.

#### Description.

A large, tridactyl footprint between 30.6-32.1 cm long and 26.3-28.4 cm wide (l/w ratio 1.13-1.16). Mesaxony varies between 0.36-0.50. Digits are moderately slender with sub-parallel margins and distally tapered to narrow, long anteriorly-oriented ungual marks. Digit iv is subtly longer than digit iii, while digit ii is the shortest digit. Digit length ratios on average differ slightly from morphotype-1a; digit iii/ii (1.22) and digit iii/iv (0.89). Vague phalangeal pad margins exhibit a ‘2:3:4’ formula. Wide digit ii-iv divarication angles measure between 63.8°-77.0°.

### Morphotype-1c material description

#### Isolated tracks.

Morphotype-1c is known from two isolated tracks which occur in the lower portion of bed 2 (Fig 19, Table 18). PC-TH-I-56 is the only known concave epirelief morphotype-1c track and occurs in a slightly younger rippled bed 2 horizon than PC-TH-I-59 (Fig 19A-D). Due to wear, the track scores a preservation grade of 1.5. The shallowly convex epirelief PC-TH-I-59 features clear digit and phalangeal pad margins and scores a preservation grade of 2. The track is situated in an older bed 2 horizon which directly overlies the topmost, desiccated bed 1 horizon (Fig 19E-H). In the same horizon, PC-TH-I-65, a partial track, is referred to the morphotype because it possesses similar digit slenderness, narrow tapering digits, and subtle sigmoidal curvature to PC-TH-I-59 (Fig 19I-J). The track is partially metamorphosed on the edge of the wave cut platform.

### Morphotype-1d

**Fig 20:**
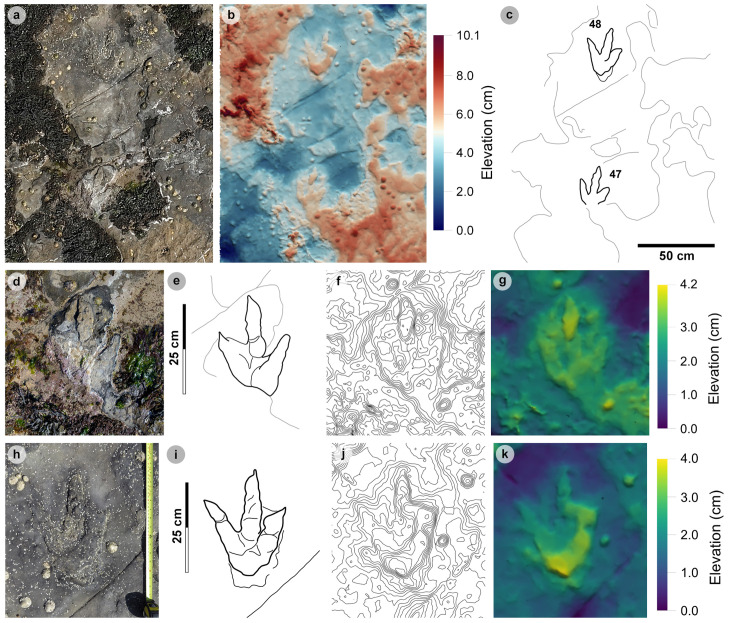
Overview of PC-TH-A-3. (A) Photograph cropped to model area, (B) DEM, (C) outline of PC-TH-A-3. Although characterised by a smaller < 30 cm overall length, the tracks of PC-TH-A-3 are classified under morphotype-1 due to a similar average l/w ratio (1.46), < 60° digit ii-iv divarication angle, and ‘2:3:4’ phalangeal pad configuration to morphotype-1a. From left to right, photographs, outline, contour map, and DEM of (D-G) PC-TH-A-3-47 and (H-K) PC-TH-A-4-48. Although worn, PC-TH-A-4-48 features clear phalangeal pad creases on digit ii. The creases of Piv1 and Piii3 are also visible.

**Material** – PC-TH-A-3-47, 48 (Fig 20, Table 19).

**Table 19:** Measurements for tracks used to define morphotype-1d.

			In cm			Digit length (DL) in cm			DL ratios		Divarication angles			In cm	
**Specimen**	**PG**	**L/ R**	**L**	**W**	**L/W**	**II**	**III**	**IV**	**III/II**	**III/IV**	**II-III**	**III-IV**	**II-IV**	**te**	**M**
PC-TH-A-3-**47**	1.5	R	26.30	17.60	1.49	13.50	20.00	18.40	1.48	1.09	24.48	29.17	53.65	9.80	0.56
PC-TH-A-3-**48**	1.5	L	27.60	19.40	1.42	12.90	17.80	21.30	1.38	0.84	24.65	33.61	58.26	9.80	0.51
***AVERAGE***	***1.5***		***26.95***	***18.50***	***1.46***	***13.20***	***18.90***	***19.85***	***1.43***	***0.96***	***24.57***	***31.39***	***55.96***	***9.80***	***0.53***

All lengths were measured in cm. Divarication angles were measured in degrees. All track measurements were rounded to two decimal places.

#### Diagnosis.

Differentiated from morphotype-1a-c with shorter track lengths < 30 cm and widths < 20 cm and absent digit i. Digits are slenderer, shorter in length, with smaller, narrow ungual marks. Phalangeal pads are typically longer than wide and ovular/subcircular in shape.

#### Description.

A medium-sized, tridactyl track between 26.3-27.6 cm long by 17.6-19.4 cm wide (l/w ratio 1.42-1.49). Mesaxonic indices are moderate (0.51-0.56). Digits are moderately slender with straight margins, gradually shortening distally toward short, narrow anteriorly-oriented ungual marks. The digit iii/ii ratio (1.43) is larger than its digit iii/iv equivalent (0.96). A ‘2:3:4’ phalangeal pad formula was distinguished through bulges on digit margins or weakly defined interdigital creases. The pads are typically longer than wide and ovular/subcircular in shape. Digit ii-iv divarication angles are < 60° (53.7°-58.3°).

### Morphotype-1d material description

#### PC-TH-A-3.

PC-TH-A-3 is composed of two shallow convex epirelief tridactyl tracks – the only representatives of morphotype-1d (Fig 20, Table 19). The tracks are situated in bed 2 in Section Two, bear 87.7°, with preservation grades of 1.5. PC-TH-A-3-47 is a right track, while PC-TH-A-3-48 is a left track. Compared to PC-TH-A-3-47, PC-TH-A-3-48 features a larger digit iii-iv interdigital angle (33.6°) over digits ii-iii (24.7°). In PC-TH-A-3-47, the digit ii-iii interdigital angle is almost identical to PC-TH-A-3-48 (24.5°), while its digit iii-iv interdigital angle (29.2°) is smaller than that of PC-TH-A-3-48. PC-TH-A-4-48 furthermore features faintly defined interdigital creases on digit ii and pads Piv1 and Piii3. Based on track length, the represented trackmaker stood at ~ 1.08 m to the hip. Across the visible track association, the trackmaker paced 89.7 cm.

### Morphotype-1 ichnotaxonomy

Ichnotaxonomically, despite differences in overall track length, morphotype-1 is metrically and morphologically most like *Megalosauripus* [72]. *Megalosauripus* is functionally tridactyl with an elongate ‘heel’, i.e., the digit iv metatarsophalangeal pad (Piv1), and features weak-moderate l/w ratios (~1.2) and mesaxonic indices (0.40) and digit ii-iv divarication angles < 70° [72–73]. *Megalosauripus* furthermore possesses a ‘2:3:4’ phalangeal pad digit configuration and broad to moderately slender digits with elongated, sharp ungual marks [59,73]. The phalangeal pads themselves are often sub-circular or oval-shaped and longer than wide [72]. Morphotypes 1a and d possess all these characteristics. In the Cleveland Basin, Yorkshire, UK, ‘morphotype Bxviii’ was likened by [74–75] to *Megalosauripus isp*. due to its relatively low l/w ratios (1.24-1.34) and digit ii-iv divarication angles (45°-70°). In Portugal, [76] described ~ 80 *Megalosauripus-*like tracks with similar l/w ratios (1.24-1.39) and interdigital angles < 40° (22°-40°). Further morphotype-1 similarities to *Megalosauripus* include large obtuse pace angulations in trackways, which overlap with the 140°-160° range proposed by [77]. *Megalosauripus uzbekistanicus* tracks also feature similar pace angulation ranges of 125°-175° [72].

Similarly weak l/w ratios and mesaxonic indices were reported in the Late Jurassic *Jurabrontes curtedulensis* [59], which differ from morphotype-1 with bulkier, tear-drop shaped digits. The Late Jurassic *Iberosauripus grandis* is similar to *Jurabrontes* but differs with bulkier digits [78]. Other similar ichnotaxa include the Early Jurassic *Eubrontes giganteus*, which possess l/w ratios (1.40-1.50) and mesaxonic indices (0.58) within the range of some morphotype-1 tracks [61,79]. However, unlike *Eubrontes*, morphotype-1 possesses parallel-sided digit margins (not spindle-shaped) and a ‘2:3:4’ phalangeal pad formula (instead of ‘3:3:4’) [72].

Although most morphotype-1 tracks are characterised by digit ii-iv divarication angles < 60°, some like morphotype-1c, possess subtly slenderer digits and larger divarication angles like the Early Jurassic *Kayentapus*. Type tracks from North America reassessed by [32] from [80] possessed a subtly broader ~ 63° digit ii-iv divarication angle. These higher divarication angles contribute to low l/w ratios [79]. Morphotype-1 tracks with large digit ii-iv divarication angles > 70°, such as those of PC-TH-5, could reflect a subtle change in substrate composition or locomotion. Type *Kayentapus* tracks are further distinguished from morphotype-1 by generally slenderer, more digitigrade digits, with a detached or unimpressed Piv1 [81]. Morphotype-1c, although ~ 30 cm in length, is more similar in digit thickness and length to *Megalosauripus isp*. tracks reported from Portugal and Morocco – particularly morphotype 2c [72,82].

In contrast to most morphotype-1 tracks, morphotype-1b additionally possesses a subtriangular tapering heel region, with an anteromedially oriented digit i ungual mark on PC-TH-I-55 (Fig 17). These morphologies resemble those of the Early Cretaceous *Bueckeburgichnus maximus* [83]. However, rather than decrease in thickness between digits ii-iv, PC-TH-I-55 differs from *Bueckeburgichnus maximus* with relatively uniform digit slenderness. Furthermore, like *Megalosauripus*, oval-shaped and longer than wide phalangeal pads are present across digits ii-iv rather than in digit ii alone in *Bueckeburgichnus maximus*. The morphotype-1b characters more closely resemble type ichnospecies *Megalosauripus* tracks (*Megalosauripus uzbekistanicus*) identified by [73], which could also possess phalangeal pads, a heel impression and digit i ungual impression. These morphologies of morphotype-1b, in addition to its shorter digit lengths and wide digit ii-iv divarication angles compared to morphotype-1a, may have arisen due to deeper foot penetration into a softer substrate [84]. The extended heel impression on PC-TH-3-20, which resembles *Bueckeburgichnus maximus*, may have registered due to moderate sediment saturation. This is emphasised by large, shallow posterior displacement rims. These extend across a larger area compared to anterior displacement rims (Fig 18A-D). This may emphasise the longer duration to which the autopodia remained in contact with the sediment upon registration and a subtle posterior slide when removed *sensu* [85].

Overall, morphotype-1 is considered *Megalosauripus isp.* due to the possession of parallel-sided digit margins, broad digits, weak-moderate l/w ratios and mesaxony, and ‘2:3:4’ phalangeal pad formula. The impression of digit i in type *Megalosauripus* tracks and morphotype-1b demonstrates the variability of tracks, potentially due to substrate conditions. The presence of tracks with similar morphologies and divarication angles larger than typical *Megalosauripus* tracks, i.e., morphotype-1c and referred morphotype-1a tracks, may reflect on substrate or behaviour. Although morphotype-1c-d are smaller than typical *Megalosauripus* tracks, their possession of previously mentioned *Megalosauripus*-like metrics and morphology cannot be ignored.

### Morphotype-2

**Material** – PC-SA-1-69, 71-73, 75-84, PC-SA-2-85, 88, 90, PC-SA-I-101, PC-SA-3-102, 104-106, 108, 110-111, PC-SA-5-127-128 (Figs 21-28, Tables 20–29).

**Fig 21:**
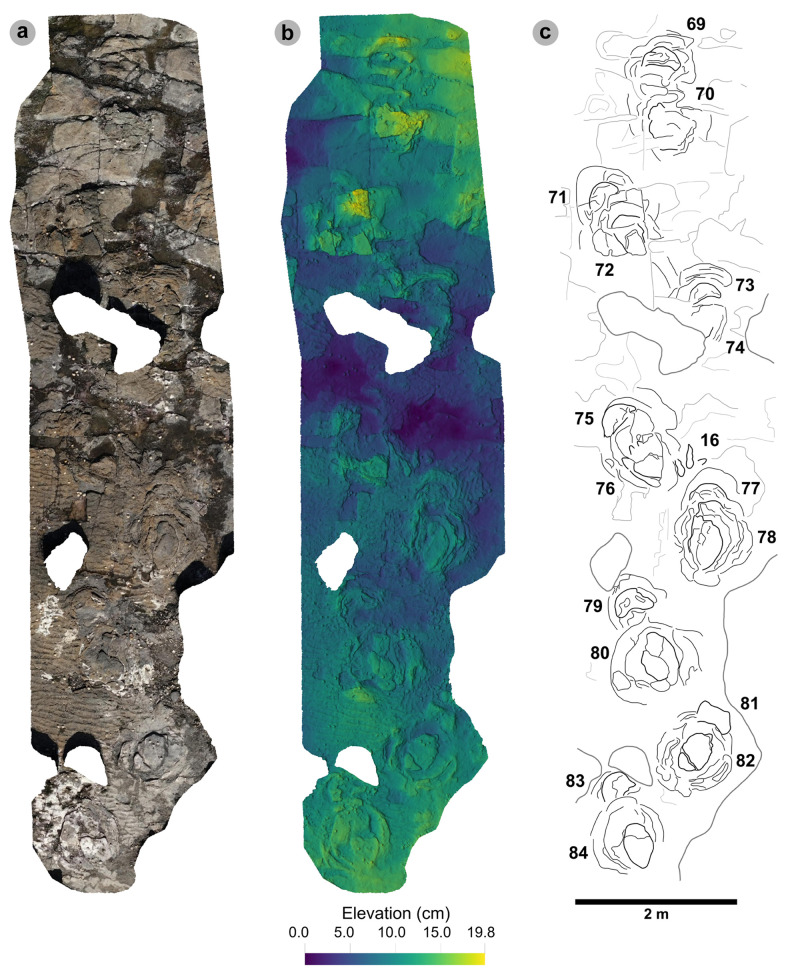
Overview of PC-SA-1. (A) Textured orthophoto with software-based shadowing, (B) DEM, (C) outline. Unlike PC-SA-1-82, most pes tracks lack digits and pads. Each are surrounded by wide, rippled displacement rims. Manus tracks are crescentic and possess a posterolaterally oriented pollex. The tracks gradually become increasingly scoured toward the present-day shoreline due to bed dip, exposing under tracks impressed down into bed 1.

**Fig 22:**
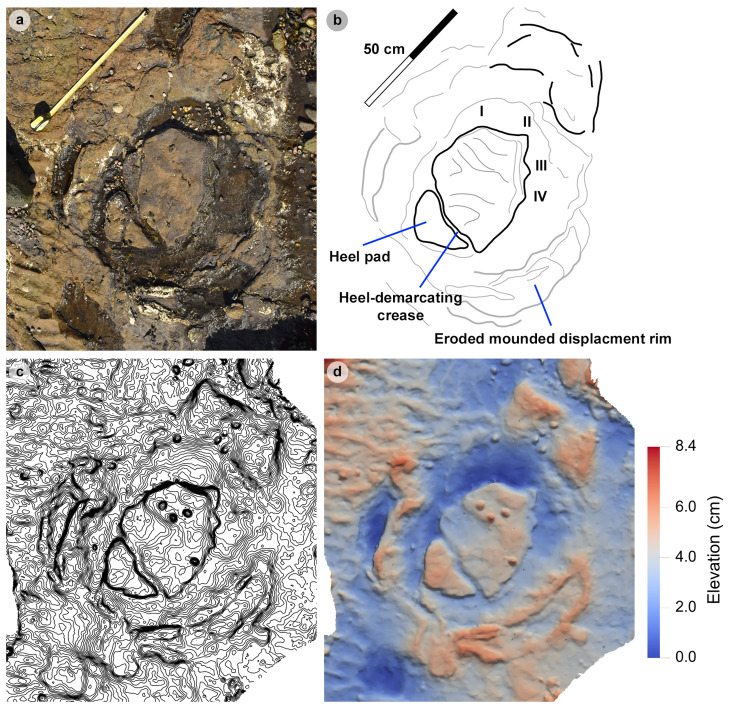
Overview of PC-SA-1-82. (A) Photograph, (B) DEM, (C) contour map, (D) outline of PC-SA-1-82 – the only pes track in sequence with digits preserved. Digits, except digit i, possess anterolaterally oriented, triangular shaped ungual marks. The heel pad and heel-demarcating crease are also preserved. The widest displacement rim widths are lateral to the pes track.

**Fig 23:**
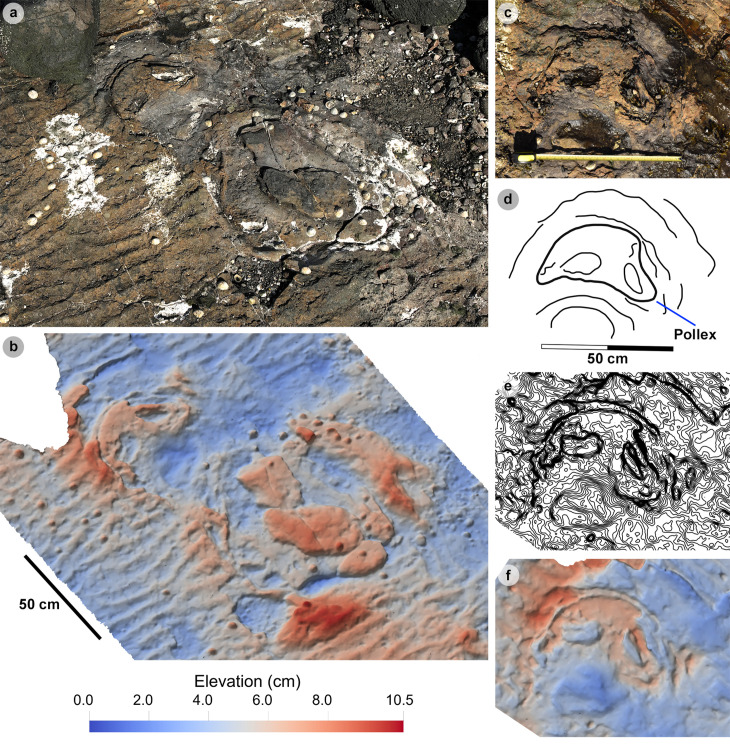
Overview of PC-SA-1-79 and 80. (A) Photograph and (B) DEM showing left manus and pes pair in context. The eroded pes track lacks clear digits and pads. (C) Photograph, (D) outline, (E) contour map, (F) DEM of manus track PC-SA-1-79. The pollex is posterolaterally oriented on the right side of this left manus.

**Fig 24:**
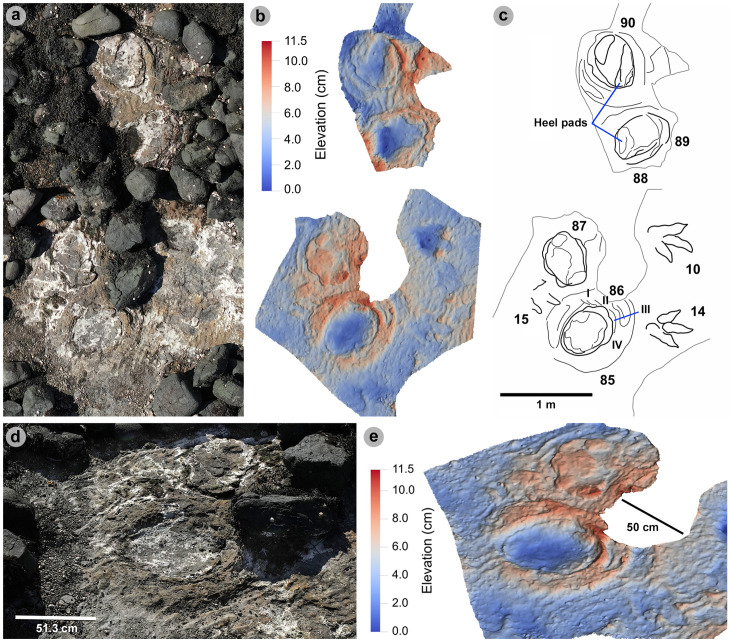
Overview of PC-SA-2. (A) Textured orthophoto, (B) DEM, (C) outline. The right pes tracks in the sequence partially overprint their correspondent manus tracks. PC-TH-2-15 (partially uncovered) partly over-impresses the displacement rim of PC-SA-2-85. PC-TH-1-10 and PC-TH-2-14 can be seen opposite PC-SA-2-87 and 85 respectively. Worn heel pads are visible on PC-SA-2-88 and 90. (D) photograph and (E) DEM of PC-SA-2-85 (foreground) shows a sharply defined mounded displacement rim with ripples. Such a structure is almost absent from PC-SA-2-87 (background).

**Fig 25:**
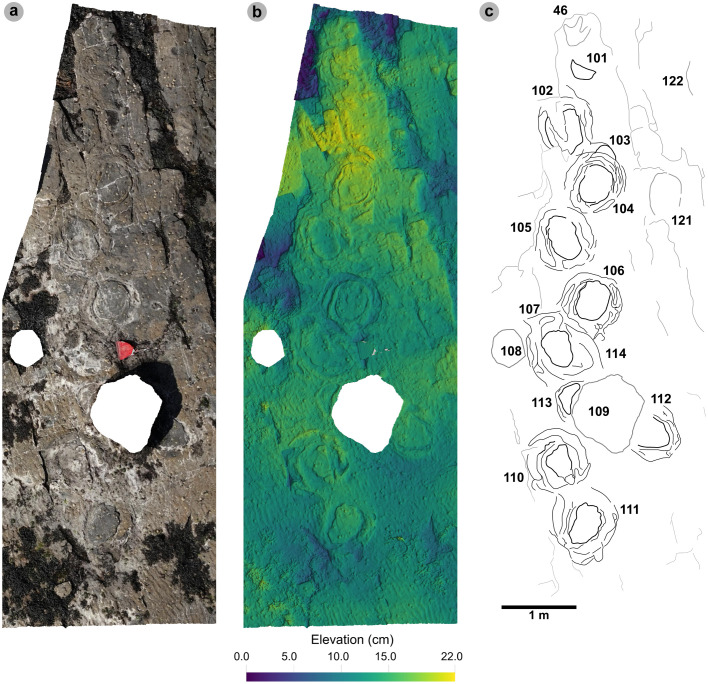
Overview of PC-SA-3. (A) Textured orthophoto, (B) DEM, (C) outline. Most pes tracks lack digits and pad impressions and overprint correspondent manus tracks. Each pes was surrounded by a variably eroded, rippled displacement rim - likely originally mounded as seen on PC-SA-3-111. PC-SA-3-109 is obstructed by a large boulder and was unmeasured. PC-SA-A-1 (PC-SA-A-1-112 to 114) meanwhile intersects PC-SA-3 between PC-SA-3-108 and 110.

**Fig 26:**
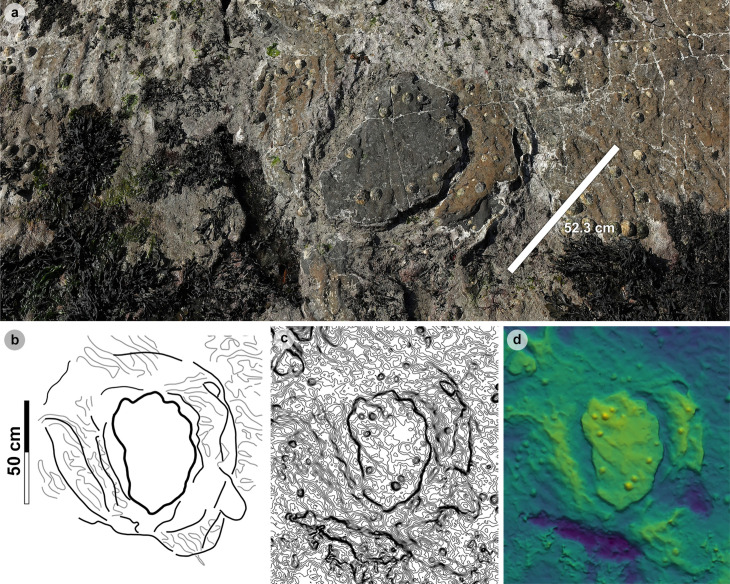
Overview of PC-SA-3-111. (A) Photograph, (B) outline, (C) contour map, (D) DEM. PC-SA-3-111 is the only pes in the trackway with well-defined digits and a mounded displacement rim with ripples across the surface. Note the widest displacements occur laterally to the track.

**Fig 27:**
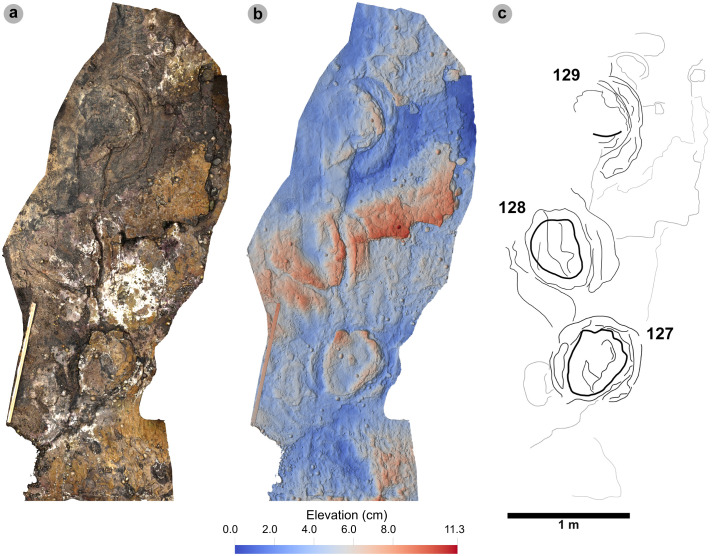
Overview of PC-SA-5. (A) Textured orthophoto, (B) DEM, (C) outline. This pes only trackway is similar to PC-SA-3 in track spacing. However, due to the poor track definition of PC-SA-5-129 and other possible preceding tracks before PC-SA-3-127, trackway measurements could not be recorded. PC-SA-5-127 features four well-defined anterolaterally oriented subtriangular digits – amongst the most clearly defined at the locality.

**Fig 28:**
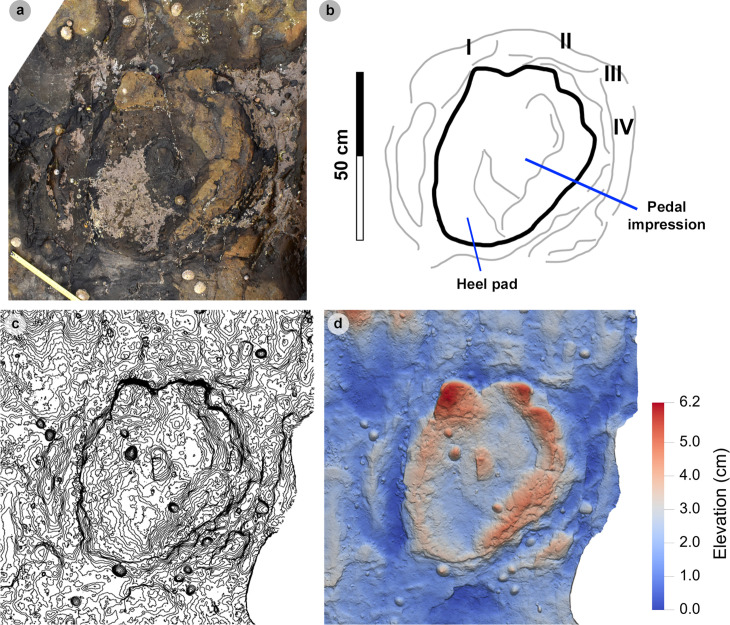
Overview of PC-SA-5-127. (A) Photograph, (B) outline, (C), contour map, (D) DEM. Digit i is the largest digit and anterolaterally oriented to the left. Digits ii-iv gradually decline in length and subtriangular pronouncement and are anterolaterally oriented to the right.

**Table 20:** Track measurements used to characterise morphotype-2.

Specimen	PG	L/ R	Manus or pes	L	W	L/W	IPS	IMS	H	ROT
PC-SA-1-**69**	1	R	Manus	23.20	34.90	0.66	n/a	28.45	n/a	n/a
PC-SA-1-**71**	0.5	L	Manus	18.60	39.50	0.47	n/a	27.11	n/a	n/a
PC-SA-1-**72**	0.5	L	Pes	48.50	39.60	1.22	43.82	n/a	1.62	n/a
PC-SA-1-**73**	0	R	Manus	18.80	38.90	0.48	n/a	27.04	n/a	4.68
PC-SA-1-**75**	1	L	Manus	21.10	39.30	0.54	n/a	28.80	n/a	43.66
PC-SA-1-**76**	0.5	L	Pes	50.50	37.40	1.35	43.46	n/a	1.51	48.50
PC-SA-1-**77**	0.5	R	Manus	20.60	33.60	0.61	n/a	26.31	n/a	14.55
PC-SA-1-**78**	0.5	R	Pes	54.60	40.70	1.34	47.14	n/a	1.79	10.65
PC-SA-1-**79**	1.5	L	Manus	26.20	41.60	0.63	n/a	33.01	n/a	26.80
PC-SA-1-**80**	0.5	L	Pes	53.30	42.40	1.26	47.54	n/a	1.44	18.04
PC-SA-1-**81**	0.5	R	Manus	21.90	38.10	0.57	n/a	28.89	n/a	24.83
PC-SA-1-**82**	1	R	Pes	49.20	36.80	1.34	42.55	n/a	1.47	38.07
PC-SA-1-**83**	1	L	Manus	24.80	41.20	0.60	n/a	31.96	n/a	32.39
PC-SA-1-**84**	0.5	L	Pes	56.90	35.90	1.58	45.20	n/a	1.41	22.03
PC-SA-2-**85**	1	R	Pes	51.30	38.60	1.33	44.50	n/a	1.83	35.68
PC-SA-2-**88**	1	R	Pes	52.40	38.50	1.36	44.92	n/a	1.72	44.06
PC-SA-2-**90**	0.5	L	Pes	55.10	40.70	1.35	47.36	n/a	n/a	44.58
PC-SA-I-**101**	1	L?	Manus	17.00	32.80	0.52	n/a	23.61	n/a	n/a
PC-SA-3-**102**	0.5	L	Pes	56.20	39.00	1.44	46.82	n/a	n/a	17.11
PC-SA-3-**104**	0.5	R	Pes	53.30	37.50	1.42	44.71	n/a	1.95	30.86
PC-SA-3-**105**	0.5	L	Pes	46.70	34.90	1.34	40.37	n/a	n/a	27.03
PC-SA-3-**106**	0.5	R	Pes	49.40	39.70	1.24	44.29	n/a	n/a	34.28
PC-SA-3-**108**	1	L	Pes	46.40	33.90	1.37	39.66	n/a	1.67	35.53
PC-SA-3-**110**	1.0	L	Pes	46.60	36.70	1.27	41.35	n/a	n/a	35.66
PC-SA-3-**111**	1.5	R	Pes	52.30	35.20	1.49	42.91	n/a	n/a	27.24
PC-SA-5-**127**	1.5	R	Pes	55.40	41.20	1.34	47.78	n/a	n/a	n/a
PC-SA-5-**128**	1	L	Pes	51.60	39.00	1.32	44.86	n/a	n/a	n/a
***AVERAGE PES***	***0.78***			***51.65***	***38.21***	***1.35***	***44.10***	n/a	***1.64***	***31.29***
***AVERAGE MANUS***	***0.78***			***21.36***	***37.77***	***0.57***	n/a	***28.35***	n/a	***24.49***

All lengths were measured in cm. Additional measurements included the index of pes size (IPS), index of manus size (IMS), track heteropody (H), and rotation (ROT). Rotation was measured in degrees between the pes long axis and stride length line. All track measurements were rounded to two decimal places.

**Table 21:** Track measurements for PC-SA-1.

Specimen	PG	L/ R	Manus or pes	L	W	L/W	Hip height	IPS	IMS	H
PC-SA-1-**68**	0	L?	Pes	n/a	n/a	n/a	n/a	n/a	n/a	n/a
PC-SA-1-**69**	1	R	Manus	23.20	34.90	0.66	n/a	n/a	28.45	n/a
PC-SA-1-**70**	0	R	Pes	56.30	39.50	1.43	2.25	47.16	n/a	1.66
PC-SA-1-**71**	0.5	L	Manus	18.60	39.50	0.47	n/a	n/a	27.11	n/a
PC-SA-1-**72**	0.5	L	Pes	48.50	39.60	1.22	1.94	43.82	n/a	1.62
PC-SA-1-**73**	0	R	Manus	18.80	38.90	0.48	n/a	n/a	27.04	n/a
PC-SA-1-**74**	0	R	Pes	n/a	n/a	n/a	n/a	n/a	n/a	n/a
PC-SA-1-**75**	1	L	Manus	21.10	39.30	0.54	n/a	n/a	28.80	n/a
PC-SA-1-**76**	0.5	L	Pes	50.50	37.40	1.35	2.02	43.46	n/a	1.51
PC-SA-1-**77**	0.5	R	Manus	20.60	33.60	0.61	n/a	n/a	26.31	n/a
PC-SA-1-**78**	0.5	R	Pes	54.60	40.70	1.34	2.18	47.14	n/a	1.79
PC-SA-1-**79**	1.5	L	Manus	26.20	41.60	0.63	n/a	n/a	33.01	n/a
PC-SA-1-**80**	0.5	L	Pes	53.30	42.40	1.26	2.13	47.54	n/a	1.44
PC-SA-1-**81**	0.5	R	Manus	21.90	38.10	0.57	n/a	n/a	28.89	n/a
PC-SA-1-**82**	1	R	Pes	49.20	36.80	1.34	1.97	42.55	n/a	1.47
PC-SA-1-**83**	1	L	Manus	24.80	41.20	0.60	n/a	n/a	31.96	n/a
PC-SA-1-**84**	0.5	L	Pes	56.90	35.90	1.58	2.28	45.20	n/a	1.41
***AVERAGE PES***	***0.39***			***52.76***	***38.90***	***1.36***	***2.11***	***45.27***	n/a	***1.56***
***AVERAGE MANUS***	***0.75***			***21.90***	***38.39***	***0.57***	n/a	n/a	***28.95***	n/a

Length (l) and width (w) were measured in cm. Hip height was measured in m. Rotation was measured in degrees between the pes long axis and stride length line. All track measurements were rounded to two decimal places.

**Table 22:** PC-SA-1 gauges.

Specimen	SW	OW	TR	Gauge
PC-SA-1-**78**	0.43	1.06	40.23	Medium
PC-SA-1-**80**	0.46	0.92	50.11	Narrow
PC-SA-1-**82**	0.41	0.97	41.83	Medium
***AVERAGE***	***0.43***	***0.99***	***44.05***	***Medium***

The gauge was determined by a trackway ratio (TR). TR was calculated based on side width (SW) and overall width (OW) – measured in metres. Note a gauge could not be determined for the first and last pes in sequence. Gauges for pes tracks after PC-SA-1-78 could not be determined with certainty due to poorly defined track margins.

**Table 23:** PC-SA-1 trackway measurements.

Specimen	P	λ	PRG	γ	ROT	WAP	WAM	WAP /WAM	WAP /PL	WAM /MW	WAP /IPS	WAM /IMS
PC-SA-1-**68**	n/a	n/a	n/a	n/a	n/a	n/a	n/a	n/a	n/a	n/a	n/a	n/a
PC-SA-1-**69**	n/a	n/a	n/a	n/a	n/a	n/a	n/a	n/a	n/a	n/a	n/a	n/a
PC-SA-1-**70**	n/a	n/a	n/a	n/a	n/a	n/a	n/a	n/a	n/a	n/a	n/a	n/a
PC-SA-1-**71**	1.80	n/a	n/a	n/a	n/a	n/a	1.00	n/a	n/a	2.53	n/a	3.69
PC-SA-1-**72**	1.36	n/a	n/a	121.94	n/a	0.67	n/a	0.67	1.39	n/a	1.54	n/a
PC-SA-1-**73**	1.77	2.95	2.77	n/a	4.68	n/a	1.18	n/a	n/a	3.03	n/a	4.36
PC-SA-1-**74**	1.42	2.43	1.24	120.57	n/a	0.80	n/a	0.67	n/a	n/a	n/a	n/a
PC-SA-1-**75**	1.91	2.81	2.55	n/a	43.66	n/a	1.13	n/a	n/a	2.87	n/a	3.92
PC-SA-1-**76**	1.87	2.87	1.69	123.00	48.50	0.71	n/a	0.63	1.40	n/a	1.63	n/a
PC-SA-1-**77**	1.47	2.53	2.26	n/a	14.55	n/a	1.07	n/a	n/a	3.18	n/a	4.07
PC-SA-1-**78**	1.24	2.75	1.02	121.01	10.65	0.67	n/a	0.62	1.22	n/a	1.42	n/a
PC-SA-1-**79**	1.65	2.27	2.00	n/a	26.80	n/a	0.98	n/a	n/a	2.35	n/a	2.96
PC-SA-1-**80**	1.52	2.40	1.36	137.70	18.04	0.50	n/a	0.51	0.93	n/a	1.05	n/a
PC-SA-1-**81**	1.74	2.77	2.59	n/a	24.83	n/a	1.09	n/a	n/a	2.86	n/a	3.78
PC-SA-1-**82**	1.27	2.60	1.17	128.91	38.07	0.56	n/a	0.51	1.14	n/a	1.32	n/a
PC-SA-1-**83**	1.43	2.28	2.01	n/a	32.39	n/a	n/a	n/a	n/a	n/a	n/a	n/a
PC-SA-1-**84**	1.33	2.35	1.21	n/a	22.03	n/a	n/a	n/a	n/a	n/a	n/a	n/a
***AVERAGE PES***	***1.43***	***2.57***	***1.28***	***125.52***	***27.46***	***0.65***	n/a	***0.60***	***1.22***	n/a	***1.39***	n/a
***AVERAGE MANUS***	***1.68***	***2.60***	***2.36***	n/a	***28.45***	n/a	***1.07***	n/a	n/a	***2.81***	n/a	***3.80***

Note PC-SA-3-73 is inwardly rotated – the rest exhibit outward rotation. Pace (P), stride (λ), progression (PRG), width of pes angulation (WAP), width of manus angulation (WAM) were measured in metres. The pace angulation (γ) and track rotation (ROT) were measured in degrees.

**Table 24:** Overall trackway velocity and gait ratio for PC-SA-1.

Track λ	L/ R	L	L-SD	h	λ	λ/h	AX-V	TR-V	AX-V-SD	TR-V-SD
SA-1-72, 70	R	52.40	0.08	2.09	2.43	1.16	1.46	1.32	0.02	0.02
SA-1-76, 72	L	49.50	6.89	2.09	2.87	1.38	1.92	1.74	0.10	0.08
SA-1-78, 76	R	52.55	0.18	2.09	2.81	1.35	1.86	1.68	0.06	0.05
SA-1-80, 78, 76	L	52.80	0.46	2.09	2.40	1.15	1.43	1.29	0.03	0.03
SA-1-82, 80, 78	R	52.37	0.06	2.09	2.60	1.25	1.63	1.47	0.00	0.00
SA-1-84, 82, 80	L	53.13	1.02	2.09	2.35	1.13	1.38	1.25	0.05	0.04
***AVERAGE***		***52.13***	***1.20***	***2.09***	***2.57***	***1.23***	***1.61***	***1.45***	***0.21***	*0.19*

Note the length of PC-SA-1-74 was not recorded due to track incompleteness. Track length (L) was measured in cm. Hip height (h) and stride (λ) were measured in m. Velocity was measured in m/s. AX velocity follows [69], while RT velocity follows [70].

**Table 25:** Trackway measurements for PC-SA-2.

Specimen	PG	L/ R	Manus or pes	L	W	L/W	Hip height	IPS	IMS	ROT
PC-SA-2-**85**	1	R	Pes	51.30	38.60	1.33	2.05	44.50	n/a	35.68
PC-SA-2-**86**	0.5	R	Manus	16.20	36.40	0.45	n/a	n/a	24.28	n/a
PC-SA-2-**87**	0	L	Pes?	54.90	39.80	1.38	2.20	46.74	n/a	32.36
PC-SA-2-**88**	1	R	Pes	52.40	38.50	1.36	2.10	44.92	n/a	44.06
PC-SA-2-**89**	0	R	Manus	18.00	37.90	0.47	n/a	n/a	26.12	n/a
PC-SA-2-**90**	0.5	L	Pes	55.10	40.70	1.35	2.20	47.36	n/a	44.58
***AVERAGE PES***	*0.63*			***53.43***	***39.40***	***1.36***	***2.14***	***45.88***	n/a	***39.17***
***AVERAGE MANUS***	*0.25*			***17.10***	***37.15***	***0.46***	n/a	n/a	***25.20***	n/a

Heteropody was not recorded as manus tracks were overprinted by pes. This additionally led to manus track measurements which did not reflect a true track length. Length (l) and width (w) were measured in cm. Hip height was measured in m. Rotation was measured in degrees between the pes long axis and stride length line. All track measurements were rounded to two decimal places.

**Table 26:** Trackway measurements for PC-SA-3.

Specimen	PG	L/ R	Manus or pes	L	W	L/W	Hip height	IPS	IMS
PC-SA-3-**102**	0.5	L	Pes	56.20	39.00	1.44	2.25	46.82	n/a
PC-SA-3-**103**	0.5	R	Manus	17.50	29.90	0.59	n/a	n/a	22.87
PC-SA-3-**104**	0.5	R	Pes	53.30	37.50	1.42	2.13	44.71	n/a
PC-SA-3-**105**	0.5	L	Pes	46.70	34.90	1.34	1.87	40.37	n/a
PC-SA-3-**106**	0.5	R	Pes	49.40	39.70	1.24	1.98	44.29	n/a
PC-SA-3-**107**	0.5	L	Manus	16.90	33.20	0.51	n/a	n/a	23.69
PC-SA-3-**108**	1	L	Pes	46.40	33.90	1.37	1.86	39.66	n/a
PC-SA-3-**109**	n/a	R	Pes	n/a	n/a	n/a	n/a	n/a	n/a
PC-SA-3-**110**	1	L	Pes	46.60	36.70	1.27	1.86	41.35	n/a
PC-SA-3-**111**	1.5	R	Pes	52.30	35.20	1.49	2.09	42.91	n/a
***AVERAGE PES***	***0.79***			***50.13***	***36.70***	***1.37***	***2.01***	***42.87***	n/a
***AVERAGE MANUS***	***0.50***			***17.20***	***31.55***	***0.55***	n/a	n/a	***23.28***

Heteropody was not recorded as manus tracks were overprinted by pes. This additionally led to manus track measurements which did not reflect a true track length. Length (l) and width (w) were measured in cm. Hip height was measured in m. Rotation was measured in degrees between the pes long axis and stride length line. All track measurements were rounded to two decimal places.

**Table 27:** Trackway and gauge measurements for PC-SA-3.

Specimen	P	λ	PRG	γ	ROT	WAP	WAP /PL	WAP /IPS	SW	OW	TR	Gauge
PC-SA-3-**102**	n/a	n/a	n/a	n/a	17.11	n/a	n/a	n/a	0.43	0.85	50.06	Narrow
PC-SA-3-**103**	n/a	n/a	n/a	n/a	27.06	n/a	n/a	n/a	n/a	n/a	n/a	n/a
PC-SA-3-**104**	0.84	n/a	0.67	117.76	30.86	44.90	0.84	1.00	0.45	0.82	54.62	Narrow
PC-SA-3-**105**	0.91	1.49	0.80	123.55	27.03	42.70	0.91	1.06	0.40	0.80	50.00	Narrow
PC-SA-3-**106**	0.90	1.59	0.78	113.44	34.28	45.70	0.93	1.03	0.43	0.83	51.69	Narrow
PC-SA-3-**107**	n/a	n/a	n/a	n/a	36.17	n/a	n/a	n/a	n/a	n/a	n/a	n/a
PC-SA-3-**108**	0.78	1.40	0.60	n/a	35.53	48.90	1.05	1.23	0.40	0.86	46.16	Medium
PC-SA-3-**109**	0.93	1.40	0.80	n/a	n/a	47.80	n/a	n/a	n/a	n/a	n/a	n/a
PC-SA-3-**110**	0.83	1.48	0.69	n/a	35.66	45.80	0.98	1.11	0.40	0.82	48.72	Medium
PC-SA-3-**111**	0.97	1.55	n/a	n/a	27.24	n/a	n/a	n/a	0.40	0.79	50.00	Narrow
***AVERAGE PES***	***0.88***	***1.48***	***0.72***	***118.25***	***29.67***	***45.97***	***0.94***	***1.09***	***0.41***	***0.82***	***50.15***	Narrow
***AVERAGE MANUS***	n/a	n/a	n/a	n/a	***31.62***	n/a	n/a	n/a	n/a	n/a	n/a	n/a

As manus tracks are mostly absent or overprinted by pes, WAM, WAP/WAM, WAM/MW, and WAM/IMS calculations were omitted. Pace (P), stride (λ), progression (PRG), and width of pes angulation (WAP) were measured in metres. The pace angulation (γ) and track rotation (ROT) were measured in degrees. The gauge was determined by a trackway ratio (TR). TR was calculated based on side width (SW) and overall width (OW) – measured in metres.

**Table 28:** The overall trackway velocity and gait ratio for PC-SA-3.

Track λ	L/ R	L	L-SD	h	λ	λ/h	AX-V	TR-V	AX-V-SD	TR-V-SD
SA-3-105, 104, 102	L	52.07	10.22	1.95	1.49	0.76	0.70	0.63	0.00	0.00
SA-3-106, 105, 104	R	49.80	0.87	1.95	1.59	0.81	0.78	0.70	0.01	0.01
SA-3-108, 106, 105	L	47.50	1.88	1.95	1.40	0.72	0.63	0.57	0.00	0.00
SA-3-108, 106	R	47.90	0.94	1.95	1.40	0.71	0.62	0.56	0.00	0.00
SA-3-110, 108	L	46.50	5.61	1.95	1.48	0.76	0.69	0.62	0.00	0.00
SA-3-111, 110	R	49.45	0.34	1.95	1.55	0.79	0.74	0.67	0.00	0.00
***AVERAGE***		***48.87***	***1.82***	***1.95***	***1.48***	***0.76***	***0.69***	***0.63***	***0.06***	***0.05***

**Table 29:** Measurements of PC-SA-5 tracks.

Specimen	PG	L/ R	Manus or pes	L	W	L/W	Hip height	IPS
PC-SA-5-**127**	1.5	R	Pes	55.40	41.20	1.34	2.22	47.78
PC-SA-5-**128**	1	L	Pes	51.60	39.00	1.32	2.06	44.86
PC-SA-5-**129**	0.5	R	Pes	n/a	n/a	n/a	n/a	n/a
***AVERAGE PES***	***1.00***			***53.50***	***40.10***	***1.33***	***2.14***	***46.32***

Track length (l) and width (w) were measured in cm. Hip height was measured in m. All track measurements were rounded to two decimal places.

**Diagnosis** – Pes tracks are ~ 50 cm long and can feature four digits with ungual marks. Digit i is typically oriented anteromedially or anterolaterally in the opposite direction to subtle anterolateral digits ii-iv. Manus tracks lack non-pollex digits and possess a subtle posterolaterally subtriangular, tapering pollex. Track rotations rarely exceed 40°. Trackways are narrow to medium gauged.

#### Description.

Small-medium sized oval/subtriangular shaped pes tracks between 46.4-56.9 cm in length and 33.9-42.4 cm wide (l/w ratio 1.22-1.58) with an IPS between 39.7-47.8. Tracks are asymmetrical and anteriorly widen from rounded heels. The widest width was measured across the anterior third of the track. Most pes tracks are outwardly rotated from the stride line (10.7°-48.5°). Digits and ungual marks i-iv are subtriangular in shape and rarely observed. The pedal and heel pads are also rarely observed. Each is separated by a shallow ‘s’ shaped heel-demarcating crease when visible.

Manus tracks measured between 17.0-26.2 cm long and 32.8-41.6 cm wide (l/w ratio 0.47-0.66) with an IMS between 23.6-33.0. Tracks are asymmetric, crescentic, and lack digits (except for a posterolaterally tapering pollex). Almost all tracks are outwardly orientated at greater angles than pes equivalents (14.6°-43.7°). On average, outward manus rotations (24.5°) are shallower than pes equivalents (31.3°).

### Morphotype-2 material description

#### PC-SA-1.

Eight visible manus/pes pairs, and an additionally isolated pes, make up a 12.4 m long trackway overall bearing 37.9° against a 226° flow-direction indicated by ripples (Fig 21, Tables 21–24). Due to the south westerly bed dip, bed 1 is gradually exposed toward the present-day high tide mark as bed 2 appears more eroded. As a result, the track margin definition declines (preservation grades =  0-1.5). Tracks are surrounded by large, shallow convex epirelief displacement rims which are variably scoured by present-day erosion. Uneroded displacement rims are shallowly mounded with ripples, while track surfaces are unrippled.

Pes tracks are on average 52.8 cm long by 38.9 cm wide (l/w ratio 1.36), with an IPS of 45.3. Based on track length, the trackmaker may have possessed an estimate hip height of ~ 2.11 m. Tracks are oval/subtriangular in shape and widest subtly anterior of the long axis midpoint. Although most tracks lack digits and pads, four anterolaterally oriented worn digits (i-iv) and faint ungual marks (ii-iv only) are present on PC-SA-1-82 (Fig 22). Although visible, due to erosion, the orientation of digit i could not be determined. The heel pad on PC-SA-1-82 is separated from the pedal impression by a heel-demarcating crease.

Manus tracks are on average 21.9 cm long by 38.4 cm wide with an IMS of 29.0. Though clearly defined, manus tracks (like PC-SA-1-79) lack non-pollex digits and correspondent ungual marks. Most possess a posterolaterally-directed, subtriangular pollex (Fig 23). Paired manus and pes tracks possess an average heteropody ratio of 1:1.56.

Due to poorly defined track margins, the gauge could only be measured between PC-SA-1-82 and 78. Between these three tracks, the gauge is narrow-medium (40.2-50.1%) (Table 22). This contributes to the overall trackway asymmetry. This is further emphasised by the variation of pes and manus track positions. Manus centres can be positioned slightly outside (e.g., PC-SA-1-80 and 84) or inside (e.g., PC-SA-1-78 and 82) of corresponding extended pes long axes. Despite this, manus tracks are predominantly further away from the track midline than pes equivalents (average WAP/WAM ratio =  0.60). Track rotations fluctuate throughout the sequence and are on average subequal between pes (27.5°) and manus tracks (28.5°). Right pes rotations from PC-SA-1-82 decrease, while left equivalents fluctuate and remain consistently outwardly rotated. The PC-SA-1-73 manus is the only recorded inwardly rotated track in the sequence (Table 23).

Left (2.35-2.87 m) and right (2.60-2.75 m) pes strides both increase up to PC-SA-1-74, when the right stride declines to 2.43 m. Paces from left tracks are larger (1.33-1.97 m) than respectively subsequent right equivalents (1.24-1.42 m) in sequence. Likewise, left-right track progressions are larger (1.21-1.69 m) than vice versa (1.02-1.24 m). These asymmetrical patterns may reflect natural fluctuations in the trackmaker path. The average trackway velocity estimates (1.45-1.61 m/s =  5.22-5.80 km/h) and stride/hip height ratio (1.23) overall indicate a walking gait (Table 24).

#### PC-SA-2.

Four pes and two partially overprinted manus tracks make up a 3.4 m long trackway bearing 311.2° (Fig 24, Table 25). Most tracks feature displacement rims. The clearest is mounded and rippled and surrounds PC-SA-2-85 (Fig 25D-E). The concave epirelief right pes tracks are more clearly defined than the shallow convex epirelief left equivalents. Overall, tracks score preservation grades between 0-1.

Pes tracks are on average 53.4 cm long by 39.4 cm wide (l/w ratio 1.36), with an IPS of 45.9. Tracks are ovular and widest subtly anterior of the long axis midpoint. Although most pes tracks lack digits, four worn digits are visible on PC-SA-2-85. PC-SA-2-88 is in concave epirelief and may have been impressed with emphasis on the left side of the foot as the heel pad appears more deeply registered into the sediment than the rest of the track. Although measured, the visible right manus tracks were overprinted by correspondent pes and do not reflect the true track length. Based on other trackways at Prince Charles’s Point, PC-SA-2 could be incomplete as two possible pes tracks may have occupied the large space between PC-SA-2-87 and 88. Due to this, trackway measurements are not taken. The sauropod is estimated to have a ~ 2.14 m hip height.

#### PC-SA-3.

PC-SA-3 consists of eight sequential shallow convex epirelief pes tracks and two manus tracks bearing 330° over 5.2 m (Figs 25, Tables 26–28). Across the sequence, pes tracks gradually become rounded and score lower preservation grades (between 0.5-1.5). Manus tracks were overprinted by the corresponding pes and are not measured (preservation grades =  0.5).

Pes tracks are on average 50.1 cm long by 36.7 cm wide (l/w ratio 1.37) with an IPS of 42.9. Tracks are variably ovular and widest across the anterior third of the long axis. Each is surrounded by large, often scoured displacement rims. The displacement rim surrounding PC-SA-3-111 is one of the most uneroded at the locality and is mounded with clear ripples across the surface. Like other tracks in PC-SA-3, the displacement rim is widest and in most relief laterally to the track. PC-SA-3-111 is also the only pes to feature clearly defined, subtriangular, anterolaterally oriented digits (i-iv) in the trackway (Fig 26). These digits decrease in size toward digit iv like PC-SA-1-82. Manus tracks, meanwhile, are difficult to distinguish due to pes overprinting. As a result, the measurements do not reflect the true track dimensions and heteropody estimation is omitted for this trackway. An estimated hip height of ~ 2.01 m is inferred from pes track length.

The quadrupedal trackway is characterised by a narrow to marginal medium gauge by trackway ratios between 46.2-54.6% (Table 27). These gauges are emphasised by pes tracks mostly overlapping the track midline as WAP/PL ratios range between 0.84-1.05. Left pes track rotations gradually decline across the sequence (35.7°-17.1°) while right equivalents fluctuate (34.3°-27.2°). The 17.1° from PC-SA-3-102 is based on the projection of the PC-SA-3-105 stride. Unlike PC-SA-1, the low track rotation ranges contributed to a minimalised track asymmetry. This is also reflected in the low variation in stride (1.40-1.59 m), pace (0.78-0.97 m), and progression (0.60-0.80 m). Right pes track paces (0.84-0.97 m) and progression (0.67-0.80 m) consistently decline in between fluctuations in left paces (0.78-0.91 m) and progression (0.60-0.80). The estimated average trackmaker velocity estimates (0.63-0.69 m/s =  2.27-2.48 km/h) and stride/hip height ratio (0.76) signify a slow walking gait (Table 28).

PC-SA-3-109 track lengths were omitted due to obstruction by a boulder. Track length (L) was measured in cm. Hip height (h) and stride (λ) were measured in m. Velocity was measured in m/s. AX velocity follows [69], while RT velocity follows [70].

#### PC-SA-5.

Situated on a narrow exposure of bed 2 before the main Section Two track cluster, PC-SA-5 is composed of at least three consecutive pes tracks (Fig 27, Table 29). The trackway spans 2 m bearing 328.4°. Further tracks may be present before PC-SA-5-127. However, these hints of sedimentary features have margins too poorly defined to confidently confirm. Due to the exposure of multiple ripple horizons, track margins become progressively poorly defined and score preservation grades between 0.5-1.5. As a result, trackway measurements were not taken, and a precise horizon of origin could not be determined. Despite this, large partial or complete shallow convex epirelief displacement rims surround each track.

PC-SA-5-127 and 128 feature sufficient margin definition to be measured and described. On average, the tracks measure 53.5 cm long by 40.1 cm wide (l/w ratio 1.33) with an IPS of 46.3. Both tracks are ovular in shape and anteriorly widen. PC-SA-5-127 is the only track to feature four clearly defined subtriangular digits. Digit i is anterolaterally oriented in the opposite direction to digits ii-iv (Fig 28). Tapering ungual marks are most visible on digits i-ii. Faint, shallow convex epirelief heel and pedal impressions are also distinguished. An estimated hip height of ~ 2.14 m was interpolated from track length.

### Morphotype-2 discussion

Morphotype-2 is inferred to have been made by a sauropod trackmaker based on the following synapomorphies: quadrupedal trackways, relatively digitigrade pes and manus posture, four entaxonic, semi-circularly arranged digits with ungual marks on pes tracks, and vertically oriented metacarpals that do not therefore leave impressions but impart an overall crescent shape to a pronated/semi-tubularly arranged manus track [86–90]. In contrast to morphotype-2, quadrupedal tracks made by Middle Jurassic stegosaurs described by [91] were smaller with a tridactyl pes (digit iii is the most pronounced digit) and a narrower crescentic manus with a pronounced pollex. Morphotype-2 pes tracks feature comparable morphologies to a non-neosauropod or basal eusauropod. These include a prominently impressed digit i, sickle-shaped digits ii-iii, and comparatively smaller digit iv [88–90]. Although the digits can taper and are anterolaterally oriented, their relatively low pronation contrasts with the synapomorphic pronounced curvature observed on more derived neosauropod tracks [88].

Additional similarities to eusauropods include: an anteriorly widening pes with a horizontally held and spread-out metatarsus and clearly defined heel impression [87,88,92]. Most eusauropod manus metacarpals form shallowly concave posterior margins rather than the tightly bunched metacarpal colonnades of arched ‘U’ shaped equivalents of derived neosauropods [86,93,94]. This is especially apparent on morphotype-2 manus tracks, which are shallowly crescentic. Like most eusauropods and some basal neosauropods (which included diplodocoids and basal macronarians), the pollex of morphotype-2 manus tracks are the only visible digit with a pronounced ungual mark [86,95]. Large pollex ungual marks were also identified by [37] in sauropod manus tracks from the nearby Duntulm Formation at Cairidh Ghlumaig (Duntulm). The tracks contrast the smaller or absent pollex ungual mark of the titanosauriform manus [96].

Morphotype-2 pes sequences are predominantly narrow-medium gauge with an average trackway ratio of 47.1%. Wider gauge trackways are typically associated with Titanosauriformes – sauropods which may have originated, or at least most thoroughly diversified, in the Late Jurassic [68,89]. Despite its medium gauges, the tracks of PC-SA-1 feature similar dimensions and track morphologies present in narrower gauged trackways at Prince Charles’s Point, i.e., PC-SA-3. This suggests that a likely single, non-neosauropod or basal neosauropod trackmaker species may have been capable of leaving multiple trackway gauges. This variation could be due to individual behaviour or dimorphism [63].

Many of the morphotype-2 morphologies are present in tracks assigned as ‘*Breviparopus*-like/*Parabrontopodus*-like’ as per [37,97]. These characteristics include shallowly crescentic manus tracks with a large pollex ungual mark: similar to tracks identified as *Breviparopus* from the Middle Jurassic of Morocco [98; Fig 9.5]; and *Parabrontopodus* from the Late Jurassic of North America [66]. Undiagnosed manus tracks from Yorkshire described by [99] also featured these morphologies.

Morphotype-2 pes tracks meanwhile occupy the lower ~ 50 cm range of either ichnotaxon [66,100]. The tracks additionally anteriorly widen and feature four anterolaterally oriented digits and ungual marks. Although most pes tracks lack digits, [63] suggested the classification of equivalent tracks in Late Jurassic shallow marine carbonate platform sediments in Switzerland was appropriate as some diagnosed *Breviparopus* or *Parabrontopodus* trackways featured tracks without digits. Like the type *Breviparopus* and *Parabrontopodus* trackways, morphotype-2 equivalents are predominantly narrow-medium gauged, although are characterised by a lower average heteropody. Overall, morphotype-2 most resembles equivalent Middle Jurassic *Breviparopus*/*Parabrontopodus*-like morphotypes, such as Ai-Aii from the Cleveland Basin, Yorkshire, England [99,101].

## Discussion

### Summary

Prince Charles’s Point provides evidence for a dinosaur assemblage, which includes large theropods and sauropods, that likely traversed across a locally, shallowly submerged lagoon margin of variable substrate composition in the Late Bathonian on Skye. A further undetermined quadrupedal trackmaker is represented separately in bed 5 and evidences the occurrence of dinosaurs across multiple beds. The overall variation in the preservation of tracks within trackways also indicates variable substrate conditions despite present-day erosion and burial soon after impression. The slow gaits of both theropod and sauropod trackmakers and variability in the preferred direction of travel further suggests cumulative milling behaviour [102].

### Assemblage comparison

At Prince Charles’s Point, we recorded 63 large theropod pes tracks over 56 sauropod manus and pes – in this case representing a greater abundance of large theropod trackmakers. Middle Jurassic aged theropod-dominated assemblages such as this are rare across much of Europe and North America [32,34,35,38,76,96,103,104]. A previously described assemblage in the Kilmaluag Formation at Lùb Score contrasts with an abundance of tiny-medium sized (<30 cm long) theropod tracks [35]. At Prince Charles’s Point, only two medium-sized tracks are known (PC-TH-A-3-47 and 48). Given the short duration of substrate exposure, the absence of tiny-medium sized tracks and dominance of large theropods and sauropods could reflect a census of the most locally abundant dinosaurs [105]. Although both localities are considered lagoon margins, the hypothesised occurrence of post-hatchling care at Lùb Score by [35], may reflect closer proximity to more suitable habitat for such behaviour and the inhabitation of smaller theropods than at Prince Charles’s Point. The assemblage composition thus may have been influenced by the idiosyncratic needs of each clade that was present.

The brackish lagoonal, rippled sandstones of the Duntulm Formation at An Corran meanwhile have thus far mostly yielded large theropod tracks [34]. Morphologically, like morphotype-1a (and European and North American examples of *Megalosauripus*), the tracks possess broad, parallel-sided digits with a ‘2:3:4’ phalangeal pad formula. Metrically, the average length (44.2 cm) and digit ii-iv divarication angles (52°) overlap with the ranges of morphotype-1a. The shorter trackways also reached similar pace angulations up to 180°. A single small sized tridactyl track was further recorded from An Corran [101]. Like Prince Charles’s Point, the track-bearing surface was likely shallowly submerged as it is rippled and lacks desiccation and radial cracks. In the same formation, a single large theropod track is recorded from the sauropod-dominated assemblage at Cairidh Ghlumaig – composed of similar ‘*Breviparopus*-*Parabrontopodus*-like’ tracks to Prince Charles’s Point [37,106; Fig 27]. In the Lealt Shale Formation of Rubha nam Bràithrean (BP2), several poorly preserved large theropod tracks with parallel sided digit margins and weak l/w ratios like morphotype-1a and *Megalosauripus* constitute approximately 10% of the tracks [38]. These are recorded in the same horizon of brackish lagoonal sediment as more abundant sauropod tracks [38]. Similar theropod tracks are also recorded in horizons of Lealt Shale Formation at BP1 and BP3 which represent periods of subaerial exposure [32].

When compared to Rubha nam Bràithrean (BP2) [38] and Cairidh Ghlumaig [37], the balance of trackmakers at Prince Charles’s Point is strikingly different with a greater abundance of theropod tracks over sauropods. The track-bearing horizons across the three localities reflect lagoonal settings and provide little indication of subaerial exposure. However, there appears to be salinity differences between these lagoonal settings, possibly due to their location within the broader and dynamic coastal palaeoenvironment [45]. At Prince Charles’s Point, the lagoonal palaeoenvironment within the Kilmaluag Formation was influenced by freshwater sources [6,40], while the Lealt Shale and Duntulm Formations at BP2 and Cairidh Ghlumaig evidence more brackish/marine influences [6,107,108]. Speculatively, the difference in trackmaker balance between Prince Charles’s Point and other localities with *in-situ* sauropod tracks might arise from the substrate or palaeoenvironmental preferences of different dinosaur clades. Despite these differences in trackmaker assemblage, the takeaways of other Scottish *in-situ* localities remain consistent: sauropods frequently spent time in lagoonal palaeoenvironments along with large-bodied theropods [37–38].

In contrast, it is unclear whether the absence of ornithopods and thyreophorans at Prince Charles’s Point reflects genuine absence or preservation bias. Their presence in the subaerially exposed mudflat sediments of Rubha nam Bràithrean (BP3), described by [32], may suggest that these groups favoured these environments more than shoreline lagoons, although we must be mindful of interpreting such small numbers of tracks.

Elsewhere, similar theropod and sauropod morphotypes, Bxviii and Ai-Aiii respectively, are reported from *ex-situ* blocks of carbonate mudflat sediment in the Cleveland basin [75,99,109]. These bear similar morphologies to the Prince Charles’s Point tracks (see morphotype ichnotaxonomy sections) and are respectively classified as *Megalosauripus* isp. and *Breviparopus* isp. [101]. Despite their presence, smaller theropod and ornithopod morphotypes are recorded in greater abundance along much of the north Yorkshire coast [109]. Bathonian aged sediments in Ardley, Oxfordshire, UK, preserve multiple extensive theropod and sauropod trackways across an emergent/shallowly submerged, carbonate mudflat surface [96]. Although identified as *Megalosauripus*, the tracks are > 50 cm in length (larger than morphotype-1). The sauropod tracks are also much larger and are referred to Titanosauriforms – currently not known from the Inner Hebrides. In Portugal, similarly aged, sized, divaricated, and padded *Megalosauripus* tracks are found within bimodially oriented trackways on a tidal mudflat surface [76]. Based on this comparison, the dinosaur tracks at Prince Charles’ Point are generally consistent with the broader global context. These fit into a bigger global story that appears to show a preference for both sauropods and megalosaurid theropods in more marginal, coastal environments on the basis of their footprints. This pattern could either be due to the higher preservation potential of these environments or genuine trackmaker preferences.

### Trackmaker behaviour

**Fig 29:**
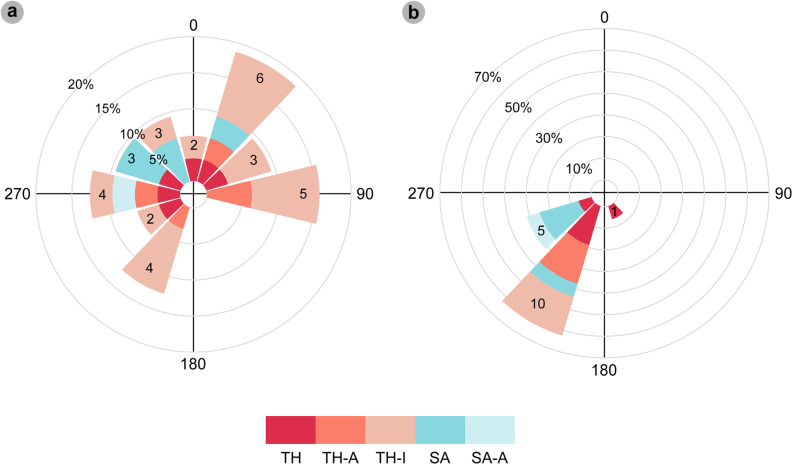
Track and flow-direction (ripple) bearings from magnetic north. (A) Trackmaker and (B) ripple orientations. Classifications include: theropod trackways (TH), theropod track associations (TH-A) isolated theropod tracks (TH-I), sauropod trackways (SA), and sauropod track associations (SA-A). Ripple orientations were measured opposite respective tracks when present. Both theropod and sauropod trackmakers generally show no favoured direction.

The lagoonal margin at Prince Charles’s Point hosted several theropod and sauropod individuals, as evidenced by their tracks and trackways. All bear multiple directions (Fig 29A, Table 30) and lack evidence of gregarious behaviour, i.e., parallel, or structurally spaced trackways [110–112]. Such wide directional variation, coupled with walking gaits, is often attributed to milling behaviour [102]. As evidenced by trackways such as PC-TH-1 and 2, coupled with relatively sharp track definition and lack of invertebrate bioturbation, this behaviour appears time averaged over a short period prior to burial. Similar time averaged milling behaviour has also been observed at other Great Estuarine Group tracksites [32,37,38].

**Table 30:** Track and flow-direction (ripple) bearings.

Specimen	Track orientation (TO)	TO Class	Ripple orientation (RO)	RO Class
PC-TH-1	47.46	45-75	219.29	195-225
PC-TH-2	32.24	15-45	219.04	195-225
PC-TH-3	311.14	285-315	139.21	135-165
PC-TH-4	237.02	225-255	228.22	225-255
PC-TH-5	7.24	345-15	215.83	195-225
PC-TH-6	279.61	255-285	n/a	n/a
PC-TH-A-1	201.71	195-225	213.36	195-225
PC-TH-A-2	87.22	75-105	n/a	n/a
PC-TH-A-3	87.73	75-105	210.56	195-225
PC-TH-A-4	280.2	255-285	221.55	195-225
PC-TH-A-5	42.09	15-45	n/a	n/a
PC-SA-1	37.86	15-45	225.98	225-255
PC-SA-2	311.17	285-315	226.88	225-255
PC-SA-3	330.01	315-345	222.23	195-225
PC-SA-4	314.67	285-315	227.74	225-255
PC-SA-5	328.41	315-345	n/a	n/a
PC-SA-A-1	270	255-285	230.83	225-255
PC-TH-I-01	210	195-225	214.96	195-225
PC-TH-I-02	219	195-225	n/a	n/a
PC-TH-I-16	42	15-45	n/a	n/a
PC-TH-I-41	282	255-285	n/a	n/a
PC-TH-I-46	345	315-345	n/a	n/a
PC-TH-I-53	90	75-105	n/a	n/a
PC-TH-I-54	62.39	45-75	n/a	n/a
PC-TH-I-55	231	225-255	n/a	n/a
PC-TH-I-56	5	345-15	221.36	195-225
PC-TH-I-57	50	45-75	n/a	n/a
PC-TH-I-58	104	75-105	n/a	n/a
PC-TH-I-59	27	15-45	215.31	195-225
PC-TH-I-62	39	15-45	n/a	n/a
PC-TH-I-63	225	195-225	n/a	n/a
PC-TH-I-64	98	75-105	n/a	n/a

The variety of bearings of multiple tracks suggests that trackmakers were not necessarily restricted by local topography, i.e., shoreline positions, or influenced by the direction of palaeocurrents. The ‘TO’ and ‘RO’ classes denote the angular range occupied in the wind rose diagrams.

Such behaviour furthermore was generally independent to the predominant NE to SW flow direction indicated by ripples (Fig 29B, Table 30). In the track-bearing horizons of beds 2 and 5, the ripple orientations opposite tracks predominantly indicated south westerly flow directions (210.5°-230.8°). Occasional southerly (~179°) and south easterly (~143°-159°) flow directed horizons generally lacked tracks. This implies that trackmakers were not constrained by local topography, i.e., shoreline position [111,113]. Despite this, it is noteworthy that no tracks bearing directly south or in southeasterly directions have been recorded. Interestingly, horizons with large ripple wavelengths that lack tracks feature southeasterly flow-directed ripples. A deeper water source could have existed to the southeast of the tracksite. While it is possible to speculate why these trackmakers used the lagoon, such as for food or shelter, there is currently no evidence to support specific usages.

### Interpretation of sauropod tracks

The unusual shallow relief of the Prince Charles’s Point sauropod tracks have led previous geologists observing these structures to hypothesise various non-track explanations for their size and shape. Andrews first recognised the tracks as ‘circular obstacle marks’ within his locality sediment log [40]. Obstacle marks are flow-induced structures which form on sediment surfaces when current eddies become trapped and cause the substrate surface to be scoured around or ahead of an anchored object [114–115]. Andrews later amended this hypothesis and stated that the low energy conditions present in the lagoon would unlikely instigate “turbulent eddy scour-pool excavations” under any large boulder [39; pp. 249].

Andrews then proceeded to provide an extended description of the ‘circular impressions’ and described them as: “platelike structures about 50 cm in diameter, which are found on wave-rippled sandstone” [39; pp. 249]. Andrews suggested four alternative formation mechanisms: scour marks induced by plant remains, jelly fish traces, animal footprints, or fish resting/burrow traces. Andrews considered fish resting/burrow traces as the most likely explanation, as he described them as most comparable in structure and shape to modern mudskipper burrows described by [116]. These burrows were reported to be 20-45 cm in diameters with narrow embankments between 5-10 cm. Andrews, however, noted the absence of mudskippers until the Oligocene and suggested an unknown fish species was responsible [39]. Known burrowing freshwater/low salinity fish, such as lungfish, produce long tubular and ovular subsurface concavities [39,117]. Although circular in cross-section, the widest lungfish burrow diameters are typically < 15 cm [118–119].

Andrews rejected an ‘animal footprint’ interpretation, stating: “the shallow, regular shape of these circular structures is not consistent with the footprints of a heavy animal; neither is there any predictable sequence of depressions, which might be expected in a trackway” [39; pp. 249]. Andrews also commented that “nine of these structures were seen within an area of about 10 m^2^ (the limit of bedding plane exposure)”. Based on this description, [39] most likely described the sedimentary structures we describe as the PC-SA-3 trackway (Fig 25).

Contrary to Andrews’s observations, PC-SA-3 and other diagnosed sauropod trackways are composed of a continuous alternating left-right sequences of oval-subtriangular shaped, anteriorly widening, asymmetric pes tracks – some with digits. The asymmetry is also consistent with a single direction of movement. The lack of un-overprinted elliptical/crescentic manus tracks with a pollex in PC-SA-3, as observed in other trackways (e.g., PC-SA-1), may have contributed to the initial diagnosis. The track figured in [39; Fig 2] may presently suffer from erosion but could feature digits (S10 Fig). All these details are more apparent to us than they were to Andrews because our site drone imagery allows the spatial relationships of the tracks to be viewed from an aerial perspective.

Unlike previously recorded sauropod tracks in the Great Estuarine Group, the Prince Charles’s Point sauropod tracks are shallower with wide displacement rims and are in convex epirelief. Similar tracks have been described in the lagoonal/sabkha Middle Jurassic Iouaridène Formation of Morocco [98] and lagoonal Lower Cretaceous Broome Sandstone [120–121]. [120; pp. 5] attributed similar track morphology to “the physical properties of the substrate and the size and behaviour of the trackmaker.” Sediment properties included grain size, moisture content, viscosity, and sediment disturbance [122–123]. Each property determined the extent of morphological retention based on the duration of exposure before burial [124].

**Fig 30:**
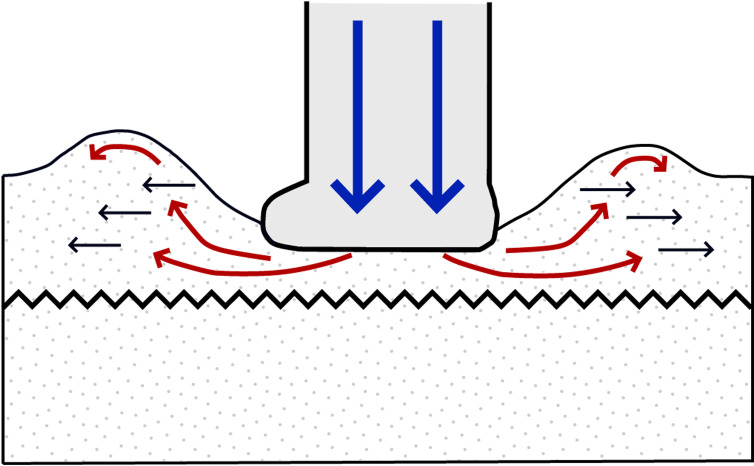
The hypothesised distribution of forces during track impression. Blue arrows represent the downward forces exerted by the sauropod’s limb. Red arrows show the direction of displacement. Due to the desiccated (hardened) bed 1-2 boundary horizon and thinness of compliant bed 2 substrate, the horizontal component of sediment displacement was emphasised (black arrows).

Based on our observations, we can hypothesise how the tracks at Prince Charles’s Point formed in their particular environmental setting. The ~ 10 cm thick (but variable, and sometimes thicker) rippled sandstone which our sauropod tracks occur in are underlain by a hardened, desiccated surface (the bed 1-2 boundary horizon). Due to this, as hypothesised by [120; pp. 6], the downward forces of impressing a track would proximally displace and ripple the sediment outwards, with greater emphasis on a horizontal component, between and during the touch down and weight bearing phases of track impression (Fig 30). This horizontal component would be reinforced by reflected forces from the underlying hardened horizons. A shallow submersion component would emphasise this process and enable sediment to be displaced over a larger distance as no air gaps were present to resist. Eventually, the sediment would gradually displace upwards to form wide, shallowly mounded, consolidated displacement rims as seen on tracks such as PC-SA-2-85 and PC-SA-3-111 (Figs 24, 26). The widest widths of each generally occur laterally around the tracks, which suggests the autopodia were planted almost perpendicular to the substrate. The presence of mounded displacement rims further suggests that at the time of impression, the track-bearing surface was relatively firm *sensu* [122,125]. The sediment, however, was soft enough to be reworked as evidenced by subsequent ripples forming across uneroded mounded displacement rim surfaces.

**Fig 31:**
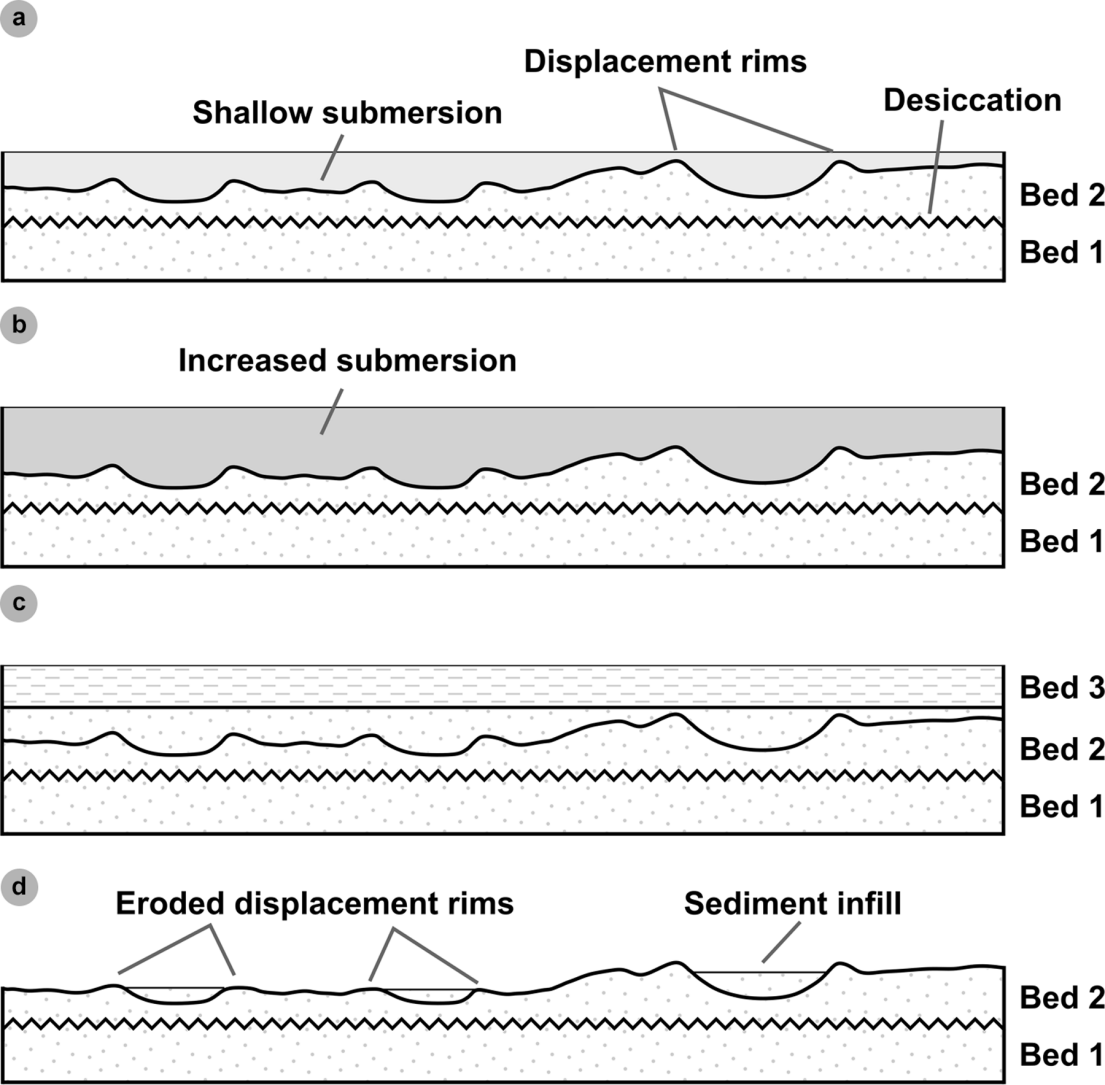
Cross-section demonstrates the hypothesised formation of shallow sauropod tracks. (A) Tracks were impressed into a thin layer of fine-grained sand with variable moisture content. (B) The lagoonal water table increased and enabled further sediment to be quickly deposited on top of the track-bearing layer. (C) The fine-grained sand was overlaid by finer sediment deposited when water levels continued to increase. This would go on to become the bed 3 shale layer. (D) Present-day exposure of the sauropod tracks, some with eroded displacement rims and uneven sediment infill.

The resultant track formed as a shallow, concave impression with variable morphological retention. Based on track infills in the same relatively uneroded horizon viewed in the present-day, most are rounded, featureless impressions which could appear larger than the actual trackmaker autopodium. The morphological variation of tracks within individual trackways may indicate localised variation in sediment moisture content prior to burial *sensu* [58,120], especially with the presence of morphologies such as digits and pads in some tracks and not others. An absence of radial and desiccation cracks within or around the tracks suggests the lagoon margin surface at this time was constantly moist [46]. Based on this, the sauropods likely traversed a locally shallowly submerged lagoon margin surface with variable moisture content (Fig 31).

After impression, the tracks would have become increasingly submerged by the shallow lagoonal body of water, indicated by ~ 5-8 cm long amplitude flow-directed ripples on the track-bearing surface (and displacement rims), and did not suffer uniform morphological degradation, i.e., a few tracks featured digits and pads while most did not. This implies that weak lagoonal currents alone could not have necessarily erased all track morphologies in the time between track impression and burial. This time was likely brief as the track-bearing surface lacked invertebrate bioturbation. Presently, the tracks appear infilled with sediment from an overlying layer, almost level with the surrounding track-bearing layer. Although raised, most displacement rims are affected by present-day erosion and are rarely seen as mounded structures. Due to their fine-grained composition, the sediments may have eroded at a similar rate to induce their unusually shallow appearance.

### Possible trackmaker identification

Great Estuarine Group theropod and sauropod body fossils are scarce and often limited to isolated bones. Theropod bones include a middle caudal vertebra and several teeth [7,26–28]. For sauropods, several vertebra and teeth, and a humerus are known [23–25]. The humerus, previously identified as a femur, was affiliated with a cetiosaurid-type sauropod [23]. A similar scarcity of dinosaur body fossils has been observed across most European Middle Jurassic sediments [5]. Identifications based on these bones have been limited to vague higher-level taxa which may or may not necessarily correspond to the identity of the Prince Charles’s Point trackmakers.

[76] suggested various possible trackmaker identities for their theropod tracks based on the trackmaker body proportions of appropriately spatiotemporally distributed large Middle Jurassic theropods. We used a similar approach due to a lack of comparable skeletal autopodia – typically lost during fossilisation [93].

For morphotype-1a, the relatively moderate average l/w ratios (1.43), 40°-55° digit ii-iv divarication angles, and presence of narrow, elongated ungual marks and padded, parallel sided, broad digits indicate a megalosaurid type theropod diagnosis *sensu* [75]. Based on the average morphotype-1a track length (46.3 cm), trackmakers possessed a ~ 1.85 m hip height. [126] suggested < 50 cm long tracks, represented by the Late Jurassic *Megalosauripus transjuranicus*, did not reflect trackmakers with body lengths < 5 m or > 7 m. We adopt this conclusion and suggest the following potential trackmakers. *Megalosaurus bucklandii* possessed a ~ 6 m total body length and was spatiotemporally widespread across what is now England during the Bathonian [127]. *Duriavenator hesperis*, also recorded in England, was similarly sized at ~ 7 m [128]. Both species were considered capable of registering < 50 cm long tracks with the morphologies of morphotype-1a and *Megalosauripus isp*. [75–76]. The specimens representing *Magnosaurus nethercombensis* (which possessed a ~ 1 m hip height [129]) and *Cruxicheiros newmanorum* (which likely produced larger > 50 cm tracks [130]), are subadults of uncertain ontogenetic status and may have grown to larger body sizes to become appropriate trackmakers. Regardless of potential species, megalosaurids are the only known theropod trackmakers which could impress large tridactyl footprints in the Late Bathonian of modern-day Britain [76,129].

We reach a similar conclusion for morphotype-1b-d. Morphotype-1d could have represented a juvenile megalosaurid, perhaps the same species as represented by morphotype-1a due to similarity in average metrics such as l/w ratios (1.46) and digit ii-iv divarication (56°). Alternatively, morphotype-1d could have represented an entirely different species with a ~ 1 m hip height. Possible English Middle Jurassic theropod trackmakers include *Eustreptospondylus oxoniensis* [131].

For morphotype-2, based on track characteristics, the known Skye body fossil record, and contemporary English taxa, we suggest a non-neosauropod or basal neosauropod species was likely present at Prince Charles’s Point, consistent with the interpretations of [37] for the Duntulm Formation sauropod tracks. On Skye, at least two sauropod species were present based on the differences in morphology between two teeth described by [24] and [25]. The best known sauropod postcranial element from Skye, an isolated 90 cm long near complete left humerus from the Valtos Sandstone Formation, was described as similar to the equivalent bone of the type cetiosaurid species, the Middle Jurassic *Cetiosaurus oxoniensis*, by [23]. Based on the average 51.7 cm pedal track length of morphotype-2, the Valtos humerus would likely proportionally fit within the estimated average trackmaker hip height of ~ 2.07 m. Determining possible affinities with wider English/European skeletal material is problematic due to the relative incompleteness of available autopodia. *Cetiosaurus oxoniensis* was widespread across England with a total body length up to 16 m, but its autopodia are only known from a handful of metatarsals and metacarpals, making it difficult to match the foot to tracks [132]. Other potential eusauropod trackmakers included the similarly aged *Cardiodon* [133]. However, trackmaker identification based on *Cardiodon* is highly uncertain as the species was erected based on a handful of teeth [133–134]. Currently, we consider the most plausible trackmaker to be *Cetiosaurus* or a similar basal eusauropod or potentially a basal neosauropod.

## Conclusion

In the Kilmaluag Formation at Prince Charles’s Point, on the Isle of Skye in Scotland, we report and document 131 dinosaur tracks of Middle Jurassic age. These include tracks we hypothesised were made by a large megalosaurid and a non-neosauropod or basal neosauropod trackmaker. All traversed across a shallowly submerged lagoonal margin surface within a closed, ephemeral, freshwater (low salinity) lagoon palaeoenvironment, represented by a fine-grained rippled sandstone. The trackmakers consistently walked in non-uniform directions, which likely represented cumulative milling behaviour rather than interspecies or gregarious interactions. The occurrence of these tracks in a lagoonal shoreline provides further evidence to the widespread habitation of sauropods in these palaeoenvironments on ancient Skye, as indicated by previous discoveries, and suggests that theropods may have been more common components of the lagoonal assemblages than previously recognised.

## Supporting information

S1 Appendix Further tracksite details including field observations and further tracks.
The appendix is formatted in a.pdf file and contains descriptions, figure captions, and tables for further tracks present at Prince Charles’s Point (including referred morphotype track associations) and additional information recorded in the field. Track photogrammetric models and photo sets are available to download via Dryad: https://doi.org/10.5061/dryad.wh70rxwwx.
(DOCX)

S1 Fig Overview of PC-TH-6.
(A) Textured orthophoto with software-based shadowing, (B) DEM, (C) outline highlights the trackway. The tracks exhibit mixed preservation: PC-TH-6-43 is a concave epirelief track with poorly defined margins, while PC-TH-6-44 and 45 are in convex epirelief.
(TIF)

S2 Fig Photographic and digital representations of selected PC-TH-6 tracks.
Photographs, outlines, contour maps, and DEMs are respectively represented from left to right. (A-D) Although the digit margins of PC-TH-6-44 are indistinct, unlike PC-TH-6-45, the anterior most phalangeal pads are most visible on digits iii-iv. (E-H) PC-TH-6-45 features poor digit margin definition and missing diagnostic morphologies such as phalangeal pads. The original surrounding substrate has been mostly eroded. (I) Selected tracks, indicated by red boxes, in context to the rest of the trackway.
(TIF)

S3 Fig Overview of PC-TH-A-1.
(A) Textured orthophoto with software-based shadowing, (B) DEM, (C) outline. Most tracks were marginally worn and partly infilled by ripples, particularly PC-TH-A-1-04 and 05.
(TIF)

S4 Fig Overview of PC-TH-A-2.
(A) Photograph cropped to model area, (B) DEM, (C) outline. The tracks are in shallow convex epirelief and appear on top of bed 1 as the original surrounding original horizon was eroded.
(TIF)

S5 Fig Overview of PC-TH-A-2-18.
(A) Photograph and (B) outline of PC-TH-A-2-18, which is missing most of its heel. Unlike most morphotype-1a tracks, such as those of PC-TH-1 and 2, PC-TH-A-2-18 exhibits distinct sigmoidal curvature on digit iii.
(TIF)

S6 Fig Overview of PC-TH-A-4.
(A) Photograph cropped to model, (B) DEM, (C) outline. The tracks are almost perpendicular to the long axis of the ripples.
(TIF)

S7 Fig Overview of PC-TH-A-5.
(A) Textured orthophoto with software-based shadowing, (B) DEM, (C) outline. The tracks have low mesaxony like morphotype-1a but overall have poorly defined margins.
(TIF)

S8 Fig Close up photographic and digital representations of PC-TH-A-5 tracks.
Photographs, outlines, contour maps, and DEMs are respectively represented from left to right. (A-D) PC-TH-A-5-60, (E-H) PC-TH-A-5-61. The tracks are in concave epirelief, with partial sediment infill on digits, and are heavily worn.
(TIF)

S9 Fig Overview of PC-TH-3. (A) Textured orthophoto with software-based shadowing, (B) DEM, (C) outline. Most tracks are incomplete or unexposed. PC-TH-3-21 was recognised from its heel, which resembles that of PC-TH-3-20.
(TIF)

S10 Fig Photograph and outlines of PC-SA-I-124.
(A) Photograph highlights a worn displacement rim around the track. (B) Outline highlights possible digits around the top of the track. The ripples which intersected the track have likely since eroded. Presently, a few of these ripples are faintly recognised.
(TIF)
