# Supplementary material for: A new Middle Jurassic lagoon margin assemblage of theropod and sauropod dinosaur trackways from the Isle of Skye, Scotland
Source: PLoS One. 2025 Apr 2;20(4):e0319862. doi: 10.1371/journal.pone.0319862 (PMC11964282; doi:10.1371/journal.pone.0319862)
Supplement: S1 Appendix — The appendix is formatted in a.pdf file and contains descriptions, figure captions, and tables for further tracks present at Prince Charles’s Point (including referred morphotype track associations) and additional information recorded in the field. Track photogrammetric models and photo sets are available to download via Dryad: https://doi.org/10.5061/dryad.wh70rxwwx. (DOCX) [file pone.0319862.s001.docx]

**Supporting information for:**

**“A new Middle Jurassic lagoon margin assemblage of theropod and sauropod dinosaur trackways from the Isle of Skye, Scotland”**

Tone Blakesley^1*^, Paige E. dePolo^2^, Thomas J. Wade^1^, Dugald A. Ross^3^, Stephen L. Brusatte^1^

^1^ School of GeoSciences, University of Edinburgh, Edinburgh, Scotland, United Kingdom

^2^ School of Biological and Environmental Sciences, Liverpool John Moores University, Liverpool, England, United Kingdom

^3^ Staffin Museum, Staffin, Isle of Skye, Scotland, United Kingdom

*Corresponding author:

E-mail: tone.blakesley.research@gmail.com

**S1 Appendix**

**Additional track descriptions**

This section contains the descriptions and figures of additional tracks present at Prince Charles’s Point. The material primarily includes track associations and isolated tracks.

**Morphotype-1a:**

**PC-TH-6**

PC-TH-6 features three large, tridactyl tracks which extend over 2.8 m at 279.6° (S1-S2 Figs, S1-S3 Tables). Due to sediment infiltration and present-day erosion, some tracks lacked clearly defined digits and score preservation grades between 0.5-1 (average 0.67). Despite this, PC-TH-6-44 features sharp ungual marks, and at least two phalangeal pads on digit iii which suggests a theropod trackmaker affinity. The average track length (46.0 cm), and digit ii-iv divarication angles of 48.6° of PC-TH-6-43 and 57.1° of PC-TH-6-44 are furthermore within the range of morphotype-1a (S1 Table). PC-TH-6-44 features the clearest digit margins and possesses a digit iii/ii ratio >1 and digit iii/iv ratio <1 – consistent with most morphotype-1a tracks.

**S1 Fig. Overview of PC-TH-6.** (A) Textured orthophoto with software-based shadowing, (B) DEM, (C) outline highlights the trackway. The tracks exhibit mixed preservation: PC-TH-6-43 is a concave epirelief track with poorly defined margins, while PC-TH-6-44 and 45 are in convex epirelief.

**S2 Fig. Photographic and digital representations of selected PC-TH-6 tracks.** Photographs, outlines, contour maps, and DEMs are respectively represented from left to right. (A-D) Although the digit margins of PC-TH-6-44 are indistinct, unlike PC-TH-6-45, the anterior most phalangeal pads are most visible on digits iii-iv. (E-H) PC-TH-6-45 features poor digit margin definition and missing diagnostic morphologies such as phalangeal pads. The original surrounding substrate has been mostly eroded. (I) Selected tracks, indicated by red boxes, in context to the rest of the trackway.

**S1 Table. Measurements of PC-TH-6 tracks.**

|  |  |  |  | |  | **Digit length (DL)** | | | **DL ratios** | | **Divarication angles** | | |  |  |
| --- | --- | --- | --- | --- | --- | --- | --- | --- | --- | --- | --- | --- | --- | --- | --- |
| **Specimen** | **PG** | **L / R** | **L** | **W** | **L/W** | **II** | **III** | **IV** | **III/II** | **III/IV** | **II-III** | **III-IV** | **II-IV** | **te** | **M** |
| PC-TH-6-**43** | 0.5 | L | 44.40 | 31.30 | 1.42 | 23.80 | 36.90 | 30.90 | 1.55 | 1.19 | 17.51 | 31.08 | 48.59 | 17.80 | 0.57 |
| PC-TH-6-**44** | 1 | R | 49.30 | 35.00 | 1.41 | 26.10 | 33.20 | 34.50 | 1.27 | 0.96 | 21.66 | 35.40 | 57.06 | 18.50 | 0.53 |
| PC-TH-6-**45** | 0.5 | L | 44.40 | 35.10 | 1.26 | n/a | n/a | n/a | n/a | n/a | n/a | n/a | n/a | 16.20 | 0.46 |
| ***AVERAGE*** | ***0.67*** |  | ***46.03*** | ***33.80*** | ***1.36*** | ***24.95*** | ***35.05*** | ***32.70*** | ***1.41*** | ***1.08*** | ***19.59*** | ***33.24*** | ***52.83*** | ***17.50*** | ***0.52*** |

All lengths were measured in cm. Divarication angles were measured in degrees. All track measurements were rounded to two decimal places.

PC-TH-6 features a narrow WAP of 12 cm, shallow angles of rotation which increase from 4.6° to 5.3°, and an obtuse pace angulation angle (170.1°) (S2 Table). Pace lengths from PC-TH-6-43 (1.51 m) and PC-TH-6-44 (1.30 m) are similar to other morphotype-1a trackways. A stride of 2.80 m and ~1.84 m average hip height determined the sequence constituted a walking gait (stride/hip height ratio = 1.52) and 1.94-2.14 m/s (6.98-7.70 km/h) velocity (S3 Table). PC-TH-6-44 furthermore exhibits considerable anterior pronation relative to the track midline (24.1°) compared to left tracks (1.0°-2.3°). Due to this difference, it is possible that this sequence might not represent a single trackway but instead a set of unrelated tracks that mimic a trackway. Our ability to make a firm determination is limited because there are only three observable tracks in this sequence.

**S2 Table. Trackway measurements for PC-TH-6.**

| **Specimen** | **L / R** | **L** | **h** | **P** | λ | **WAP** | **αR** | **αL** | **γ** |
| --- | --- | --- | --- | --- | --- | --- | --- | --- | --- |
| PC-TH-6-**43** | L | 44.40 | 1.78 | 1.51 | 2.80 | n/a | 4.58 | n/a | n/a |
| PC-TH-6-**44** | R | 49.30 | 1.97 | 1.30 | n/a | 12.00 | n/a | 5.30 | 170.12 |
| PC-TH-6-**45** | L | 44.40 | 1.78 | n/a | n/a | n/a | n/a | n/a | n/a |
| ***AVERAGE*** |  | ***46.03*** | ***1.84*** | ***1.41*** | ***2.80*** | ***12.00*** | ***4.58*** | ***5.30*** | ***170.12*** |

All values were rounded to two decimal places. Total track length (L) and width of pes angulation (WAP) were measured in cm. Hip height (h), pace (P) and stride (λ) lengths were measured in m. Right or left angles of rotation (αR or αL respectively) and pace angulation (γ) were measured in degrees.

**S3 Table. The overall trackway velocity and gait ratio for PC-TH-6.**

| **Track λ** | **L / R** | **L** | **L-SD** | **h** | **λ** | **λ/h** | **AX-V** | **TR-V** | **AX-V-SD** | **TR-V-SD** |
| --- | --- | --- | --- | --- | --- | --- | --- | --- | --- | --- |
| PC-TH-6-43-45 | L | 46.03 | n/a | 1.84 | 2.80 | 1.52 | 2.14 | 1.94 | n/a | n/a |

As the trackway is composed of a single stride, standard deviations and variance were not calculated. All values were rounded to two decimal places. Track length was measured in cm. Hip height (h), and stride length (λ) were measured in metres. Velocity was measured in m/s.

**Associated tracks**

PC-TH-A-1 features three large, concave epirelief tridactyl tracks and extends over 4.7 m at 201.7° (S3 Fig, S4 Table). A presumed absent second left track in the sequence reduces the track classification to an association. The relatively shallow impressions are mostly infilled by ripples which reduced the definition of digit margins. As a result, the tracks score low preservation grades of 0-0.5 (average 0.33). Similarities to morphotype-1a include overall track lengths (40.1-44.1 cm), l/w ratios (1.27-1.40), digit iii/ii ratios >1 and digit iii/iv ratios <1, moderate mesaxony (0.48-0.49), and digit ii-iv divarication angles (49.9°-54.9°) within the 40°-60° morphotype-1a range.

**S3 Fig. Overview of PC-TH-A-1.** (A) Textured orthophoto with software-based shadowing, (B) DEM, (C) outline. Most tracks were marginally worn and partly infilled by ripples, particularly PC-TH-A-1-04 and 05.

**S4 Table. Track measurements for PC-TH-A-1.**

|  |  |  |  | |  | **Digit length (DL)** | | | **DL ratios** | | **Divarication angles** | | |  |  | |
| --- | --- | --- | --- | --- | --- | --- | --- | --- | --- | --- | --- | --- | --- | --- | --- | --- |
| **Specimen** | **PG** | **L / R** | **L** | **W** | **L/W** | **II** | **III** | **IV** | **III/II** | **III/IV** | **II-III** | **III-IV** | **II-IV** | **te** | **M** |  |
| PC-TH-A-1-**03** | 0 | L? | n/a | n/a | n/a | n/a | n/a | n/a | n/a | n/a | n/a | n/a | n/a | n/a | n/a |  |
| PC-TH-A-1-**04** | 0.5 | R? | 44.10 | 31.40 | 1.40 | 22.70 | 30.70 | 32.00 | 1.35 | 0.96 | 23.72 | 26.14 | 49.86 | 15.40 | 0.49 |  |
| PC-TH-A-1-**05** | 0.5 | R? | 40.10 | 31.50 | 1.27 | 16.30 | 23.70 | 26.80 | 1.45 | 0.88 | 26.53 | 28.34 | 54.87 | 15.20 | 0.48 |  |
| ***AVERAGE*** | ***0.33*** |  | ***42.10*** | ***31.45*** | ***1.34*** | ***19.50*** | ***27.20*** | ***29.40*** | ***1.40*** | ***0.92*** | ***25.13*** | ***27.24*** | ***52.37*** | ***15.30*** | ***0.49*** |  |

All lengths were measured in cm. Divarication angles were measured in degrees. All track measurements were rounded to two decimal places.

PC-TH-A-2 is composed of two large, shallow convex epirelief tridactyl tracks and extends over 1.55 m at 87.2° (S4-S5 Figs, S5 Table). Although both tracks appear on top of a bed 1 surface, it is unclear which precise horizon the tracks were originally impressed as the surrounding substrate no longer exists. This may have reduced the track margin definition and influenced our assignment of low preservation grades of 0.5-1. PC-TH-A-2-17 suffered considerable wear and was not measured. Despite this, morphotype-1a features such as broad, tapering digits and vague phalangeal pads on digit iii were distinguished to allow the tracks to be tentatively referred. PC-TH-A-2-18 features clearer long ungual marks and tapering digits similar to morphotype-1a. This track, however, includes features that likely result from locomotion rather than trackmaker anatomy. For example, a reduced track length (37.6 cm) and low l/w ratio (1.15) induced by sigmoidal curvature on digit iii. Despite this, the track is moderately mesaxonic (0.45) and divaricated between digits ii-iv by 48.2° – within morphotype-1a ranges.

**S4 Fig. Overview of PC-TH-A-2.** (A) Photograph cropped to model area, (B) DEM, (C) outline. The tracks are in shallow convex epirelief and appear on top of bed 1 as the original surrounding original horizon was eroded.

**S5 Fig. Overview of PC-TH-A-2-18.** (A) Photograph and (B) outline of PC-TH-A-2-18, which is missing most of its heel. Unlike most morphotype-1a tracks, such as those of PC-TH-1 and 2, PC-TH-A-2-18 exhibits distinct sigmoidal curvature on digit iii.

**S5 Table. Track measurements for PC-TH-A-2.**

|  |  |  |  | |  | **Digit length (DL)** | | | **DL ratios** | | **Divarication angles** | | |  |  |
| --- | --- | --- | --- | --- | --- | --- | --- | --- | --- | --- | --- | --- | --- | --- | --- |
| **Specimen** | **PG** | **L / R** | **L** | **W** | **L/W** | **II** | **III** | **IV** | **III/II** | **III/IV** | **II-III** | **III-IV** | **II-IV** | **te** | **M** |
| PC-TH-A-2-**17** | 0.5 | L | n/a | n/a | n/a | n/a | n/a | n/a | n/a | n/a | n/a | n/a | n/a | n/a | n/a |
| PC-TH-A-2-**18** | 1 | R | 37.60 | 32.80 | 1.15 | n/a | 23.20 | n/a | n/a | n/a | 28.40 | 19.80 | 48.20 | 14.90 | 0.45 |

All lengths were measured in cm. Divarication angles were measured in degrees. All track measurements were rounded to two decimal places.

PC-TH-A-5 consists of two successive concave epirelief tridactyl tracks and extends over 1.47 m at 42.1° (S7-S8 Figs, S6 Table). The digit margins of both tracks are worn and enlarged due to present-day erosion which results in low preservation grades of 1. Morphotype-1a similarities include 43.8-43.9 cm overall track lengths, 1.25-1.46 l/w ratios, digit iii/ii ratios >1 (1.28-1.34), digit iii/iv ratios <1 (0.86-0.87), and moderate mesaxony (0.47-0.55). The digit ii-iv divarication angle for PC-TH-A-5-60 however exceeds morphotype-1a ranges to 73.2°. Despite this, due to metrical similarities (i.e. l/w ratio, mesaxony), the tracks are referred to morphotype-1a.

**S7 Fig. Overview of PC-TH-A-5.** (A) Textured orthophoto with software-based shadowing, (B) DEM, (C) outline. The tracks have low mesaxony like morphotype-1a but overall have poorly defined margins.

**S8 Fig. Close up photographic and digital representations of PC-TH-A-5 tracks.** Photographs, outlines, contour maps, and DEMs are respectively represented from left to right. (A-D) PC-TH-A-5-60, (E-H) PC-TH-A-5-61. The tracks are Concave epirelief, with partial sediment infill on digits, and are heavily worn.

**S6 Table. Track measurements for PC-TH-A-5.**

|  |  |  |  |  |  | **Digit length (DL)** | | | **DL ratios** | | **Divarication angles** | | |  |  |
| --- | --- | --- | --- | --- | --- | --- | --- | --- | --- | --- | --- | --- | --- | --- | --- |
| **Specimen** | **PG** | **L / R** | **L** | **W** | **L/W** | **II** | **III** | **IV** | **III/II** | **III/IV** | **II-III** | **III-IV** | **II-IV** | **te** | **M** |
| PC-TH-A-5-**60** | 1 | R | 43.90 | 35.20 | 1.25 | 20.80 | 27.90 | 31.90 | 1.34 | 0.87 | 36.98 | 36.26 | 73.24 | 16.40 | 0.47 |
| PC-TH-A-5-**61** | 1 | L | 43.80 | 30.00 | 1.46 | 20.30 | 26.00 | 30.20 | 1.28 | 0.86 | 29.60 | 30.20 | 59.80 | 16.40 | 0.55 |
| ***AVERAGE*** | ***1*** |  | ***43.85*** | ***32.60*** | ***1.35*** | ***20.55*** | ***26.95*** | ***31.05*** | ***1.31*** | ***0.87*** | ***33.29*** | ***33.23*** | ***66.52*** | ***16.40*** | ***0.51*** |

All lengths were measured in cm. Divarication angles were measured in degrees. All track measurements were rounded to two decimal places.

**Additional referred morphotype-1 material figures**

**S9 Fig**. **Overview of PC-TH-3**. (A) Textured orthophoto with software-based shadowing, (B) DEM, (C) outline. Most tracks are incomplete or unexposed. PC-TH-3-21 was recognised from its heel, which resembles that of PC-TH-3-20.

**Morphotype-2:**

**PC-SA-4**

Seven heavily eroded tracks constitute a trackway, with multiple missing tracks between those present, bearing ~314.7° over 11.6 m opposite and in the same horizon as PC-SA-3. Most tracks are highlighted by partial rippled displacement rims and are likely ovular/subtriangular in shape. Due to this, tracks score preservation grades of 0 and are not used to diagnose morphotype-2.

**Additional referred morphotype-2 material figures**

**S10 Fig. Photograph and outlines of PC-SA-I-124.** (A) Photograph highlights a worn displacement rim around the track. (B) Outline highlights possible digits around the top of the track. The ripples which intersected the track have likely since eroded. Presently, a few of these ripples are faintly recognised.

**Descriptive tables of tracks**

Provided below are field observations of the tracks at Prince Charles’s Point. This specifically details: track specimen numbers; whether a track is left or right (L / R); is in concave or convex epirelief (relief); symmetric or asymmetric (symmetry); its preservation grade (PG); bed of origin (bed no.); and a general description outlining track location and notable morphologies. Note track numbers are emboldened for ease of reading.

**S7 Table. Track field observations from Prince Charles’s Point**

| **Specimen No.** | **L / R** | **Relief** | **Symmetry** | **PG** | **Bed no.** | **General description** |
| --- | --- | --- | --- | --- | --- | --- |
| PC-TH-I-**01** | R | Concave epirelief | Asymmetric | 2.0 | 2 | Seaward facing, worn, infilled complete tridactyl track with sharp ungual marks and marginally well-defined phalangeal pads - is closest to the high tide mark in section one |
| PC-TH-I-**02** | ? | Convex epirelief | ? | 0.0 | 2 | Seaward facing partial convex epirelief tridactyl - missing most of all digits and heel region. Appears on top of bed 1-2 boundary horizon, likely registered in bed 2 |
| PC-TH-A-1-**03** | L? | Concave epirelief | ? | 0.0 | 2 | Worn, partial convex epirelief tridactyl above crossing tridactyl above boulder |
| PC-TH-A-1-**04** | R? | Concave epirelief | ? | 0.5 | 2 | concave epirelief tridactyl with very worn digit margins and infilled with ripples |
| PC-TH-A-1-**05** | R? | Concave epirelief | ? | 0.5 | 2 | concave epirelief tridactyl with very worn digit margins and infilled with ripples |
| PC-TH-1-**06** | L | Concave epirelief | ? | 0.5 | 2 | Eroded, partial concave epirelief tridactyl, final visible track in PC-TH-1 trackway. Most of track floor eroded, defined from track margins of partial digit iii and ii |
| PC-TH-1-**07** | R | Concave epirelief | Asymmetric | 2.0 | 2 | Sharp concave epirelief tridactyl with minor ripple infilling around Piv1, highest most complete track. Track possesses '2:3:4' phalangeal pad configuration. Ripples clearly intrude heel region |
| PC-TH-1-**08** | L | Concave epirelief | Asymmetric | 1.5 | 2 | Concave epirelief tridactyl with digits iii-ii overprinting PC-TH-2-12, track possesses clear ungual marks and phalangeal pads on all digits. Ripples clearly intrude heel region |
| PC-TH-1-**09** | R | Concave epirelief | Asymmetric | 1.5 | 2 | Worn concave epirelief tridactyl, situated immediately left of PC-TH-2-13 |
| PC-TH-1-**10** | L | Concave epirelief | Asymmetric | 1.0 | 2 | Worn concave epirelief tridactyl commences the PC-TH-1 trackway. At centre of track, a pebble was extracted and thin sectioned |
| PC-TH-2-**11** | L | Concave epirelief | Asymmetric | 1.5 | 2 | Concave epirelief tridactyl, left of PC-TH-1-07, concludes PC-TH-2 trackway. Track possesses partial infill in digit ii and possesses clear ungual marks. Ripples clearly intrude heel region |
| PC-TH-2-**12** | R | Concave epirelief | Asymmetric | 1.5 | 2 | Concave epirelief tridactyl overprinted by PC-TH-1-08 with sharp ungual marks. Ripples clearly intrude heel region |
| PC-TH-2-**13** | L | Concave epirelief | Asymmetric | 1.5 | 2 | Worn concave epirelief tridactyl right of PC-TH-1-09. Ripples clearly intrude heel region |
| PC-TH-2-**14** | R | Concave epirelief | Asymmetric | 1.5 | 2 | Worn concave epirelief tridactyl 1m right of PC-TH-1-10. Ripples clearly intrude heel region |
| PC-TH-2-**15** | L | Convex epirelief | Asymmetric | 1.0 | 2 | Partial shallowly convex epirelief tridactyl partly impressing into sauropod displacement rim of PC-SA-2-86 pes track. Anterior margins of digits feature clearly discernible phalangeal pads, but no obvious heel |
| PC-TH-I-**16** | ? | Convex epirelief | ? | 0.5 | 2 | Partial convex epirelief tridactyl, digit iv mostly missing, only distal region intact. Situated within the gauge of PC-SA-1, between PC-SA-1-76 and 77 |
| PC-TH-A-2-**17** | L | Convex epirelief | Asymmetric | 0.5 | 2 | Heel worn off as upside down 'v' shape, visible digit ii. Situated above PC-SA-1-73. Very vague phalangeal pad margins on all digits. Track likely registered in eroded bed 2 horizon |
| PC-TH-A-2-**18** | R | Convex epirelief | Asymmetric | 1.0 | 2 | Worn convex epirelief tridactyl with pronounced sigmoidal curvature on digit iii. Track likely registered in eroded bed 2 horizon |
| PC-TH-3-**19** | R? | Concave epirelief | Asymmetric | 0.5 | 2 | Worn partial concave epirelief tridactyl, track commences PC-TH-3 trackway. Digit iv is missing |
| PC-TH-3-**20** | L? | Convex epirelief | Asymmetric | 1.5 | 2 | Most marginally well-defined track in trackway - a shallow Convex epirelief tridactyl with clear displacement rims and pronounced 'U' shape heel region |
| PC-TH-3-**21** | R? | ? | ? | 0.0 | 2 | Heel only visible, rest of track under thin Bed 3 layer |
| PC-TH-3-**22** | L? | Convex epirelief | ? | 0.5 | 2 | Convex epirelief tridactyl - lateral digits only - tapering to worn ungual marks. Final track in PC-TH-3 sequence |
| PC-TH-4-**23** | R | Convex epirelief | Asymmetric | 0.5 | 2 | Starts visible PC-TH-4 trackway. A convex epirelief tridactyl. Heel region eroded since 2022. Worn digit margins and ungual marks. Smaller PC-TH-I-63 is opposite |
| PC-TH-4-**24** | R | Concave epirelief | Asymmetric | 1.0 | 2 | Right deep concave epirelief tridactyl with rounded digit margins |
| PC-TH-4-**25** | L | Concave epirelief | Asymmetric | 1.0 | 2 | Left deep concave epirelief tridactyl, track margins rounded, medial slightly infilled |
| PC-TH-4-**26** | R | Concave epirelief | Asymmetric | 0.5 | 2 | Right shallow concave epirelief tridactyl, track margins rounded, digit iii worn |
| PC-TH-4-**27** | L | Concave epirelief | Asymmetric | 0.5 | 2 | Left shallow concave epirelief tridactyl, digit margins rounded |
| PC-TH-4-**28** | R | Concave epirelief | Asymmetric | 1.0 | 2 | Right shallow concave epirelief tridactyl, digit margins rounded |
| PC-TH-4-**29** | L | Concave epirelief | Asymmetric | 1.0 | 2 | Left shallow concave epirelief tridactyl, digit margins rounded |
| PC-TH-4-**30** | R | Concave epirelief | ? | 0.0 | 2 | Indistinct concave epirelief underprint, circular in shape like a small sauropod pes - lowermost track in sequence. Distally terminating region of lateral digits vaguely visible. Last visible track in trackway |
| PC-TH-5-**31** | L | Concave epirelief | Asymmetric | 0.5 | 2 | Starts the PC-TH-5 trackway. Left shallow concave epirelief tridactyl, track margin rounded, digit iii infilled |
| PC-TH-5-**32** | R | Concave epirelief | Asymmetric | 0.5 | 2 | Right shallow concave epirelief tridactyl, track margins rounded, slight infilling |
| PC-TH-5-**33** | L | Concave epirelief | Asymmetric | 1.0 | 2 | Left Concave epirelief tridactyl, digit margins rounded, clearer than previous two tracks |
| PC-TH-5-**34** | R | Concave epirelief | Asymmetric | 1.0 | 2 | Right concave epirelief tridactyl, digit margins rounded, partly infilled |
| PC-TH-5-**35** | L | Concave epirelief | Asymmetric | 1.0 | 2 | Left infilled concave epirelief tridactyl, features visibly wide digit iv divarication |
| PC-TH-5-**36** | R | Concave epirelief | Asymmetric | 1.0 | 2 | Right infilled concave epirelief tridactyl, underprint hard to see |
| PC-TH-5-**37** | L | Concave epirelief | Asymmetric | 1.0 | 2 | Left infilled concave epirelief tridactyl with very widely divaricated digit iv |
| PC-TH-5-**38** | L | Concave epirelief | Asymmetric | 0.5 | 2 | Left infilled concave epirelief tridactyl, most digits are not easily distinguished as a result of infill |
| PC-TH-5-**39** | R | Convex epirelief | Asymmetric | 0.0 | 2 | Partial convex epirelief, digit iii partly worn and digit iv missing, dimensions exaggerated by substrate |
| PC-TH-5-**40** | R | Concave epirelief | Asymmetric | 1.5 | 2 | Last track in PC-TH-5 sequence, a shallow concave epirelief tridactyl with sharp digit margins, ungual marks, phalangeal pads, but with ripple infiltration throughout |
| PC-TH-I-**41** | ? | Convex epirelief | Asymmetric | 2.0 | 2 | Sharply defined convex epirelief tridactyl, digit iii under boulder. Digit margins sharply defined, ungual marks pronounced with some rounding. A worn digit iv metatarsophalangeal pad may be present. Lateral digits possess sigmoidal curvature |
| PC-TH-I-**42** | ? | Convex epirelief | ? | 0.0 | 2 | Surface worn convex epirelief track with very poorly defined digits, faces almost 90 degrees to PC-TH-I-41 |
| PC-TH-6-**43** | L | Concave epirelief | Asymmetric | 0.5 | 2 | Worn complete concave epirelief tridactyl, partially infilled, difficult to distinguish |
| PC-TH-6-**44** | R | Convex epirelief | Asymmetric | 1.0 | 2 | Convex epirelief tridactyl with worn digit margins. Phalangeal pads clearest on anterior most regions of digits. Ungual mark clearest on digit iii |
| PC-TH-6-**45** | L | Convex epirelief | ? | 0.5 | 2 | Convex epirelief tridactyl with more worn digit margins than preceding track, some Concave epirelief around infill. Distal ends of lateral digits rounded, digit iii missing clear ungual mark |
| PC-TH-I-**46** | L? | Convex epirelief | Asymmetric | 1.0 | 2 | Convex epirelief tridactyl with worn digit margins - right lateral digit is hard to distinguish, unidentifiable bone fragments in heel region |
| PC-TH-A-3-**47** | R | Convex epirelief | Asymmetric | 1.5 | 2 | Lowermost of PC-TH-A-3 of shallow convex epirelief tridactyl - slender digited with short, triangular ungual marks |
| PC-TH-A-3-**48** | L | Convex epirelief | Asymmetric | 1.5 | 2 | Uppermost of PC-TH-A-3 of shallow convex epirelief tridactyl. Following digits possess interdigital creases: digit ii (all pads), digit iii (Piii3), digit iv (Piv1). Phalangeal pad formula = '2:3:4' |
| PC-TH-A-4-**49** | L | Concave epirelief | Asymmetric | 1.5 | 2 | Shallow concave epirelief with infilling in digit iii, faces the sea and is approximately halfway between PC-TH-6 and PC-TH-4. Heel region intruded by ripples but margin still distinguishable. Digits possess some interdigital creases and sharp ungual marks |
| PC-TH-A-4-**50** | R | Convex epirelief | Asymmetric | 1.0 | 2 | Worn convex epirelief tridactyl with divergent lateral digits, digit iv missing clear ungual mark |
| PC-TH-I-**51** | ? | Concave epirelief | ? | 0.0 | 2 | Worn convex epirelief tridactyl, sole and heel missing |
| PC-TH-I-**52** | ? | Convex epirelief | ? | 0.0 | 2 | Digit iii distal tip only |
| PC-TH-I-**53** | ? | Concave epirelief | Asymmetric | 0.5 | 2 | Partially infilled tridactyl, smaller than other surrounding tracks - opposite PC-TH-I-49. Track lacks clear ungual marks or phalangeal pads - rounded |
| PC-TH-I-**54** | ? | Concave epirelief | Asymmetric | 0.5 | 2 | Digit iii clearest, possibly digit ii but unclear overall - positioned 2 o'clock to PC-TH-A-3-48, identified via DEM and seen in field |
| PC-TH-I-**55** | R | Concave epirelief | Asymmetric | 1.5 | 2 | Concave epirelief tetradactyl, with motion distorted digits. Track is situated between section one and two clusters. Digit i is mostly a ungual mark impression, the digit lacks phalangeal pads. Lateral digits widely divaricated |
| PC-TH-I-**56** | L | Concave epirelief | Asymmetric | 1.5 | 2 | Concave epirelief tridactyl, below igneous intrusion and section 2 cluster. Track digit margins clear with ripples intruding. Digit iv metatarsophalangeal pad is largest pad |
| PC-TH-I-**57** | ? | Convex epirelief | ? | 0.5 | 2 | Partial convex epirelief, most southerly tridactyl opposite big igneous intrusion. Right lateral digit is partial. Track size may be exaggerated |
| PC-TH-I-**58** | ? | Convex epirelief | Asymmetric | 0.5 | 2 | Worn isolated tridactyl with rounded track margins near PC-TH-I-59 |
| PC-TH-I-**59** | L | Convex epirelief | Asymmetric | 2.0 | 2 | Isolated, sharply defined track, best at locality, but considerably surface worn |
| PC-TH-A-5-**60** | R | Concave epirelief | Asymmetric | 1.0 | 2 | Worn tridactyl above the PC-SA-4 trackway, digits ii and iv are in concave epirelief, digit iii has some infilling |
| PC-TH-A-5-**61** | L | Concave epirelief | Asymmetric | 1.0 | 2 | Worn tridactyl proceeding PC-TH-A-5-60 with poorly defined digit margins |
| PC-TH-I-**62** | ? | Concave epirelief | Asymmetric | 0.0 | 2 | Worn concave epirelief tridactyl in section two with poorly defined digit margins - track appears isolated relative to others in section two |
| PC-TH-I-**63** | ? | Convex epirelief | ? | 0.5 | 2 | Immediately left of PC-TH-4-23, small partial convex epirelief with rounded digits (lacks ungual marks) |
| PC-TH-I-**64** | ? | Concave epirelief | Asymmetric | 1.0 | 2 | Isolated concave epirelief tridactyl on slipway |
| PC-TH-I-**65** | ? | Convex epirelief | Asymmetric | 0.5 | 2 | Near PC-TH-I-59 in same bed not too far south. Digits are moderately slender with phalangeal pads (left lateral digit is missing, right lateral partial) |
| PC-U-A-1-**66** | ? | Concave epirelief | ? | 0.5 | 5 | Bed 5 track, most exposed and complete example, above tideline in cliff exposure. Narrow displacement rims encircle track |
| PC-U-A-1-**67** | ? | Concave epirelief | ? | 0.0 | 5 | Partial bed 5 track, opposite PC-U-A-1-66 in 'cliff', identified via narrow displacement rims |
| PC-SA-1-**68** | L? | Convex epirelief | ? | 0.0 | 2 | Pes? - nearest to high tide line |
| PC-SA-1-**69** | R | Convex epirelief | Asymmetric | 1.0 | 2 | Manus - last in sequence - appears impressed into bed 1 surface and may be undertrack |
| PC-SA-1-**70** | R | Convex epirelief | ? | 0.0 | 2 | Pes - last in sequence - appears impressed into bed 1 surface and may be undertrack. Track margin poorly defined |
| PC-SA-1-**71** | L | Convex epirelief | ? | 0.5 | 2 | Manus |
| PC-SA-1-**72** | L | Convex epirelief | ? | 0.5 | 2 | Pes |
| PC-SA-1-**73** | R | Convex epirelief | ? | 0.0 | 2 | Manus - only inwardly rotated manus in sequence |
| PC-SA-1-**74** | R | Convex epirelief | ? | 0.5 | 2 | Pes - poorly defined track margin - only posterior margin most well defined |
| PC-SA-1-**75** | L | Convex epirelief | Asymmetric | 1.0 | 2 | Manus - last impression directly observed in bed 2 |
| PC-SA-1-**76** | L | Convex epirelief | Asymmetric | 0.5 | 2 | Pes - last impression directly observed in bed 2 |
| PC-SA-1-**77** | R | Convex epirelief | ? | 0.5 | 2 | Manus - overimpressed by pes |
| PC-SA-1-**78** | R | Convex epirelief | Asymmetric | 0.5 | 2 | Pes |
| PC-SA-1-**79** | L | Convex epirelief | Asymmetric | 1.5 | 2 | Manus - clear track margins with pronounced posterolaterally oriented pollex |
| PC-SA-1-**80** | L | Convex epirelief | Asymmetric | 0.5 | 2 | Pes |
| PC-SA-1-**81** | R | Convex epirelief | ? | 0.5 | 2 | Manus |
| PC-SA-1-**82** | R | Convex epirelief | Asymmetric | 1.0 | 2 | Pes - only track with visible digits in sequence. Digit i missing ungual mark, other digits have ungual marks - anterolaterally oriented |
| PC-SA-1-**83** | L | Convex epirelief | Asymmetric | 1.0 | 2 | Manus - first manus in sequence, with small pollex, partly overimpressed by pes |
| PC-SA-1-**84** | L | Convex epirelief | Asymmetric | 0.5 | 2 | Pes - first in sequence |
| PC-SA-2-**85** | R | Concave epirelief | Asymmetric | 1.0 | 2 | Pes – in concave epirelief with pronounced mounded displacement rim with ripples. PC-TH-2-15 partially impressed into this track’s displacement rim |
| PC-SA-2-**86** | R | Concave epirelief | Asymmetric | 0.5 | 2 | Manus - directly below PC-TH-2-14 and overprinted by PC-SA-2-85 |
| PC-SA-2-**87** | L | Convex epirelief | Asymmetric | 0.0 | 2 | Pes – in convex epirelief, below PC-TH-1-10, with toe-like bulges on side |
| PC-SA-2-**88** | R | Concave epirelief | Asymmetric | 1.0 | 2 | Pes - features a deep concave epirelief heel pad |
| PC-SA-2-**89** | R | Convex epirelief | ? | 0.0 | 2 | Manus - overprinted by PC-SA-2-88 |
| PC-SA-2-**90** | L | Convex epirelief | Asymmetric | 0.5 | 2 | Pes - may feature pedal impression and Concave epirelief heel pad |
| PC-SA-I-**91** | ? | Concave epirelief | ? | 0.5 | 2 | Isolated amongst boulders near slipway, probably a pes |
| PC-SA-I-**92** | ? | Concave epirelief | ? | 0.0 | 2 | Displacement clear only on two sides, foot floor worn off, situated on digit margins of slipway |
| PC-U-A-2-**93** | R | Concave epirelief | Asymmetric | 0.5 | 5 | On bed 5 below PC-TH-1/2, boulder was removed from atop (not in view of drone imagery |
| PC-U-A-2-**94** | L | Concave epirelief | Asymmetric | 0.5 | 5 | Small rounded track, no clear displacement - unclear on imagery |
| PC-U-A-2-**95** | R | Concave epirelief | Asymmetric | 0.5 | 5 | Manus track? Wider than long |
| PC-U-A-3-**96** | ? | Concave epirelief | Asymmetric | 0.5 | 5 | Most sauropod pes like in shape, displacement on lower right |
| PC-U-A-3-**97** | ? | Concave epirelief | Asymmetric | 0.5 | 5 | Pes? On digit margins of platform, displacement on left side only |
| PC-U-A-4-**98** | ? | Concave epirelief | ? | 0.0 | 5 | Half a manus print? With mounded displacement rim |
| PC-U-A-4-**99** | ? | Convex epirelief | Asymmetric | 0.5 | 5 | Pes? above PC-U-A-4-99, displacement unclear |
| PC-U-A-4-**100** | ? | Convex epirelief | ? | 0.5 | 5 | Pes type track top left of PC-U-A-4-99, no displacement |
| PC-SA-I-**101** | L? | Convex epirelief | Asymmetric | 1.0 | 2 | Manus track, faces opposite direction to SA-3 trackway |
| PC-SA-3-**102** | L | Convex epirelief | Asymmetric | 0.5 | 2 | Pes |
| PC-SA-3-**103** | R | Convex epirelief | ? | 0.5 | 2 | Manus - overimpressed by pes |
| PC-SA-3-**104** | R | Convex epirelief | Asymmetric | 0.5 | 2 | Pes |
| PC-SA-3-**105** | L | Convex epirelief | Asymmetric | 0.5 | 2 | Pes - manus overimpressed |
| PC-SA-3-**106** | R | Convex epirelief | Asymmetric | 0.5 | 2 | Pes |
| PC-SA-3-**107** | L | Convex epirelief | ? | 0.5 | 2 | Manus - overimpressed by pes |
| PC-SA-3-**108** | L | Convex epirelief | Asymmetric | 1.0 | 2 | Pes – overimpressed pes from SA-A-5 |
| PC-SA-3-**109** | R | ? | ? | n/a | 2 | Pes? - under boulder, can see outline in the field |
| PC-SA-3-**110** | L | Convex epirelief | Asymmetric | 1.0 | 2 | Pes |
| PC-SA-3-**111** | R | Convex epirelief | Asymmetric | 1.5 | 2 | Pes - morphologically the clearest track with distinct digits, first track in sequence. Track also features clear mounded displacement rim with ripples across its surface – note the widest widths are recorded laterally to the track |
| PC-SA-A-1-**112** | L | Convex epirelief | ? | 0.5 | 2 | Pes? – directly behind boulder |
| PC-SA-A-1-**113** | L | Convex epirelief | Asymmetric | 1.0 | 2 | Manus - below boulder |
| PC-SA-A-1-**114** | R | Convex epirelief | ? | 0.0 | 2 | Pair? - overimpressed by PC-SA-3-108 |
| PC-SA-I-**115** | ? | Convex epirelief | ? | 0.0 | 2 | Uppermost sauropod track in section two |
| PC-SA-I-**116** | ? | Convex epirelief | Asymmetric | 0.5 | 2 | Pes? - isolated track immediately right of PC-TH-A-4 |
| PC-SA-4-**117** | ? | Concave epirelief | ? | 0.0 | 2 | Partial pes? Between lower most tracks of PC-TH-6 and PC-TH-A-4 |
| PC-SA-4-**118** | ? | Concave epirelief | ? | 0.0 | 2 | Partial pes? Opposite left of PC-TH-6-45 |
| PC-SA-4-**119** | ? | Concave epirelief | ? | 0.0 | 2 | Indistinct underprint, circular in shape above boulder |
| PC-SA-4-**120** | ? | Concave epirelief | ? | 0.0 | 2 | Partial track, one side visible |
| PC-SA-4-**121** | ? | Concave epirelief | ? | 0.0 | 2 | Indistinct track, only displacement rim remains visible |
| PC-SA-4-**122** | ? | Convex epirelief | ? | 0.0 | 2 | Partial pes? Above isolated manus next to PC-SA-3 |
| PC-SA-4-**123** | ? | Concave epirelief | ? | 0.0 | 2 | Worn track with poor track definition |
| PC-SA-I-**124** | ? | Convex epirelief | Asymmetric | 1.0 | 2 | Worn pes? Digits may be visible. Track drawn and figured in Andrews (1991)? |
| PC-SA-I-**125** | ? | Convex epirelief | Asymmetric | 0.0 | 2 | Crosscut pes? Situated a few metres north of PC-SA-I-125. Possibly associated with PC-SA-5? |
| PC-SA-I-**126** | R? | Concave epirelief | Asymmetric | 0.5 | 2 | Pes? - Precedes PC-SA-5 trackway, possibly associated |
| PC-SA-5-**127** | R | Convex epirelief | Asymmetric | 1.5 | 2 | Pes - only track with visible digits in sequence. Digit i anterolaterally oriented in opposite direction to digits ii-iv. Digits are triangular shaped with ungual marks. Heel pad present and possible pedal impression |
| PC-SA-5-**128** | L | Convex epirelief | Asymmetric | 1.0 | 2 | Pes - partially worn track margin |
| PC-SA-5-**129** | R | Convex epirelief | Asymmetric | 0.5 | 2 | Pes - partially worn track margin and displacement rim |
| PC-SA-A-2-**130** | ? | Concave epirelief | ? | 0.0 | 2 | Displacement rim only, lowermost |
| PC-SA-A-2-**131** | ? | Concave epirelief | ? | 0.0 | 2 | Displacement rim only, uppermost. |
