# Supplementary figures and images for: A new Middle Jurassic lagoon margin assemblage of theropod and sauropod dinosaur trackways from the Isle of Skye, Scotland
Source: PLoS One. 2025 Apr 2;20(4):e0319862. doi: 10.1371/journal.pone.0319862 (PMC11964282; doi:10.1371/journal.pone.0319862)

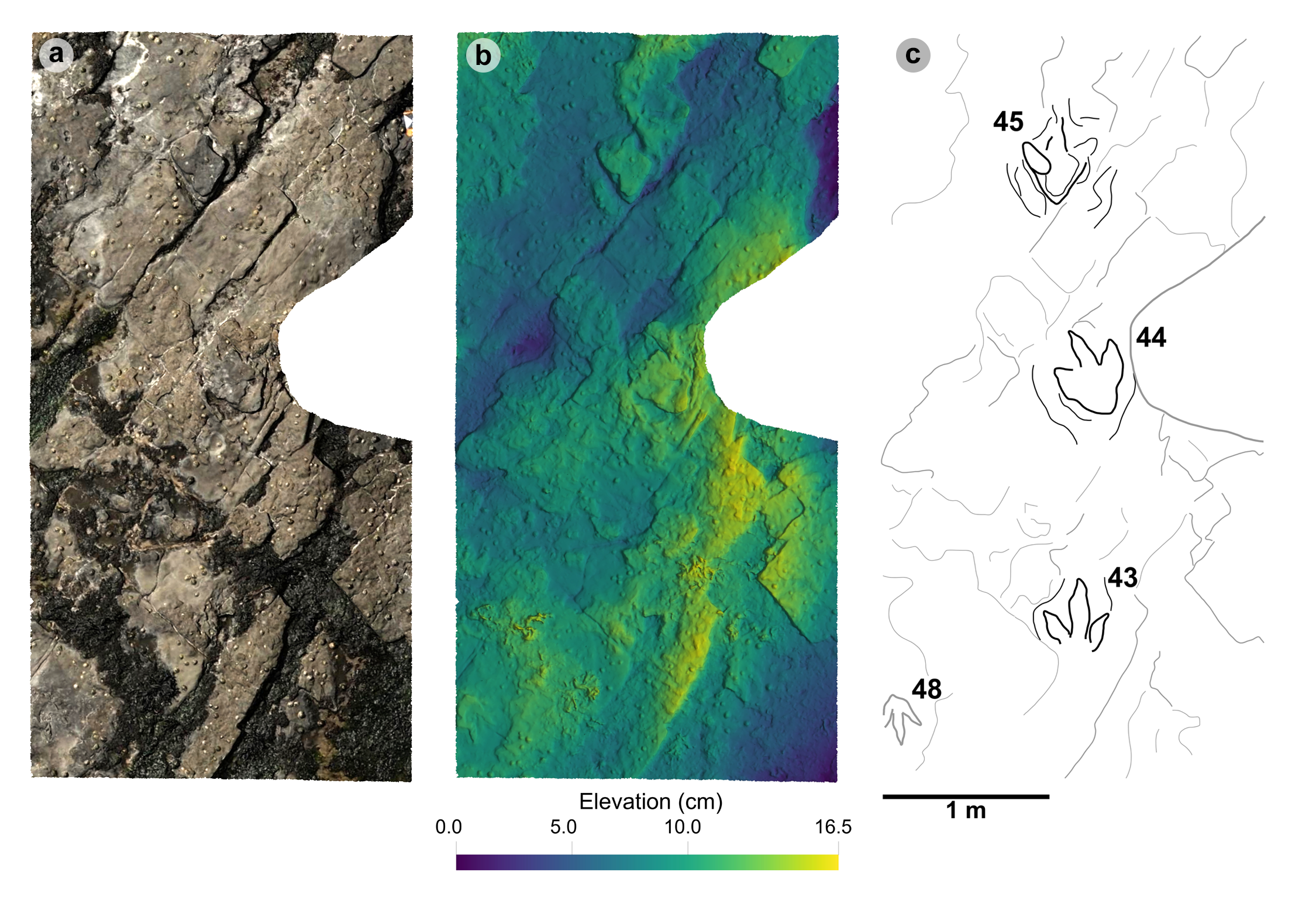

Supplement: S1 Fig — (A) Textured orthophoto with software-based shadowing, (B) DEM, (C) outline highlights the trackway. The tracks exhibit mixed preservation: PC-TH-6-43 is a concave epirelief track with poorly defined margins, while PC-TH-6-44 and 45 are in convex epirelief. (TIF) [file pone.0319862.s002.tif]

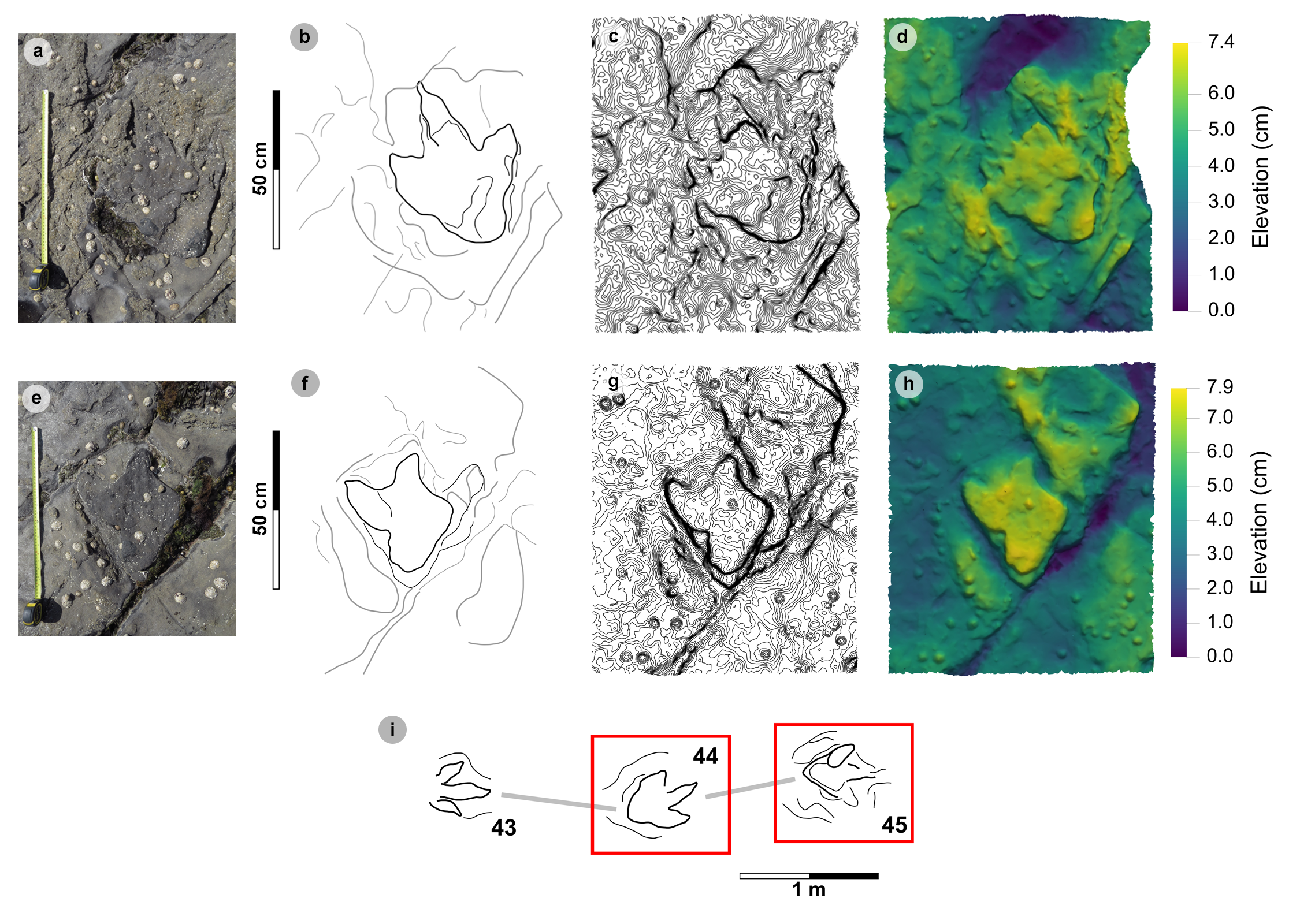

Supplement: S2 Fig — Photographs, outlines, contour maps, and DEMs are respectively represented from left to right. (A-D) Although the digit margins of PC-TH-6-44 are indistinct, unlike PC-TH-6-45, the anterior most phalangeal pads are most visible on digits iii-iv. (E-H) PC-TH-6-45 features poor digit margin definition and missing diagnostic morphologies such as phalangeal pads. The original surrounding substrate has been mostly eroded. (I) Selected tracks, indicated by red boxes, in context to the rest of the trackway. (TIF) [file pone.0319862.s003.tif]

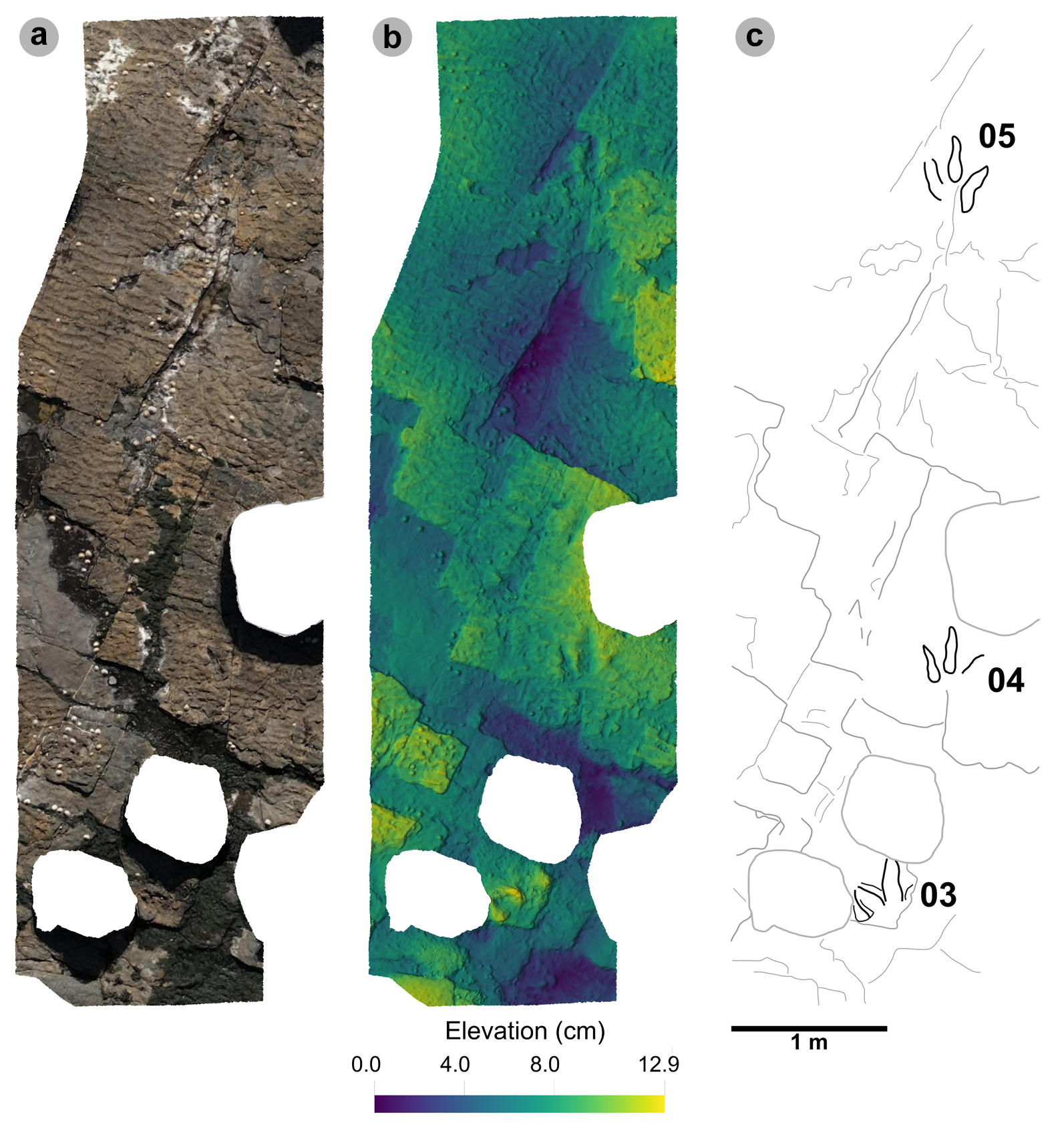

Supplement: S3 Fig — (A) Textured orthophoto with software-based shadowing, (B) DEM, (C) outline. Most tracks were marginally worn and partly infilled by ripples, particularly PC-TH-A-1-04 and 05. (TIF) [file pone.0319862.s004.tif]

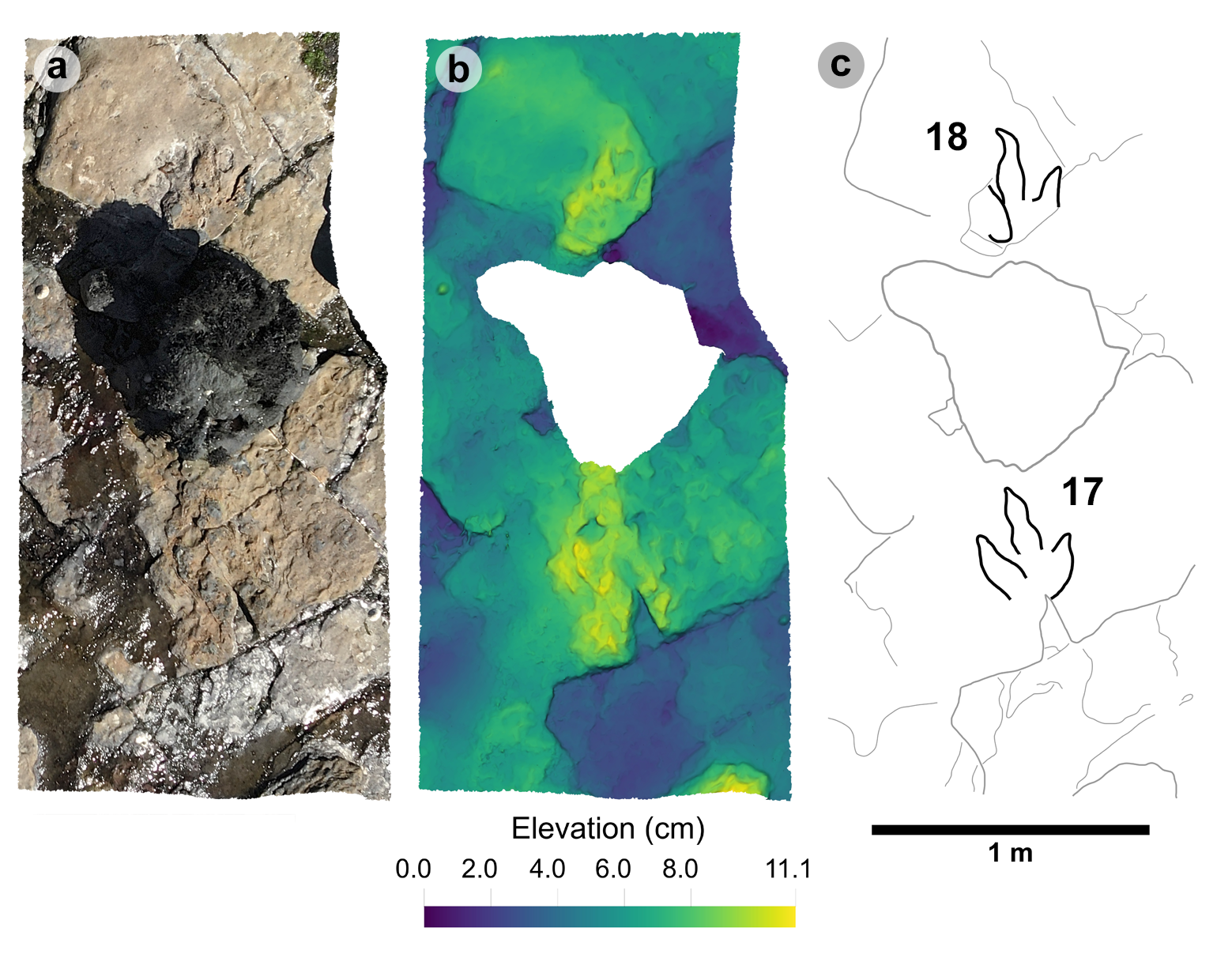

Supplement: S4 Fig — (A) Photograph cropped to model area, (B) DEM, (C) outline. The tracks are in shallow convex epirelief and appear on top of bed 1 as the original surrounding original horizon was eroded. (TIF) [file pone.0319862.s005.tif]

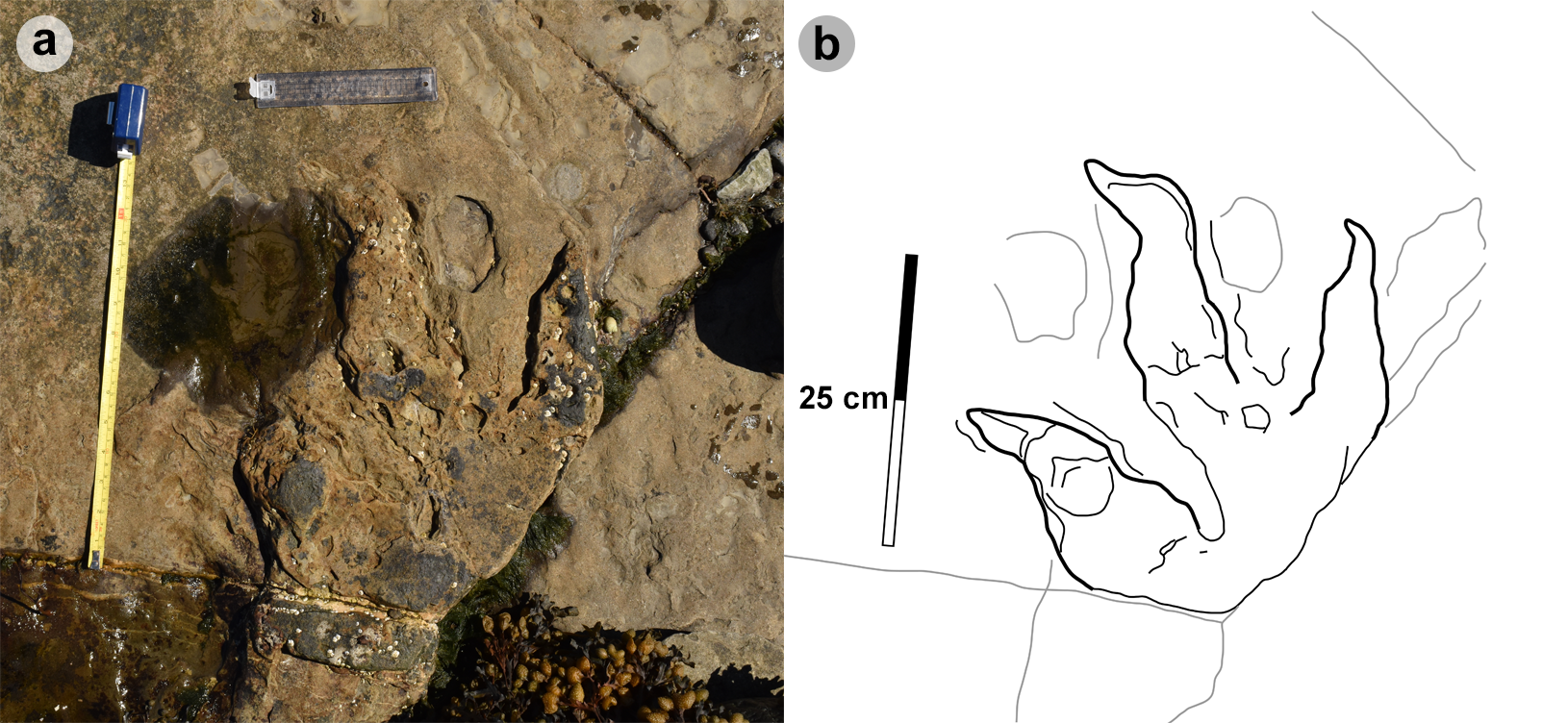

Supplement: S5 Fig — (A) Photograph and (B) outline of PC-TH-A-2-18, which is missing most of its heel. Unlike most morphotype-1a tracks, such as those of PC-TH-1 and 2, PC-TH-A-2-18 exhibits distinct sigmoidal curvature on digit iii. (TIF) [file pone.0319862.s006.tif]

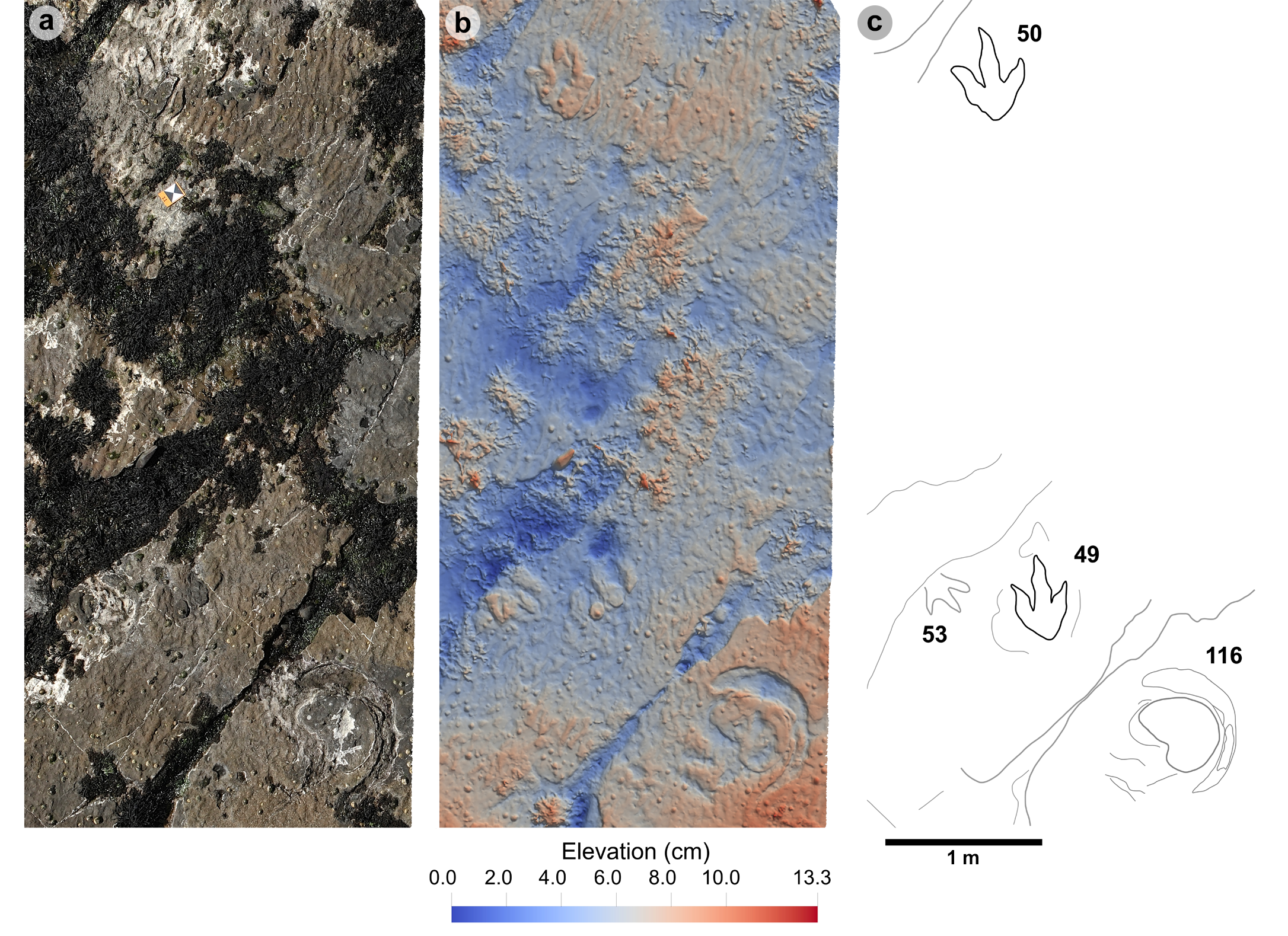

Supplement: S6 Fig — (A) Photograph cropped to model, (B) DEM, (C) outline. The tracks are almost perpendicular to the long axis of the ripples. (TIF) [file pone.0319862.s007.tif]

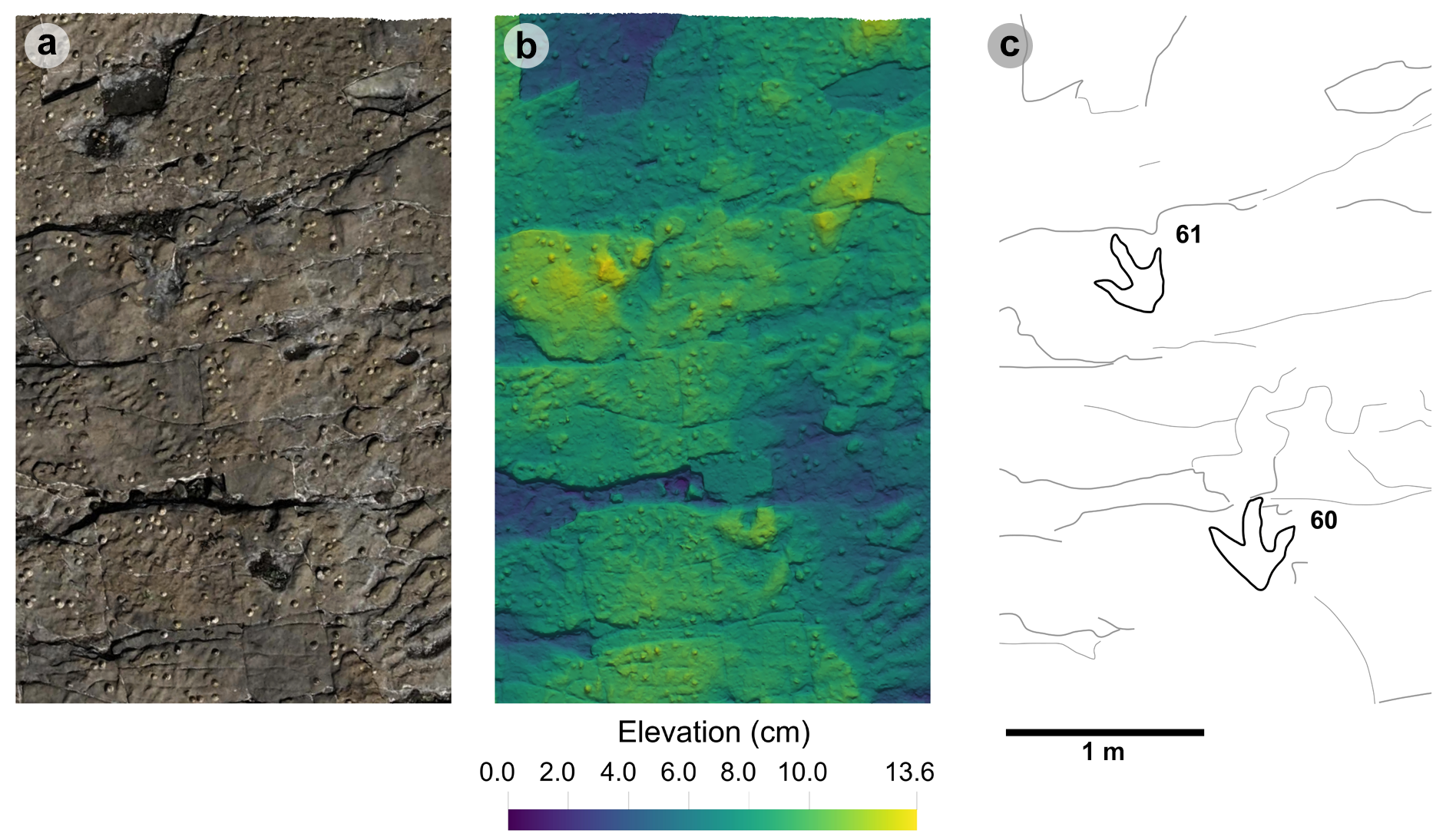

Supplement: S7 Fig — (A) Textured orthophoto with software-based shadowing, (B) DEM, (C) outline. The tracks have low mesaxony like morphotype-1a but overall have poorly defined margins. (TIF) [file pone.0319862.s008.tif]

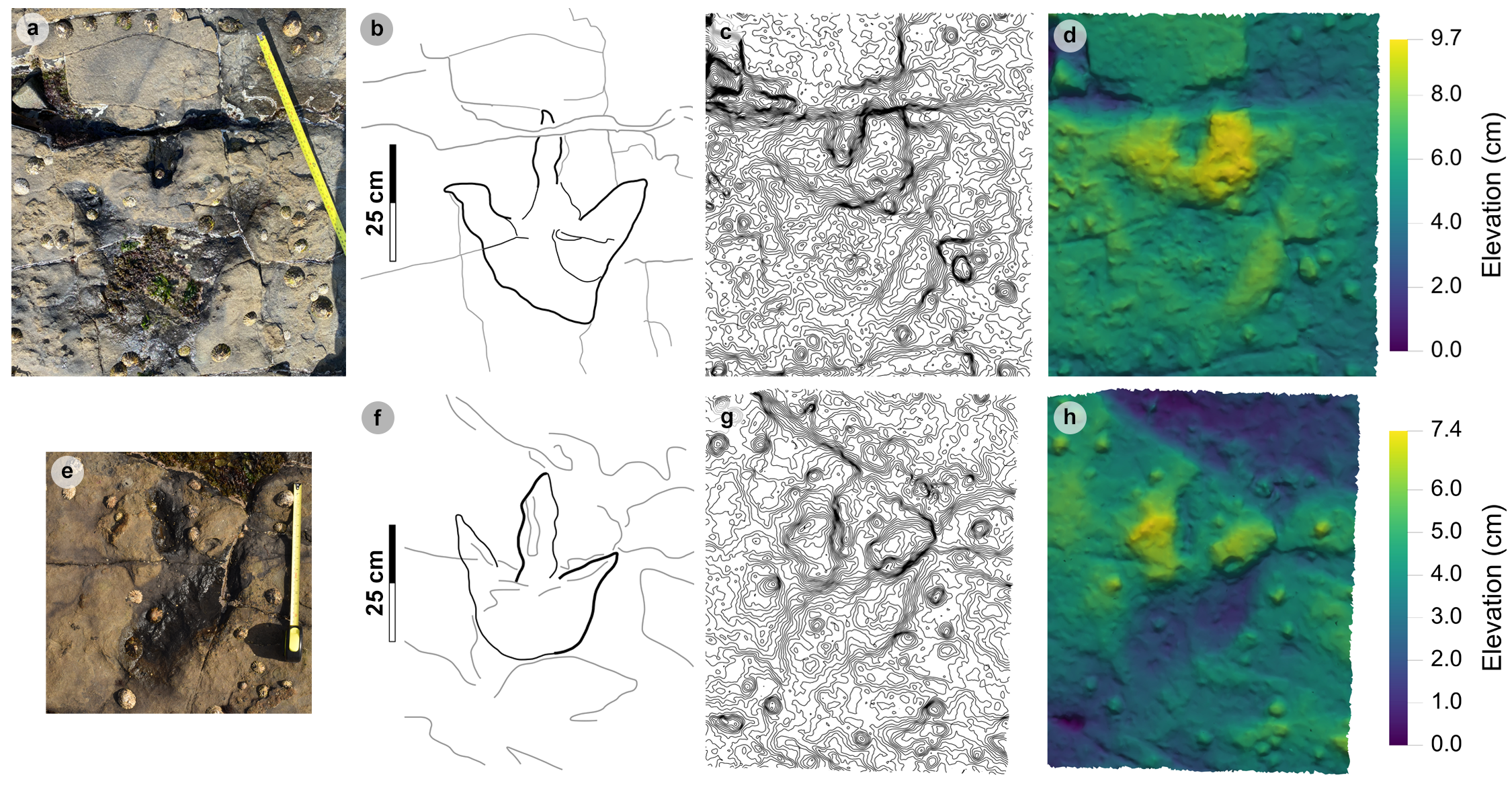

Supplement: S8 Fig — Photographs, outlines, contour maps, and DEMs are respectively represented from left to right. (A-D) PC-TH-A-5-60, (E-H) PC-TH-A-5-61. The tracks are in concave epirelief, with partial sediment infill on digits, and are heavily worn. (TIF) [file pone.0319862.s009.tif]

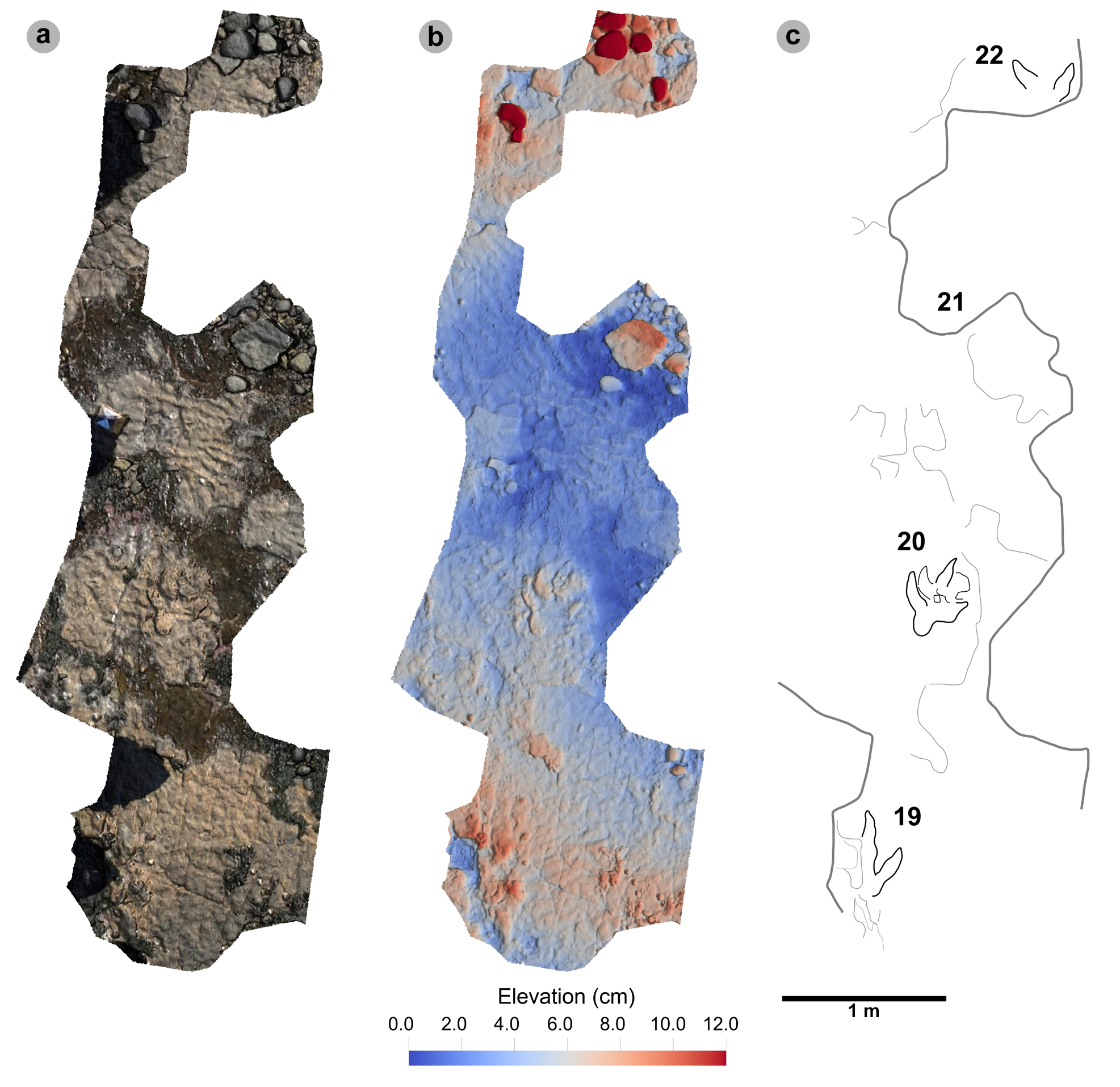

Supplement: S9 Fig — (A) Textured orthophoto with software-based shadowing, (B) DEM, (C) outline. Most tracks are incomplete or unexposed. PC-TH-3-21 was recognised from its heel, which resembles that of PC-TH-3-20. (TIF) [file pone.0319862.s010.tif]

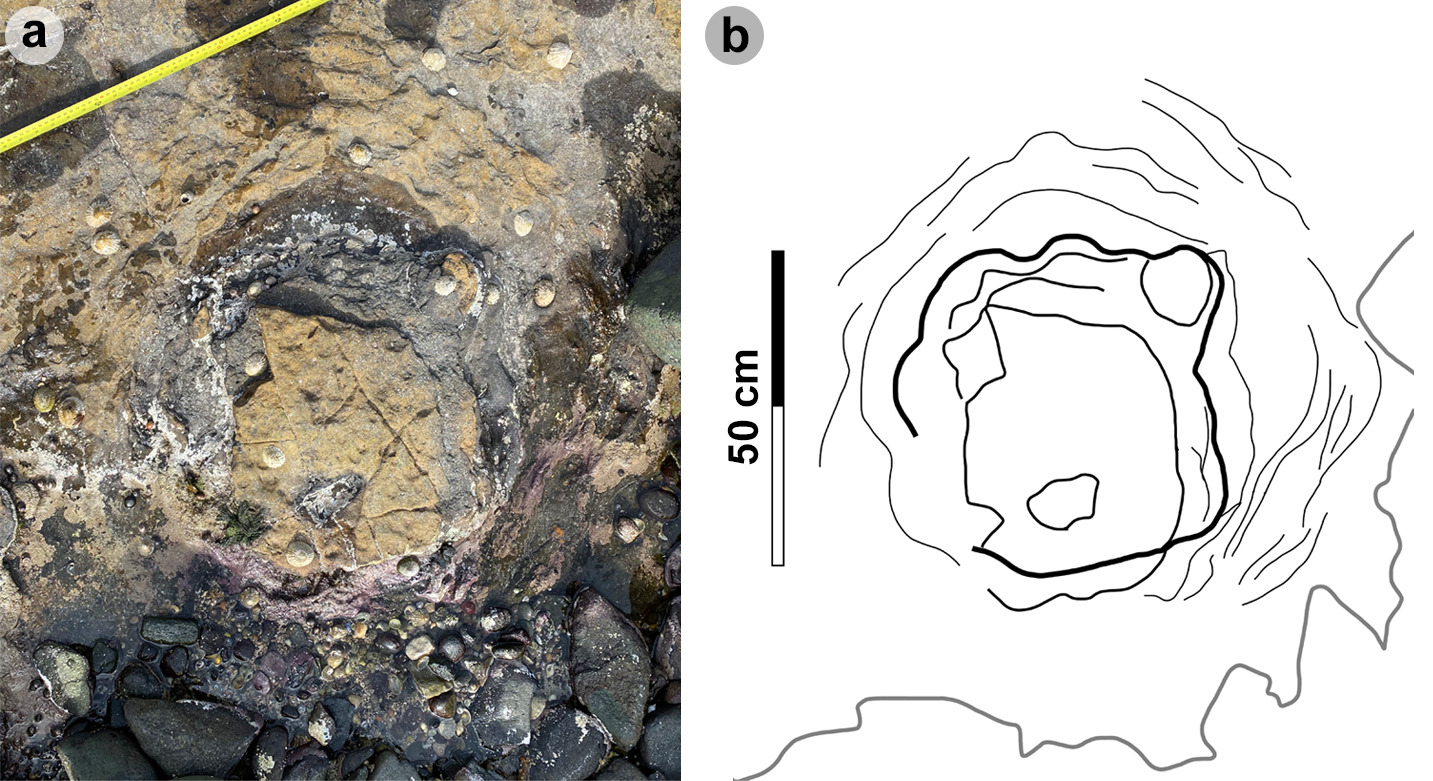

Supplement: S10 Fig — (A) Photograph highlights a worn displacement rim around the track. (B) Outline highlights possible digits around the top of the track. The ripples which intersected the track have likely since eroded. Presently, a few of these ripples are faintly recognised. (TIF) [file pone.0319862.s011.tif]
